# Supplementary material for: Bayesian spatial modelling of localised SARS-CoV-2 transmission through mobility networks across England
Source: PLoS Comput Biol. 2023 Nov 13;19(11):e1011580. doi: 10.1371/journal.pcbi.1011580 (PMC10756685; doi:10.1371/journal.pcbi.1011580)
Supplement: S1 Text — Fig A1. Comparisons of the estimates from the Naive estimator, and the BYM2. Fig A2. Plots of the true value against the fitted value for the Naive estimator and BYM2. Fig A3. ROC curve of L1 error for the Naive estimate and BYM2. Fig A4. Comparisons of the estimates from the Naive estimator, and the BYM2. Fig A5. Plots of the true value against the fitted value for the Naive estimator and BYM2. Fig A6. ROC curve of L1 error for the Naive estimate and BYM2. Fig A7. Comparisons of the estimates from the Naive estimator, and the BYM2. Fig A8. Plots of the true value against the fitted value for the Naive estimator and BYM2. Fig A9. ROC curve of L1 error for the Naive estimate and BYM2. Fig A10. Comparisons of the estimates from the Naive estimator, and the BYM2. Fig A11. Plots of the true value against the fitted value for the Naive estimator and BYM2. Fig A12. ROC curve of L1 error for the Naive estimate and BYM2. Fig A13. Comparisons of the estimates from the Naive estimator, and the BYM2. Fig A14. Plots of the true value against the fitted value for the Naive estimator and BYM2. Fig A15. ROC curve of L1 error for the Naive estimate and BYM2. Fig A16. Comparisons of the estimates from the Naive estimator, and the BYM2. Fig A17. Plots of the true value against the fitted value for the Naive estimator and BYM2. Fig A18. ROC curve of L1 error for the Naive estimate and BYM2. Fig A19. Comparisons of the estimates from the Naive estimator, and the BYM2. Fig A20. Plots of the true value against the fitted value for the Naive estimator and BYM2. Fig A21. ROC curve of L1 error for the Naive estimate and BYM2. Fig A22. Comparisons of the estimates from the Naive estimator, and the BYM2. Fig A23. Plots of the true value against the fitted value for the Naive estimator and BYM2. Fig A24. ROC curve of L1 error for the Naive estimate and BYM2. Fig A25. Comparisons of the estimates from the Naive estimator, and the BYM2. Fig A26. Plots of the true value against the fitted value f [file pcbi.1011580.s001.docx]

**Results – Scenario 1:**


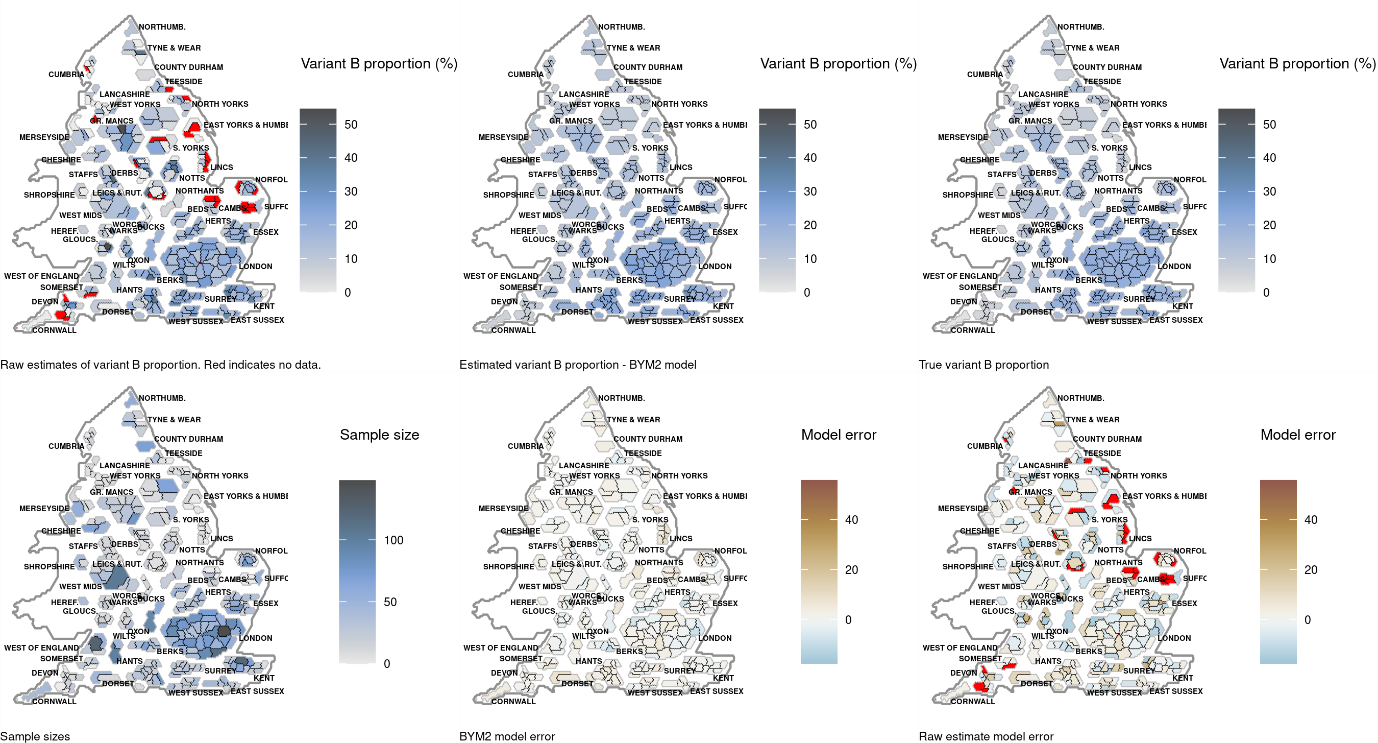


Fig A1. Comparisons of the estimates from the Naive estimator, and the BYM2


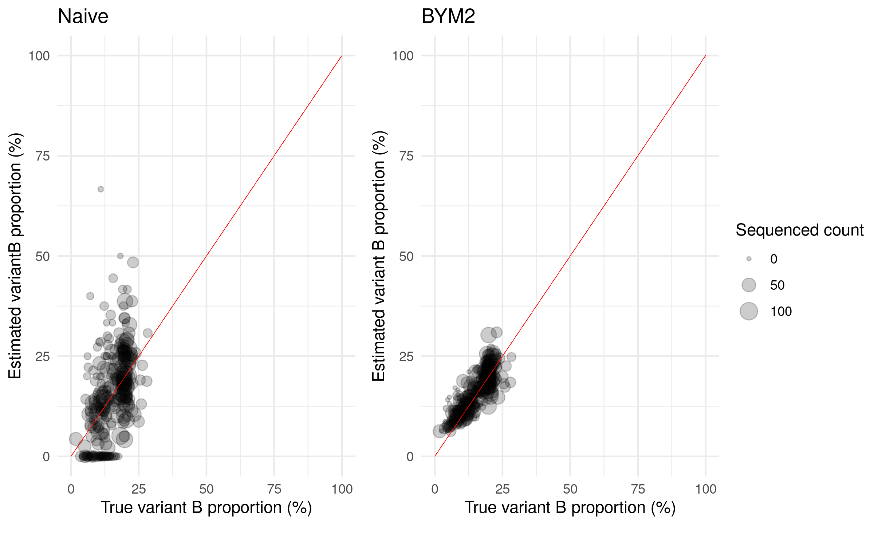


Fig A2. Plots of the true value against the fitted value for the Naive estimator and BYM2


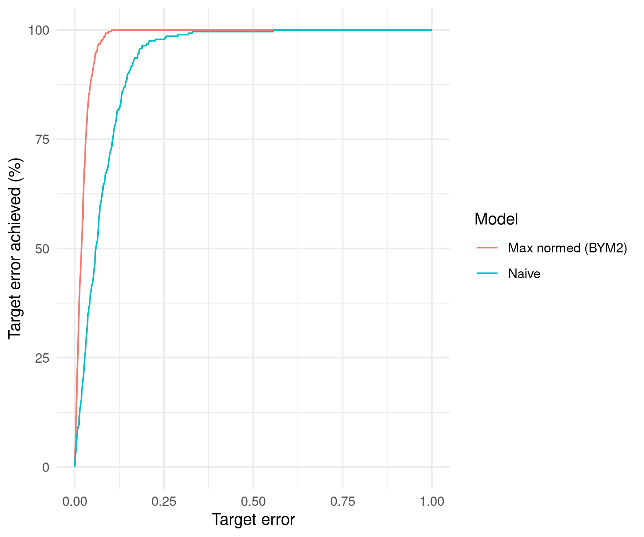


Fig A3. ROC curve of L1 error for the Naive estimate and BYM2

**Results – Scenario 1.1:**

**
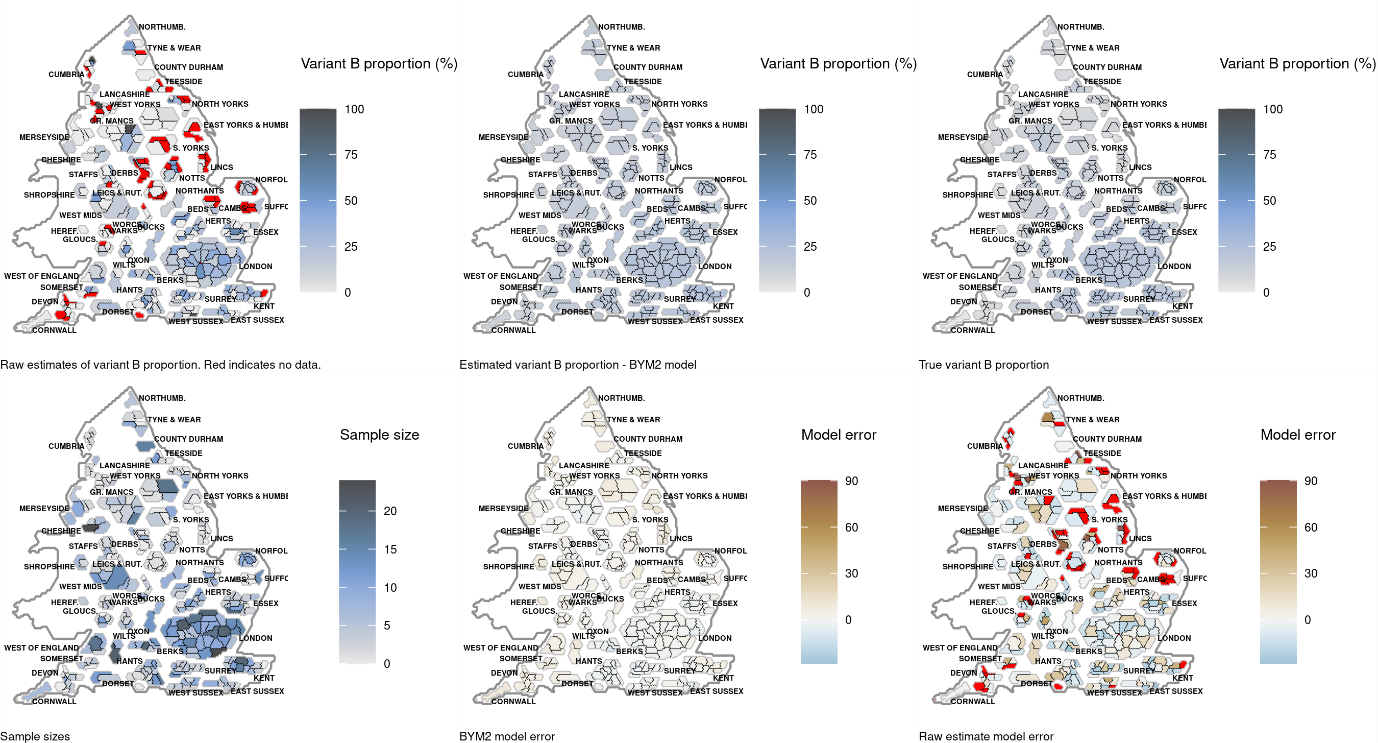
**

Fig A4. Comparisons of the estimates from the Naive estimator, and the BYM2


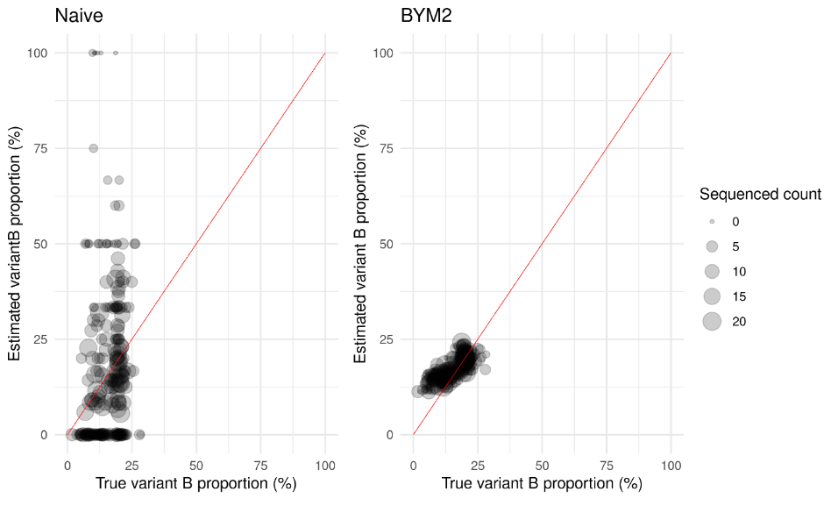


Fig A5. Plots of the true value against the fitted value for the Naive estimator and BYM2


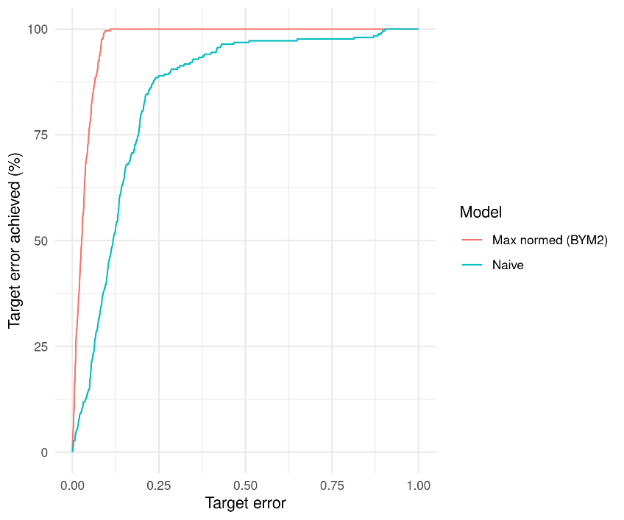


Fig A6. ROC curve of L1 error for the Naive estimate and BYM2

**Results – Scenario 1.2:**

**
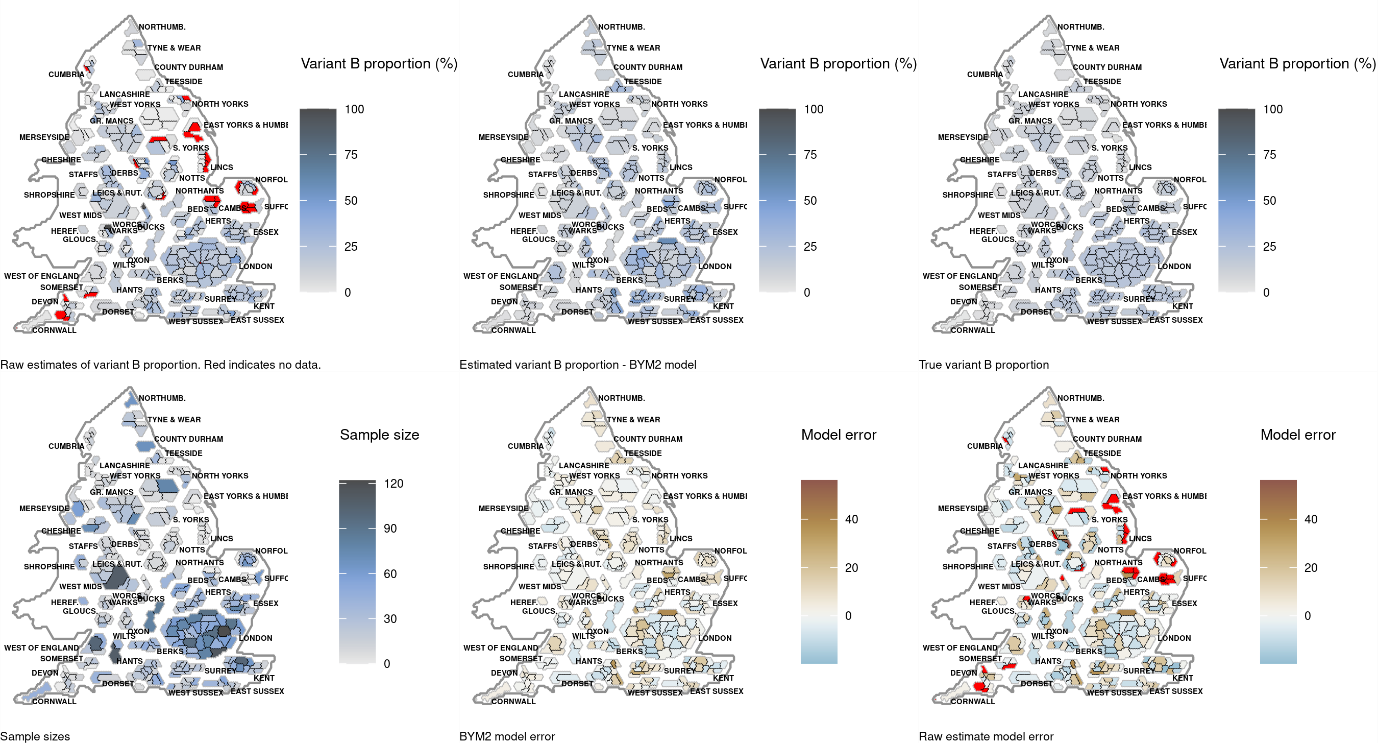
**

Fig A7. Comparisons of the estimates from the Naive estimator, and the BYM2


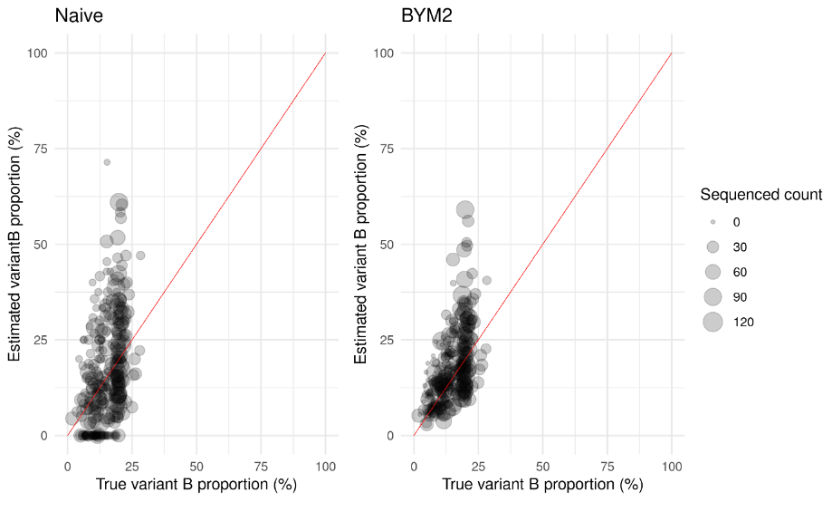


Fig A8. Plots of the true value against the fitted value for the Naive estimator and BYM2


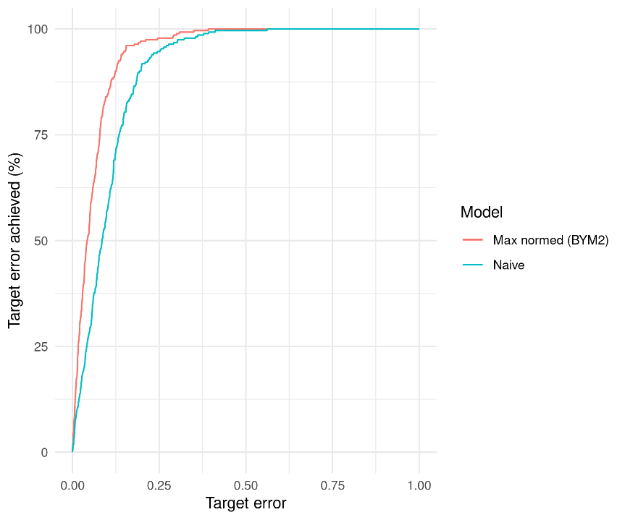


Fig A9. ROC curve of L1 error for the Naive estimate and BYM2

**Results – Scenario 1.3:**

**
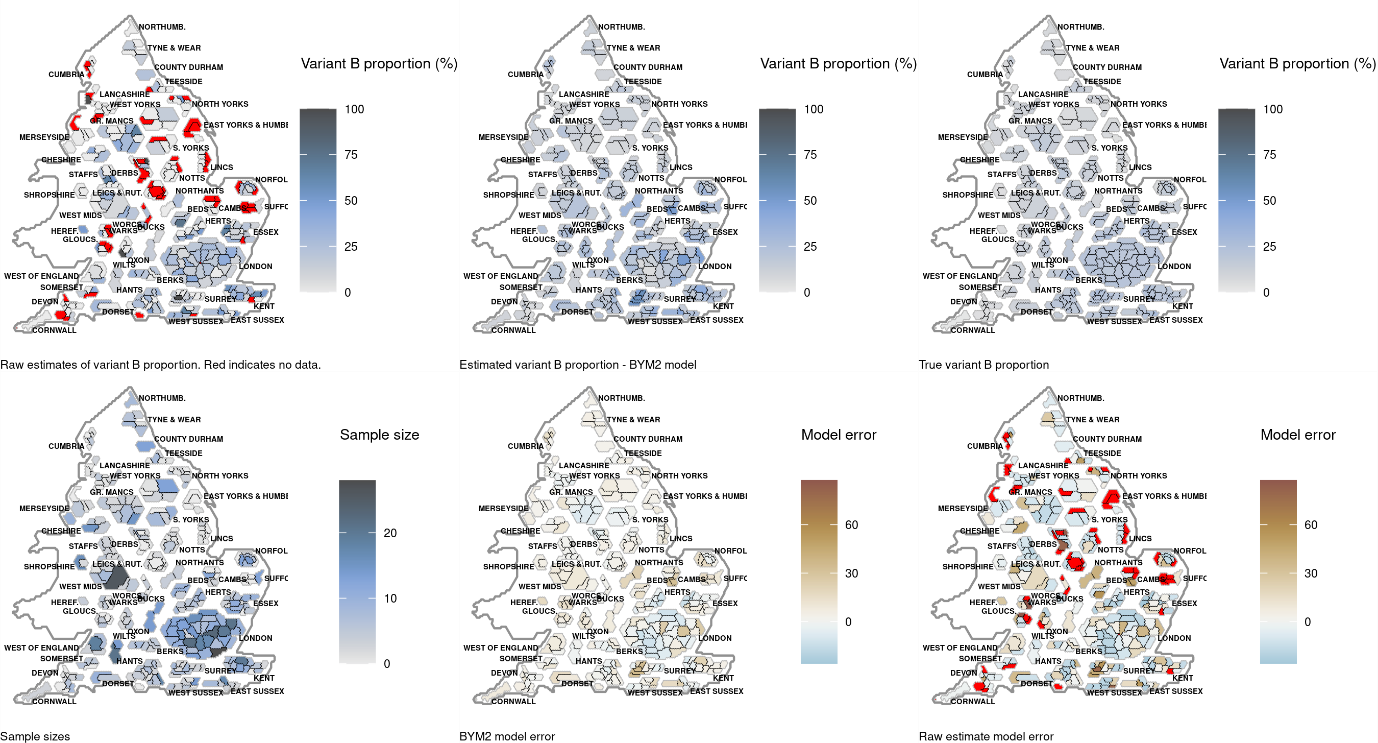
**

Fig A10. Comparisons of the estimates from the Naive estimator, and the BYM2


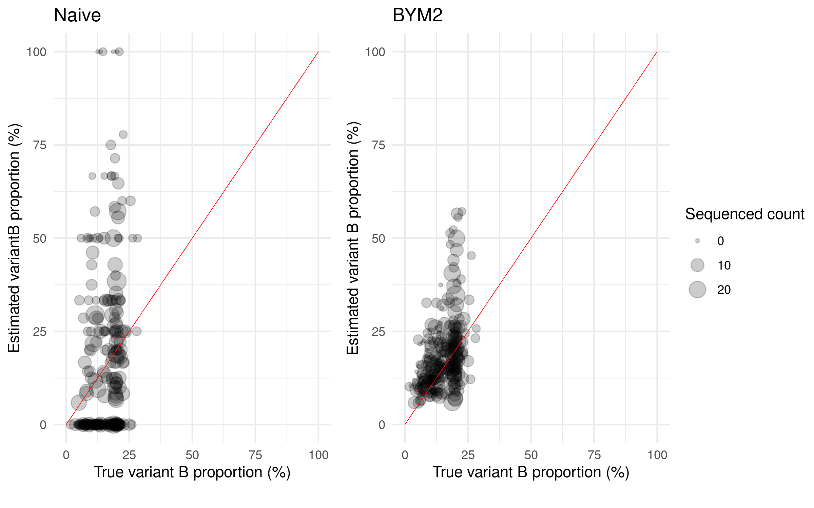


Fig A11. Plots of the true value against the fitted value for the Naive estimator and BYM2


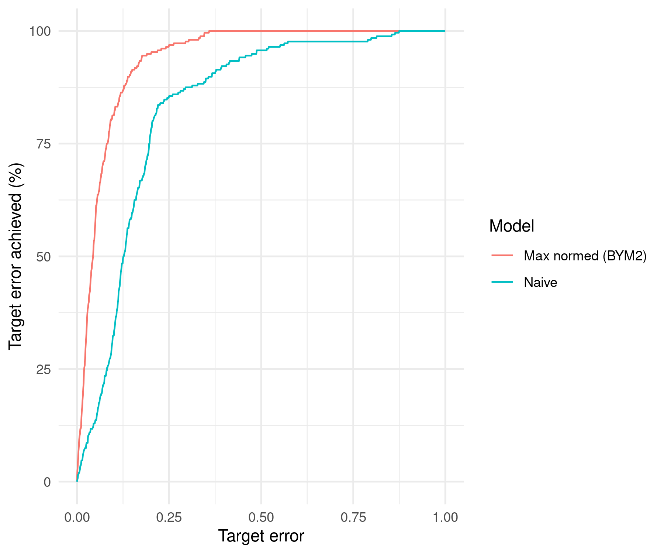


Fig A12. ROC curve of L1 error for the Naive estimate and BYM2

**Results – Scenario 2:**

**
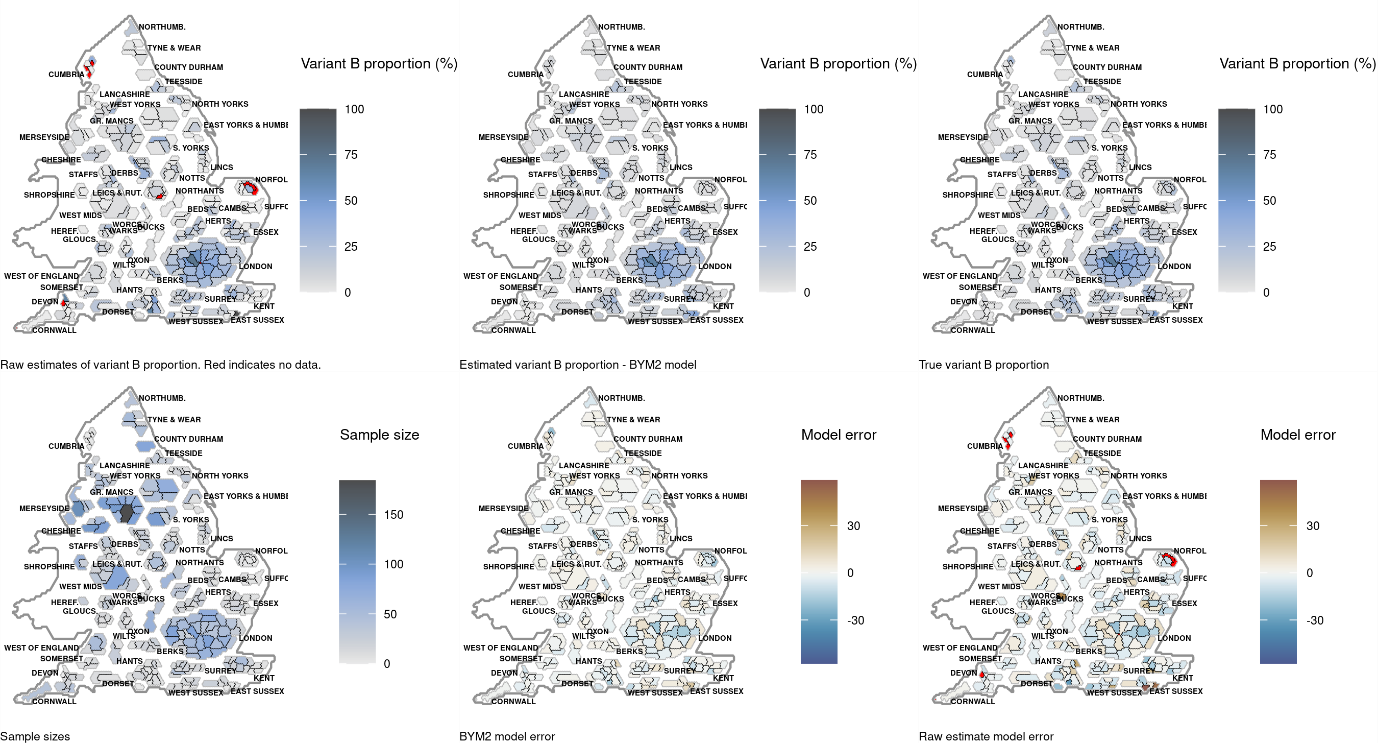
**

Fig A13. Comparisons of the estimates from the Naive estimator, and the BYM2


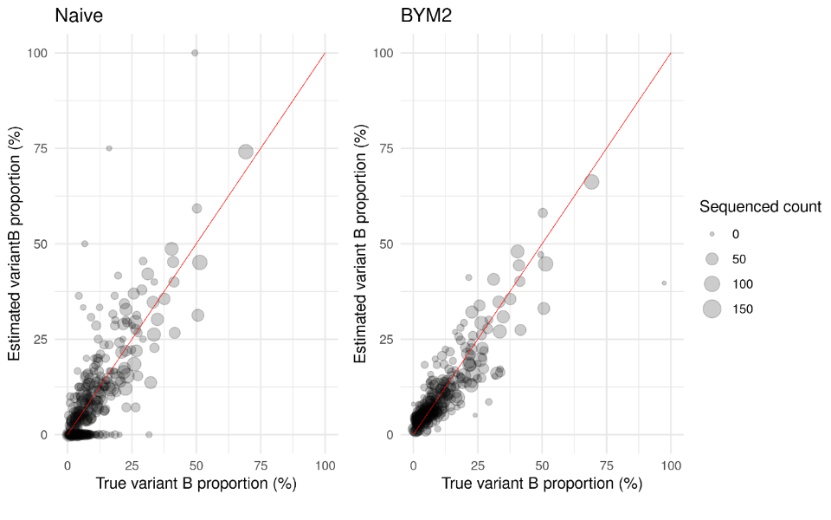


Fig A14. Plots of the true value against the fitted value for the Naive estimator and BYM2


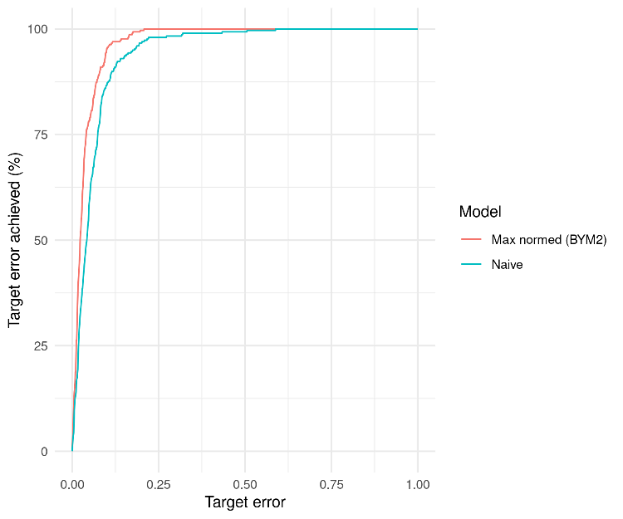


Fig A15. ROC curve of L1 error for the Naive estimate and BYM2

**Results – Scenario 2.1:**

**
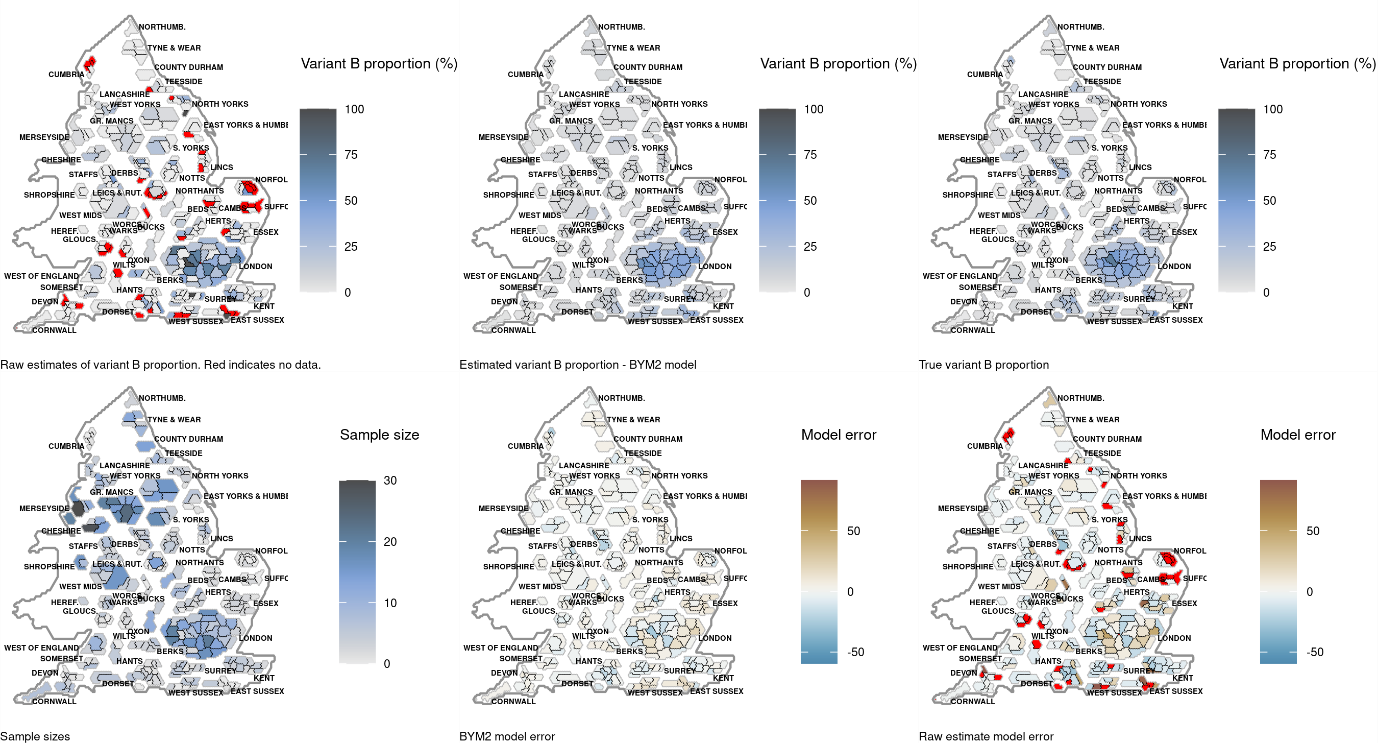
**

Fig A16. Comparisons of the estimates from the Naive estimator, and the BYM2


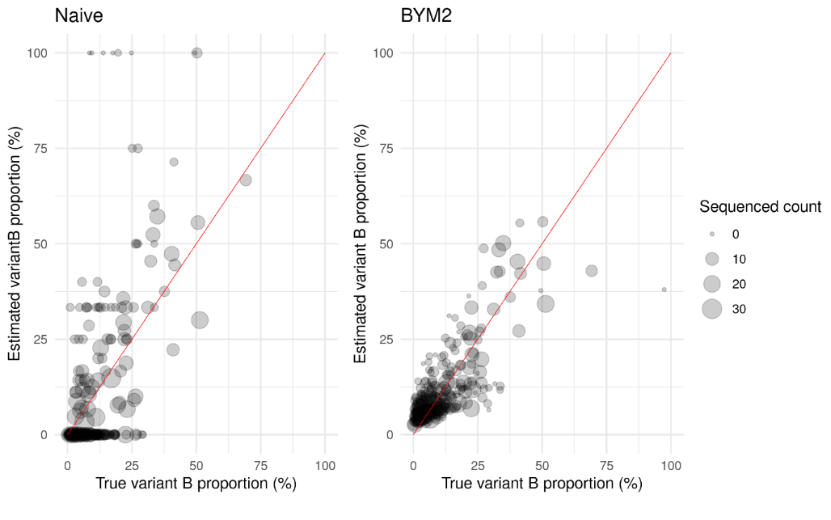


Fig A17. Plots of the true value against the fitted value for the Naive estimator and BYM2


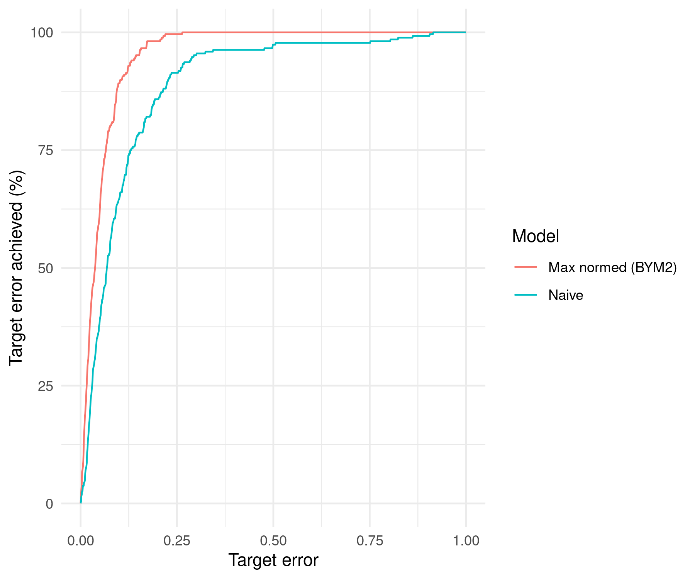


Fig A18. ROC curve of L1 error for the Naive estimate and BYM2

**Results – Scenario 2.2:**

**
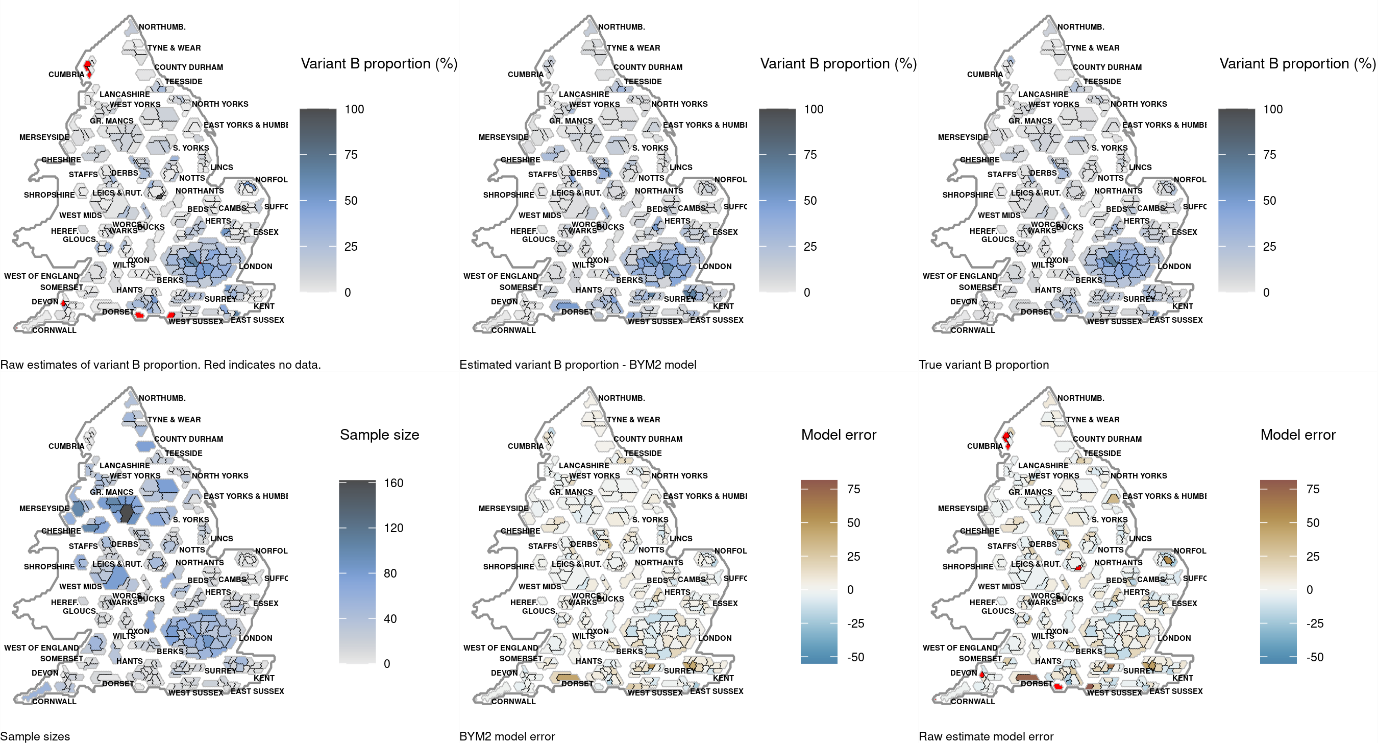
**

Fig A19. Comparisons of the estimates from the Naive estimator, and the BYM2


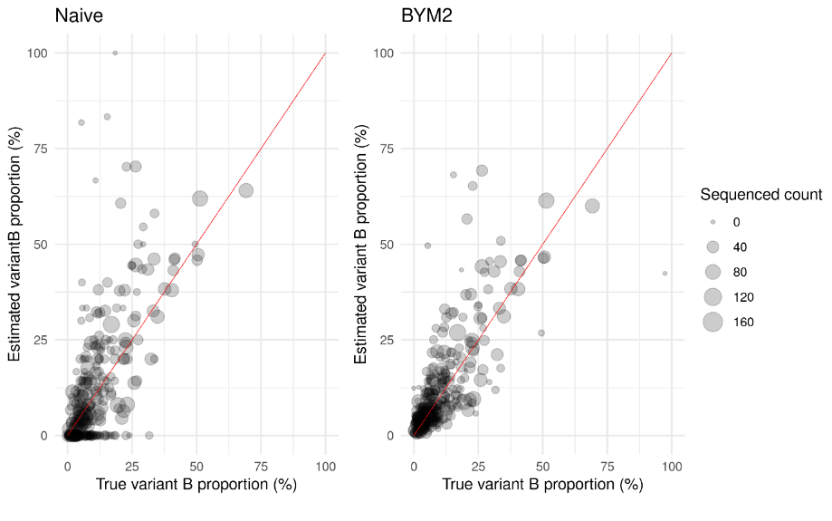


Fig A20. Plots of the true value against the fitted value for the Naive estimator and BYM2


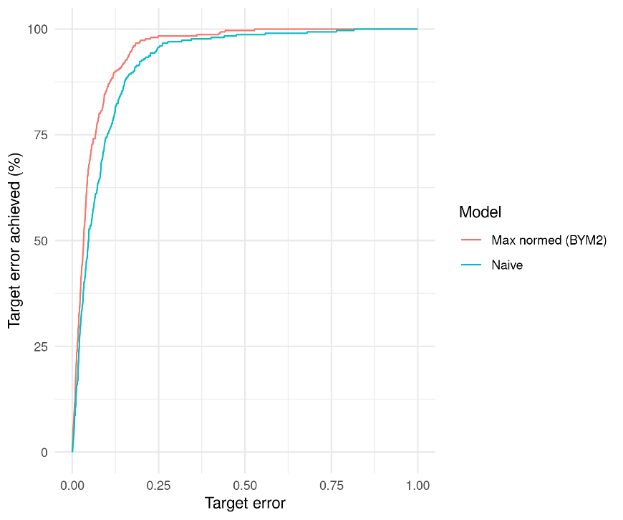


Fig A21. ROC curve of L1 error for the Naive estimate and BYM2

**Results – Scenario 2.3:**

**
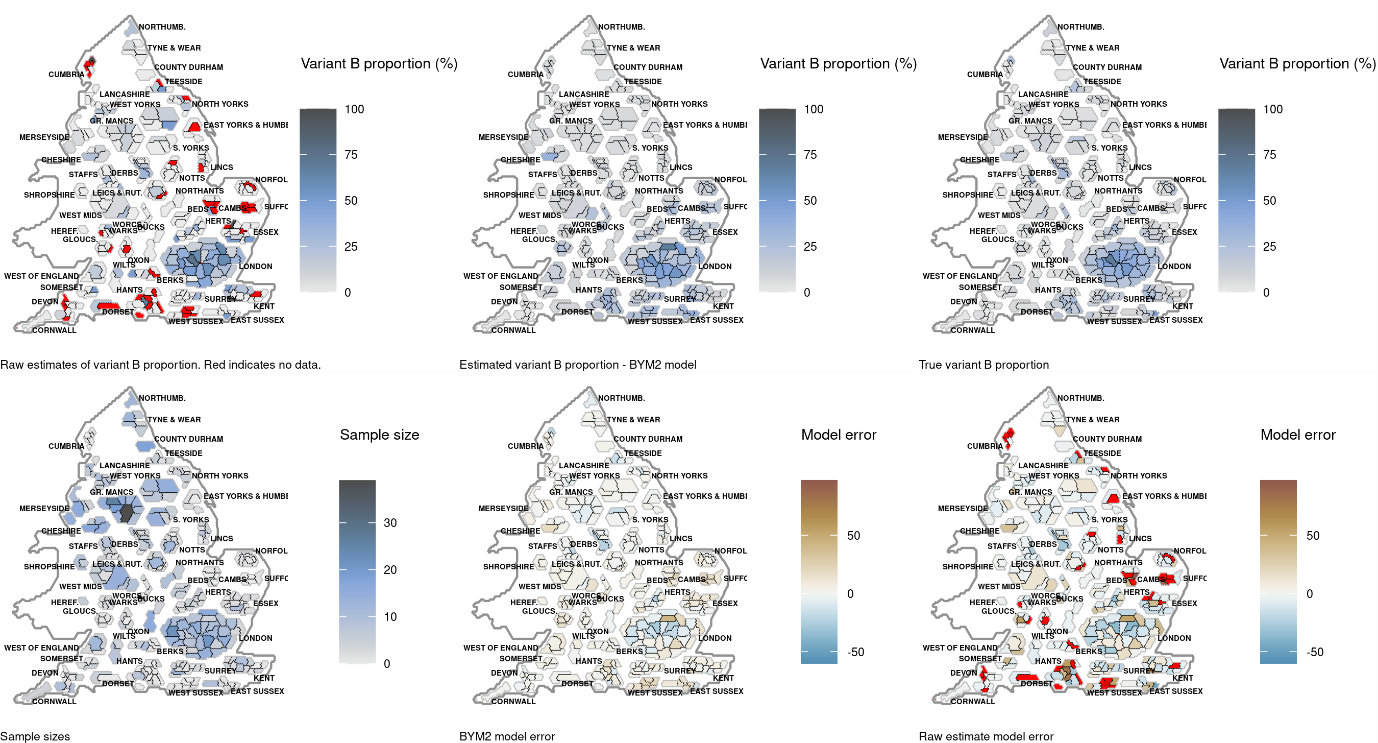
**

Fig A22. Comparisons of the estimates from the Naive estimator, and the BYM2


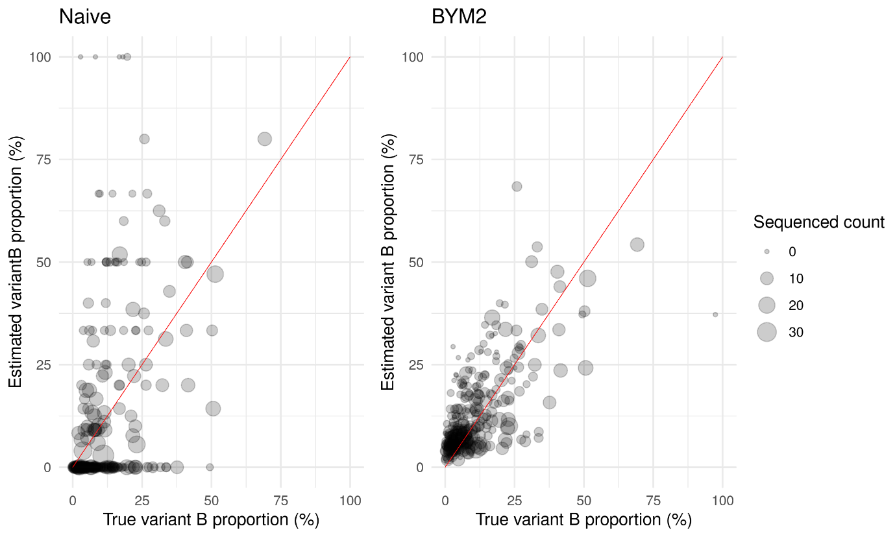


Fig A23. Plots of the true value against the fitted value for the Naive estimator and BYM2


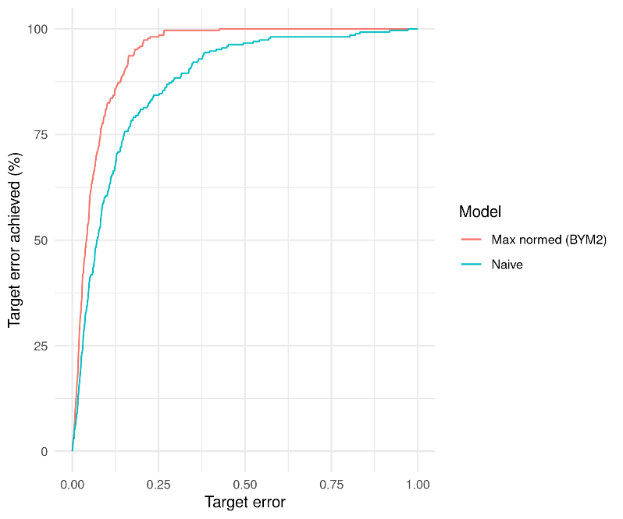


Fig A24. ROC curve of L1 error for the Naive estimate and BYM2

**Results – Scenario 3:**

**
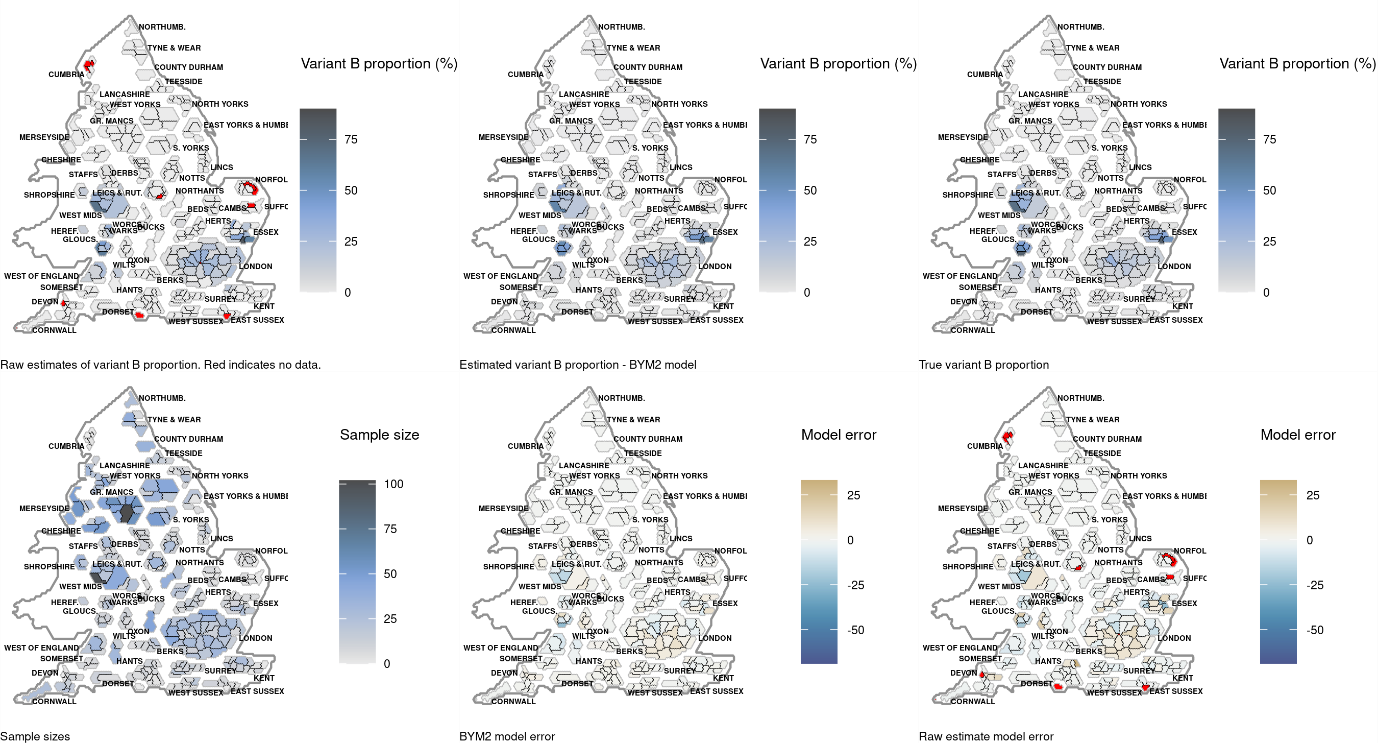
**

Fig A25. Comparisons of the estimates from the Naive estimator, and the BYM2


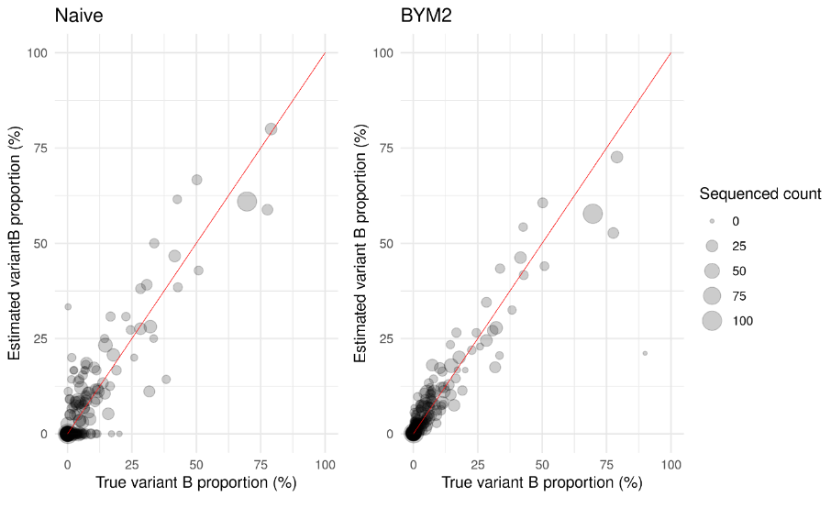


Fig A26. Plots of the true value against the fitted value for the Naive estimator and BYM2


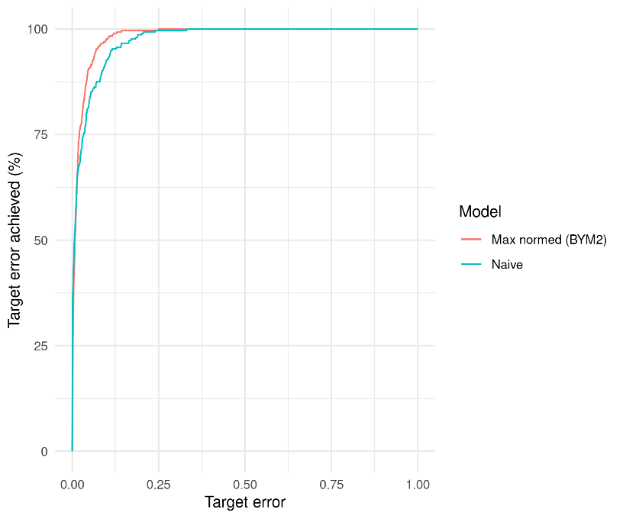


Fig A27. ROC curve of L1 error for the Naive estimate and BYM2

**Results – Scenario 3.1:**

**
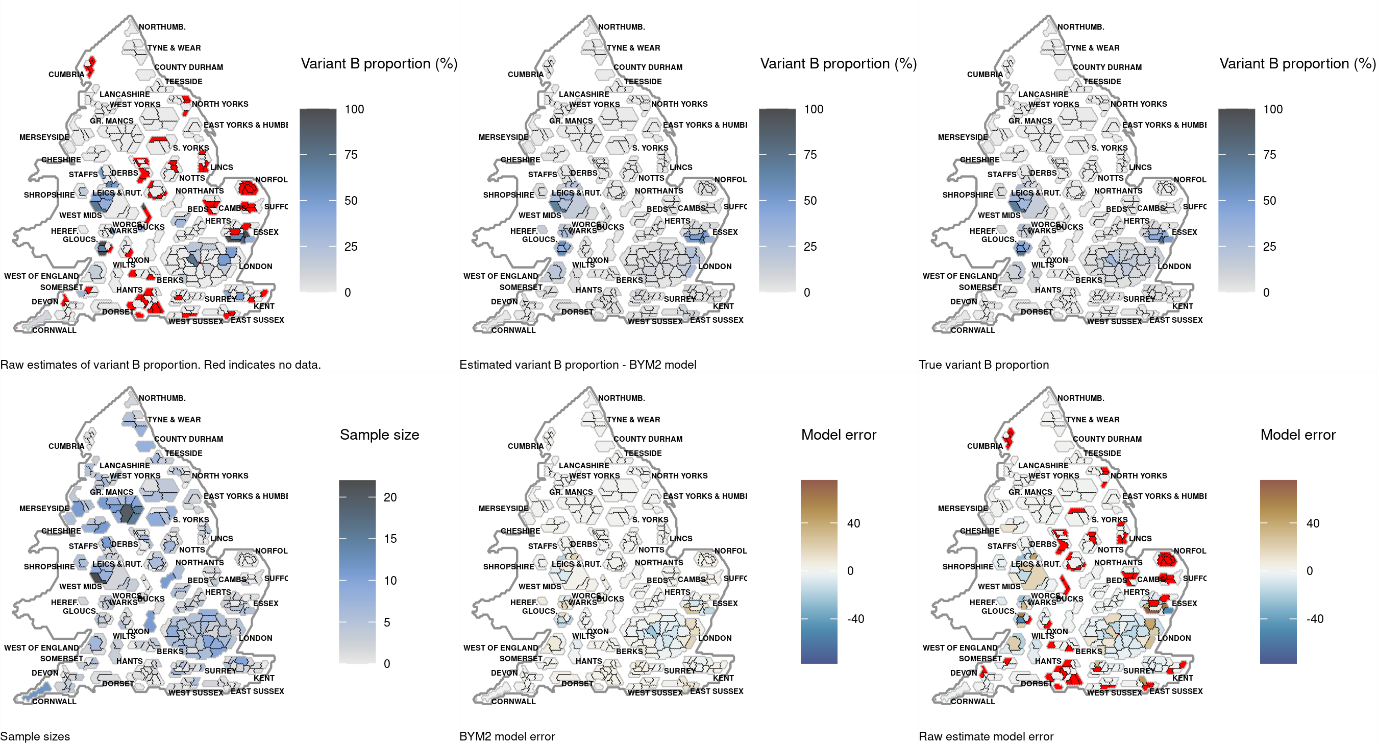
**

Fig A28. Comparisons of the estimates from the Naive estimator, and the BYM2


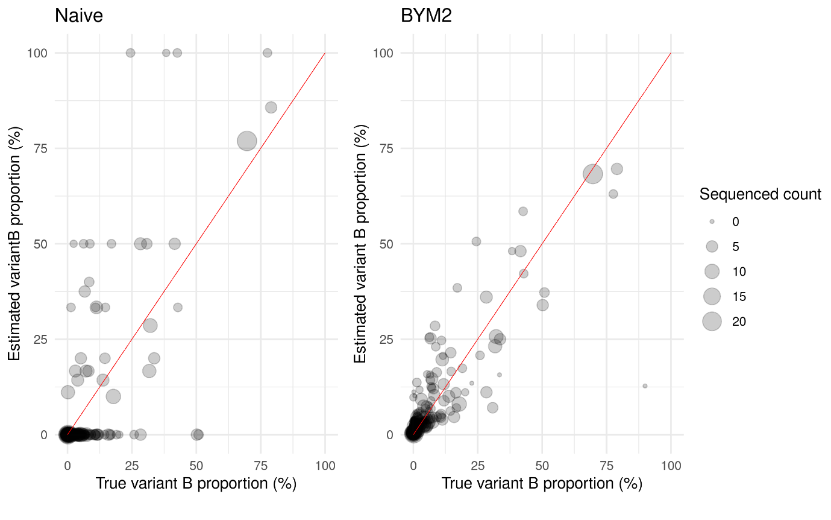


Fig A29. Plots of the true value against the fitted value for the Naive estimator and BYM2


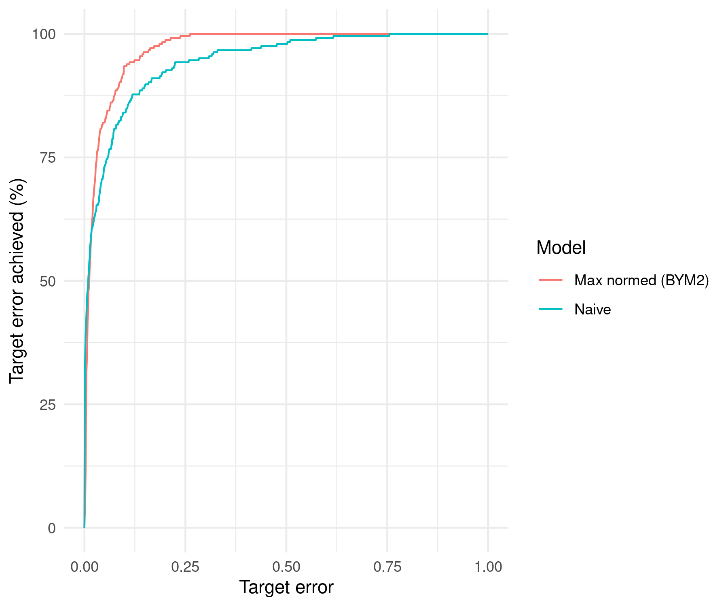


Fig A30. ROC curve of L1 error for the Naive estimate and BYM2

**Results – Scenario 3.2:**

**
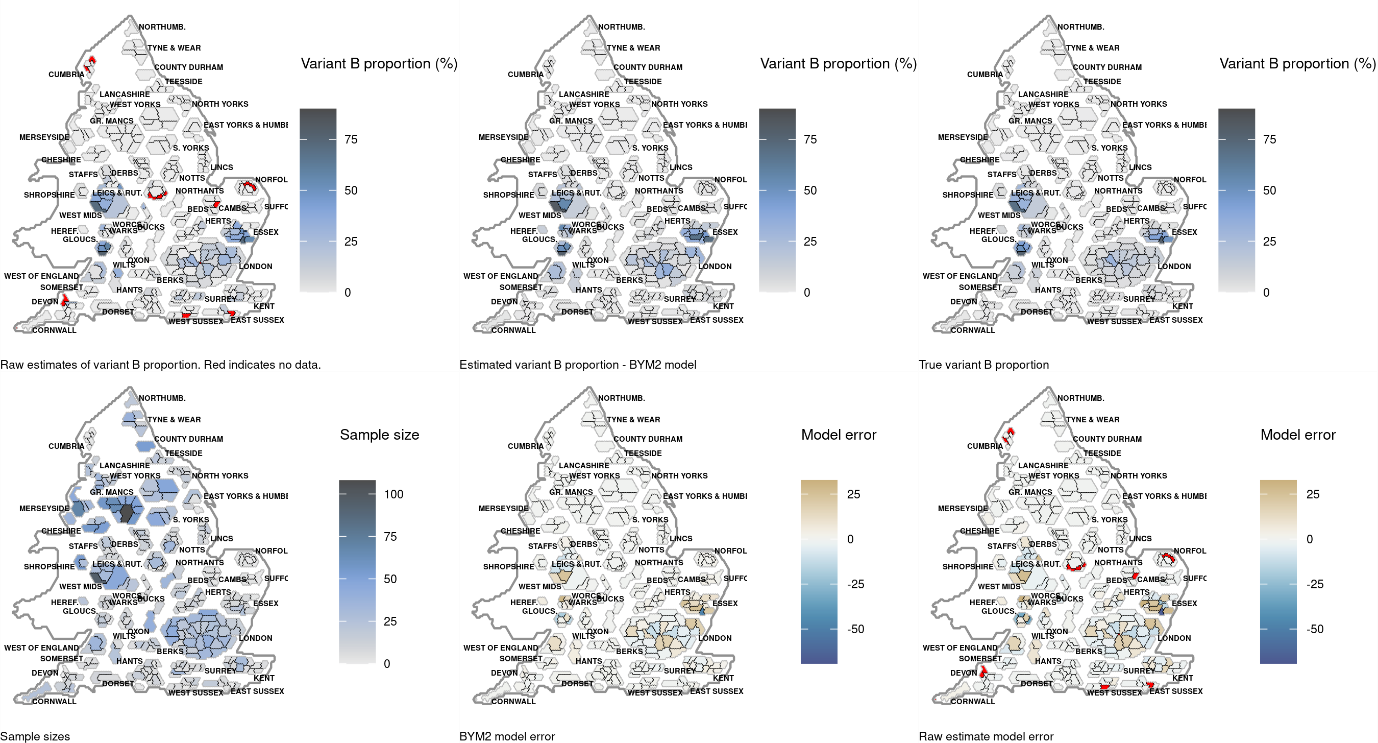
**

Fig A31. Comparisons of the estimates from the Naive estimator, and the BYM2


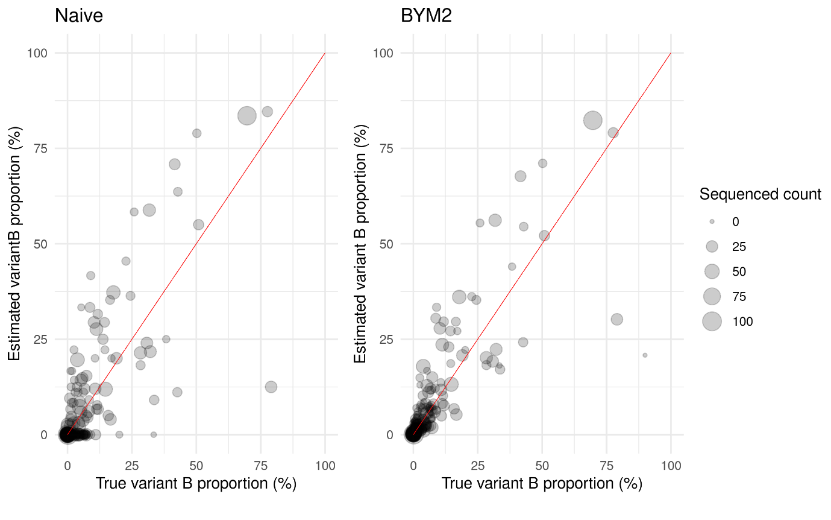


Fig A32. Plots of the true value against the fitted value for the Naive estimator and BYM2


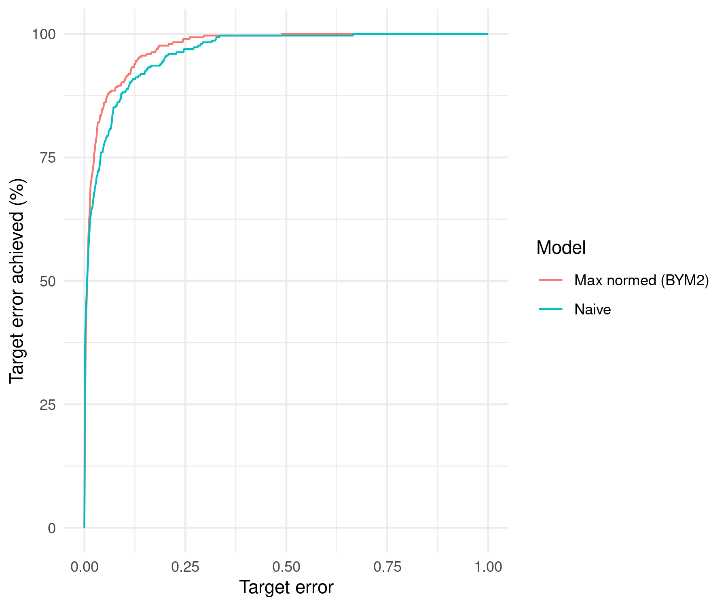


Fig A33. ROC curve of L1 error for the Naive estimate and BYM2

**Results – Scenario 3.3:**

**
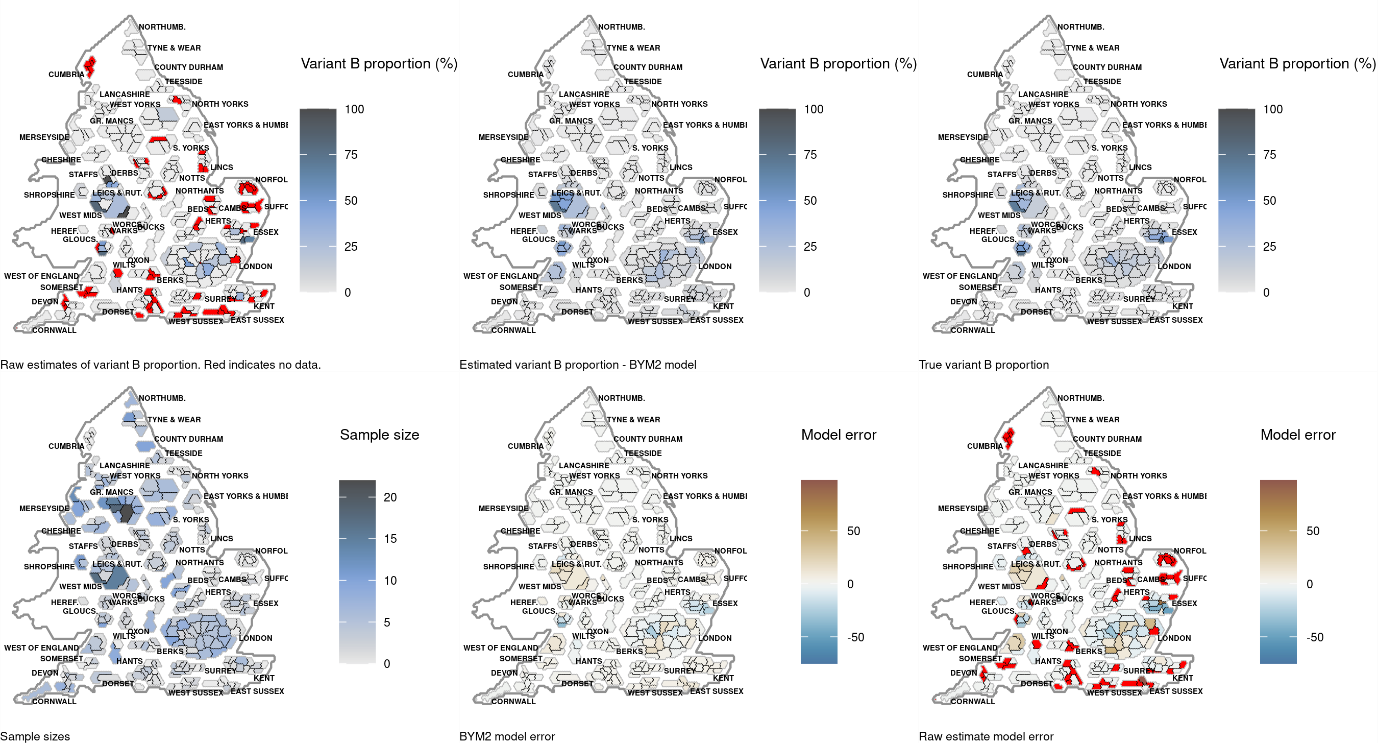
**

Fig A34. Comparisons of the estimates from the Naive estimator, and the BYM2


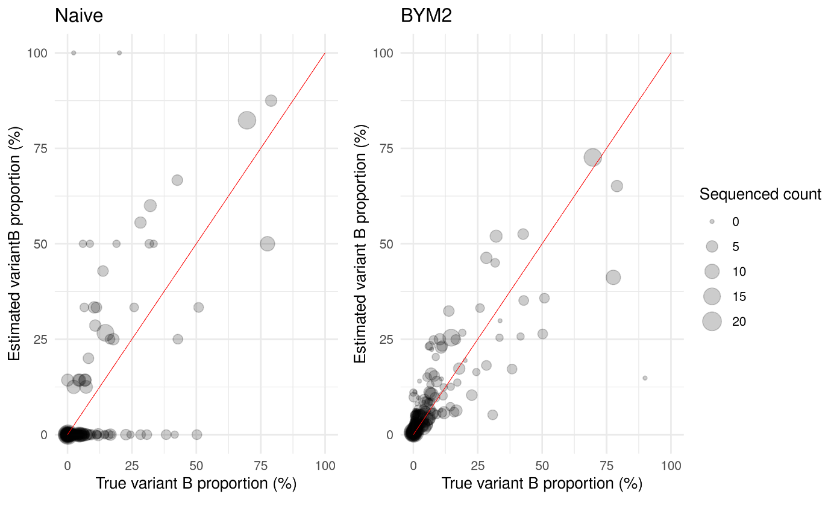


Fig A35. Plots of the true value against the fitted value for the Naive estimator and BYM2


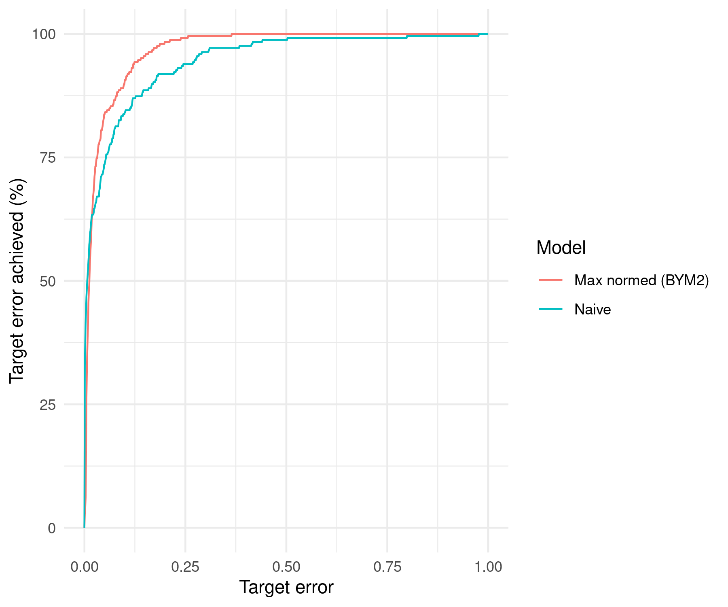


Fig A36. ROC curve of L1 error for the Naive estimate and BYM2


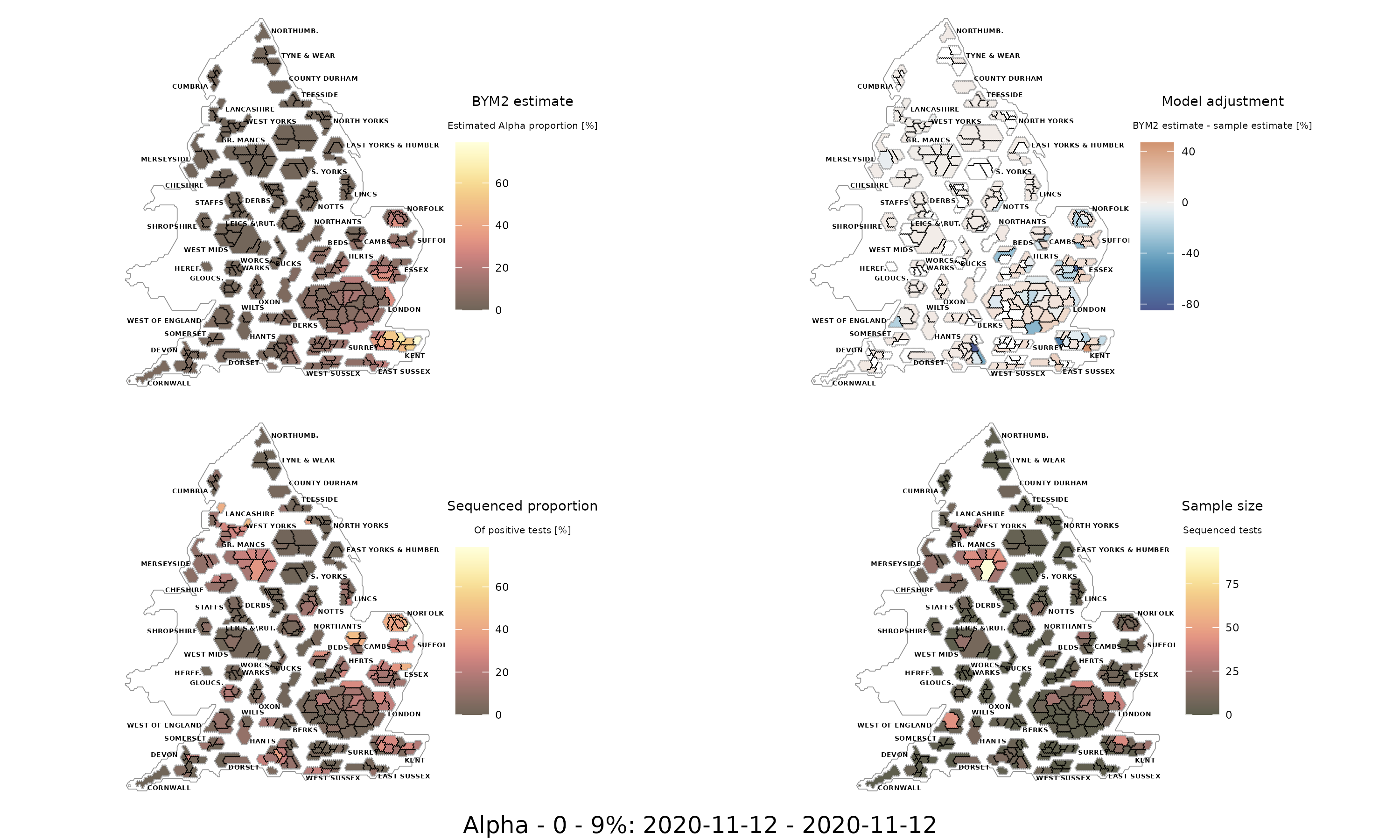


Fig A37. The BYM2 estimated model positivity of the Alpha variant as a proportion of sequenced tests, the model adjustment, the proportion of tests that were sequenced, and the sample size for the time period.


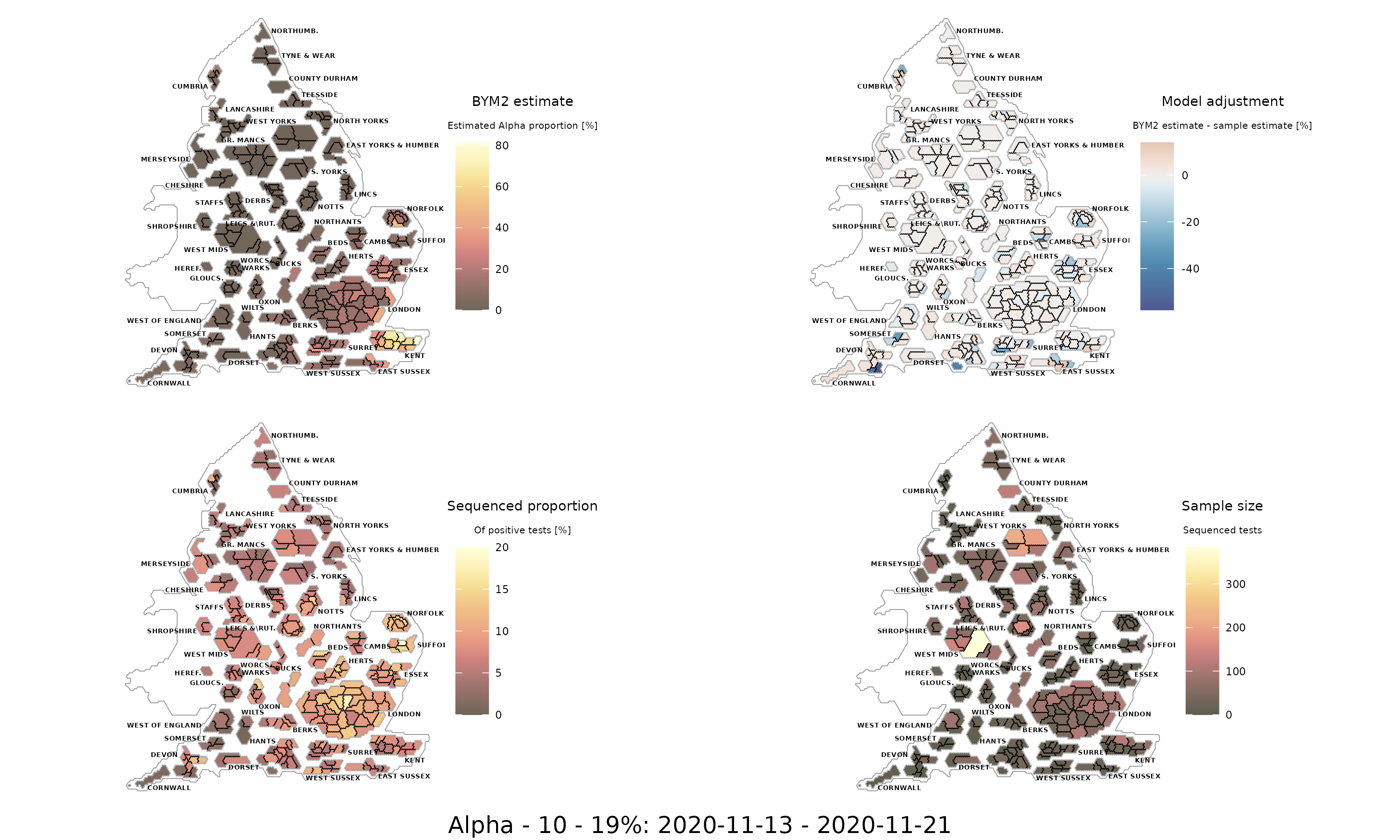


Fig A38. The BYM2 estimated model positivity of the Alpha variant as a proportion of sequenced tests, the model adjustment, the proportion of tests that were sequenced, and the sample size for the time period.


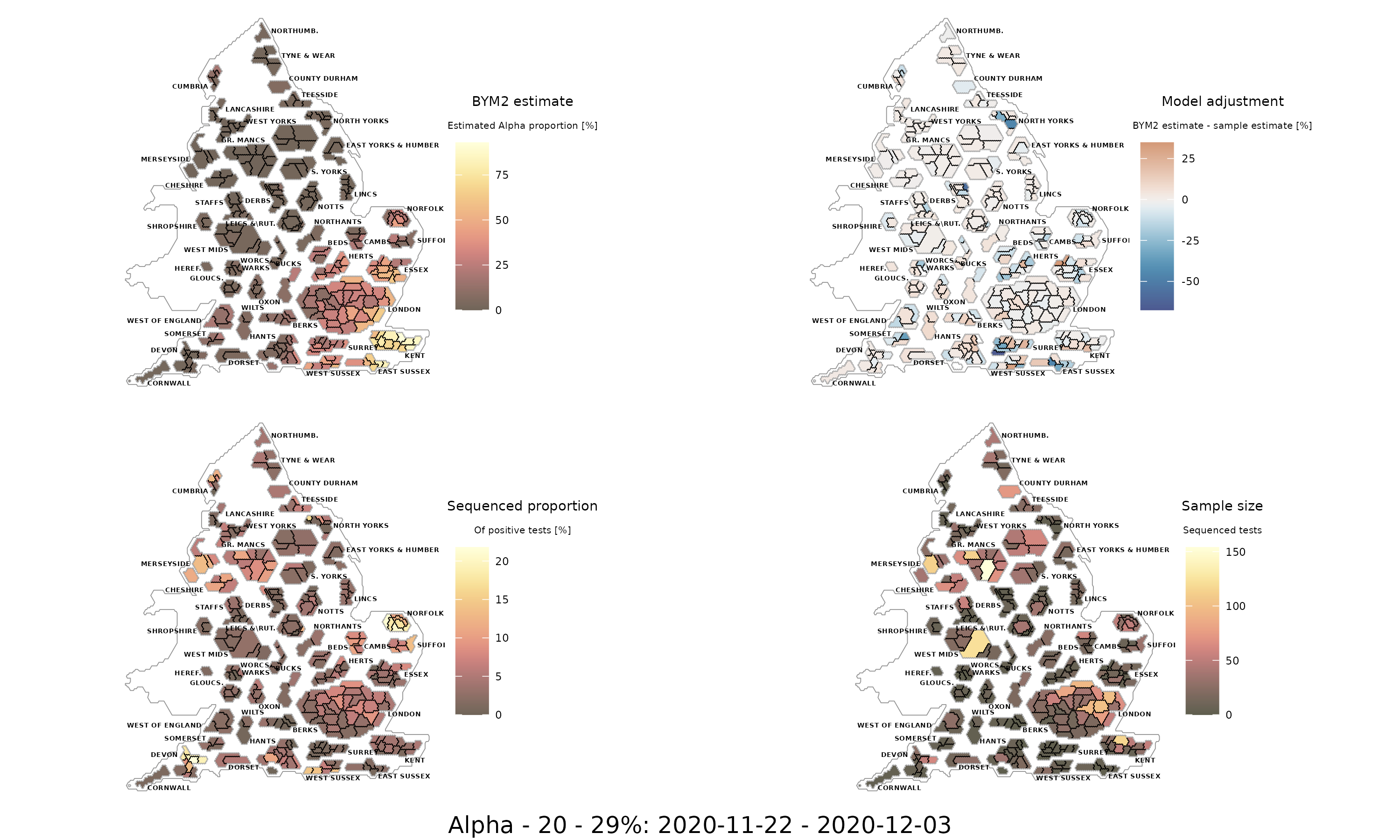


Fig A39. The BYM2 estimated model positivity of the Alpha variant as a proportion of sequenced tests, the model adjustment, the proportion of tests that were sequenced, and the sample size for the time period.


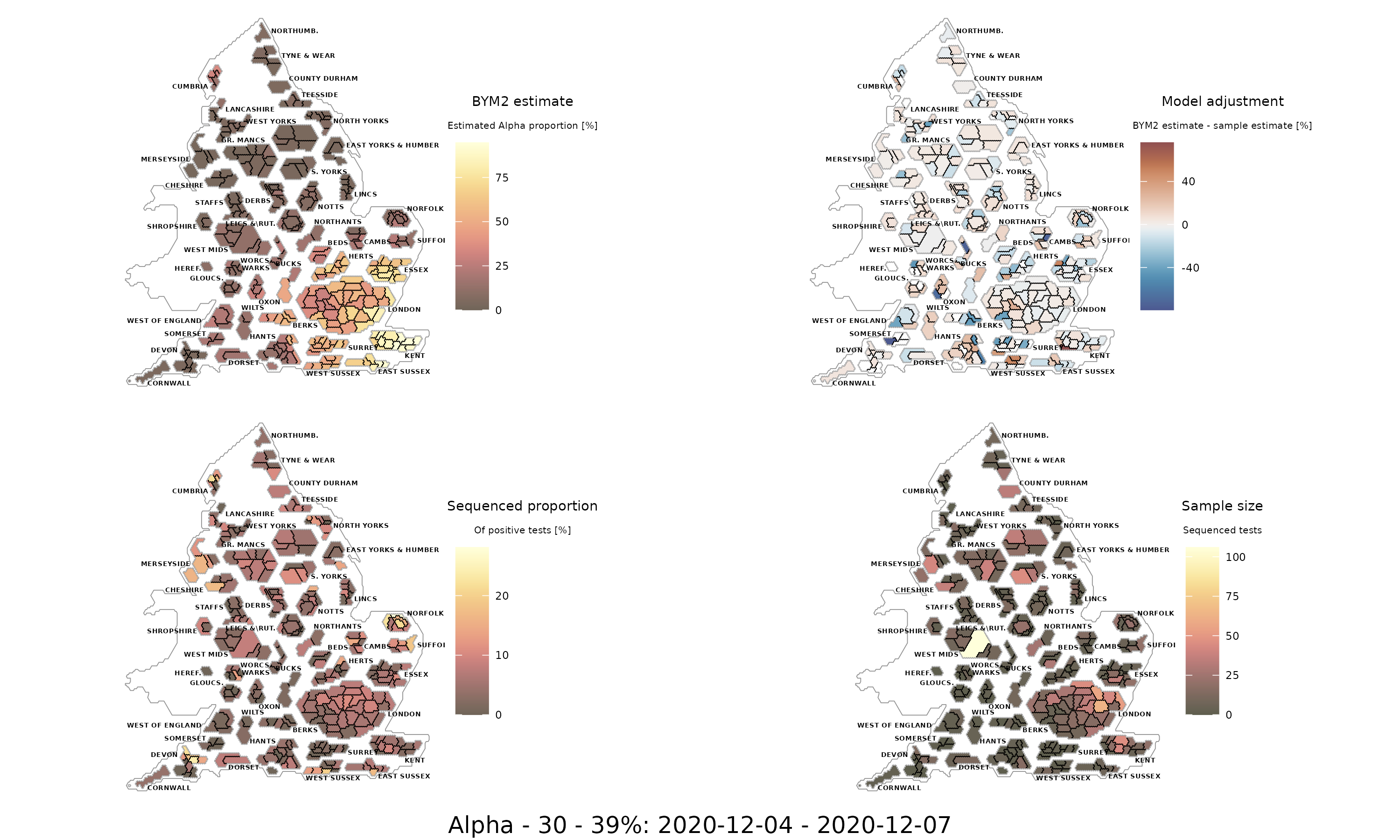


Fig A40. The BYM2 estimated model positivity of the Alpha variant as a proportion of sequenced tests, the model adjustment, the proportion of tests that were sequenced, and the sample size for the time period.


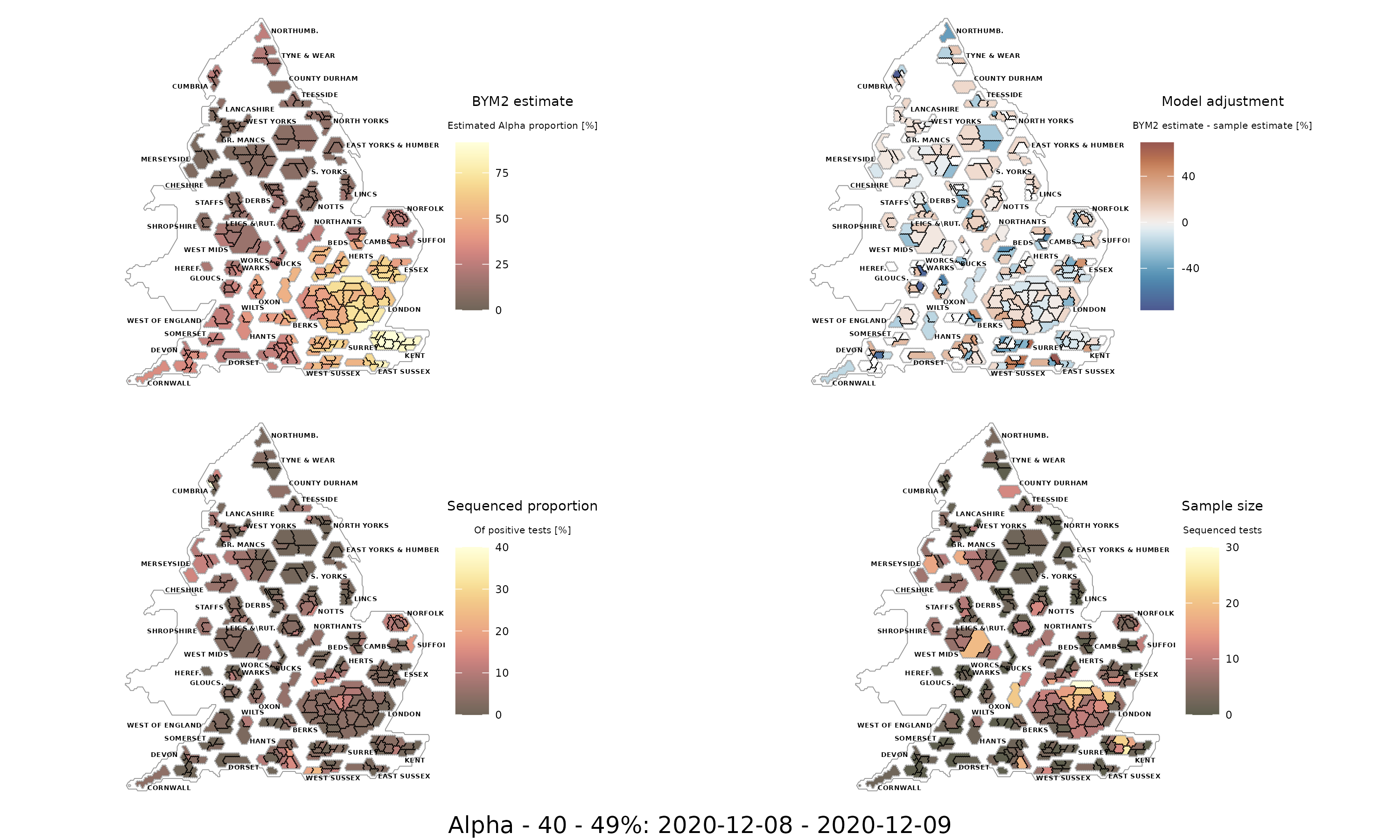


Fig A41. The BYM2 estimated model positivity of the Alpha variant as a proportion of sequenced tests, the model adjustment, the proportion of tests that were sequenced, and the sample size for the time period.


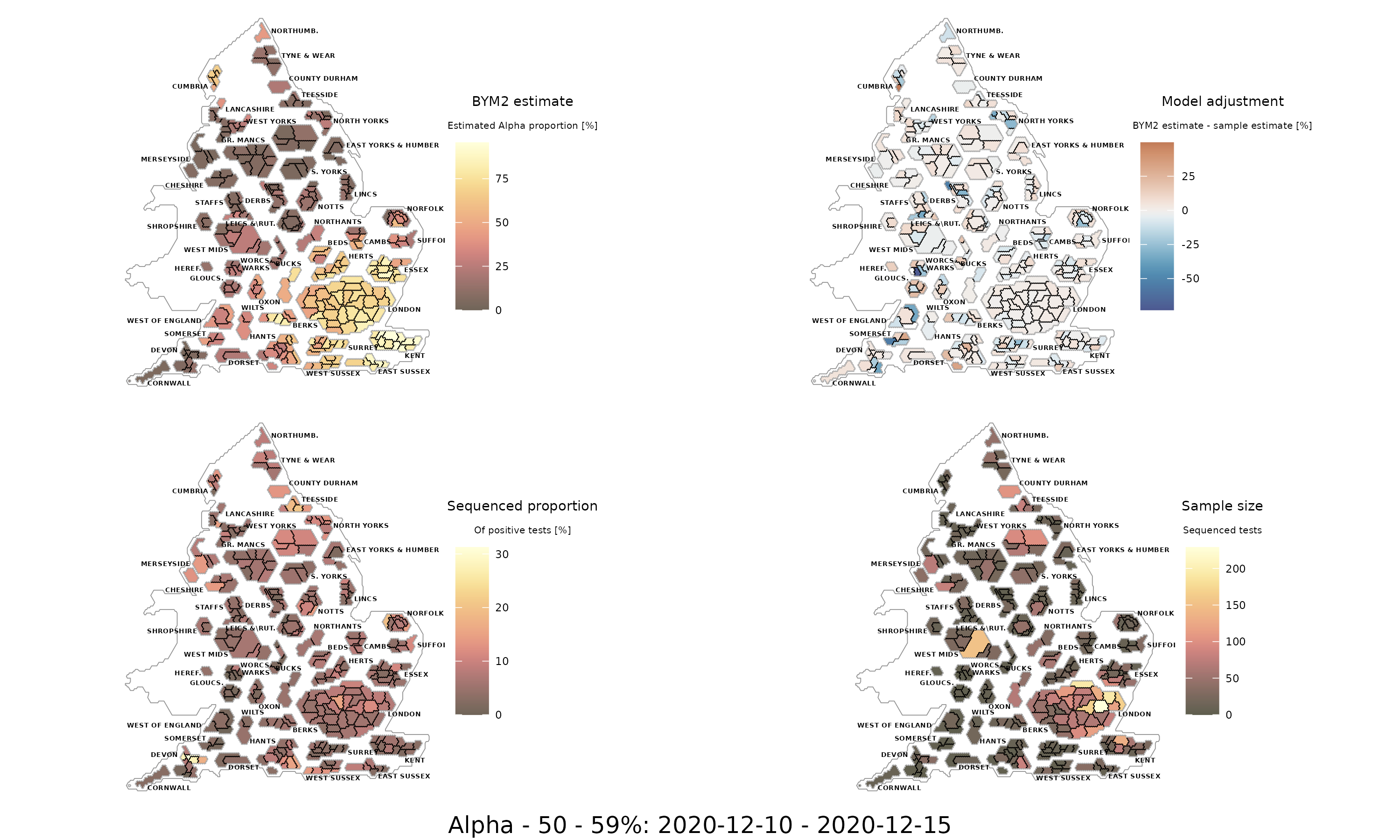


Fig A42. The BYM2 estimated model positivity of the Alpha variant as a proportion of sequenced tests, the model adjustment, the proportion of tests that were sequenced, and the sample size for the time period.


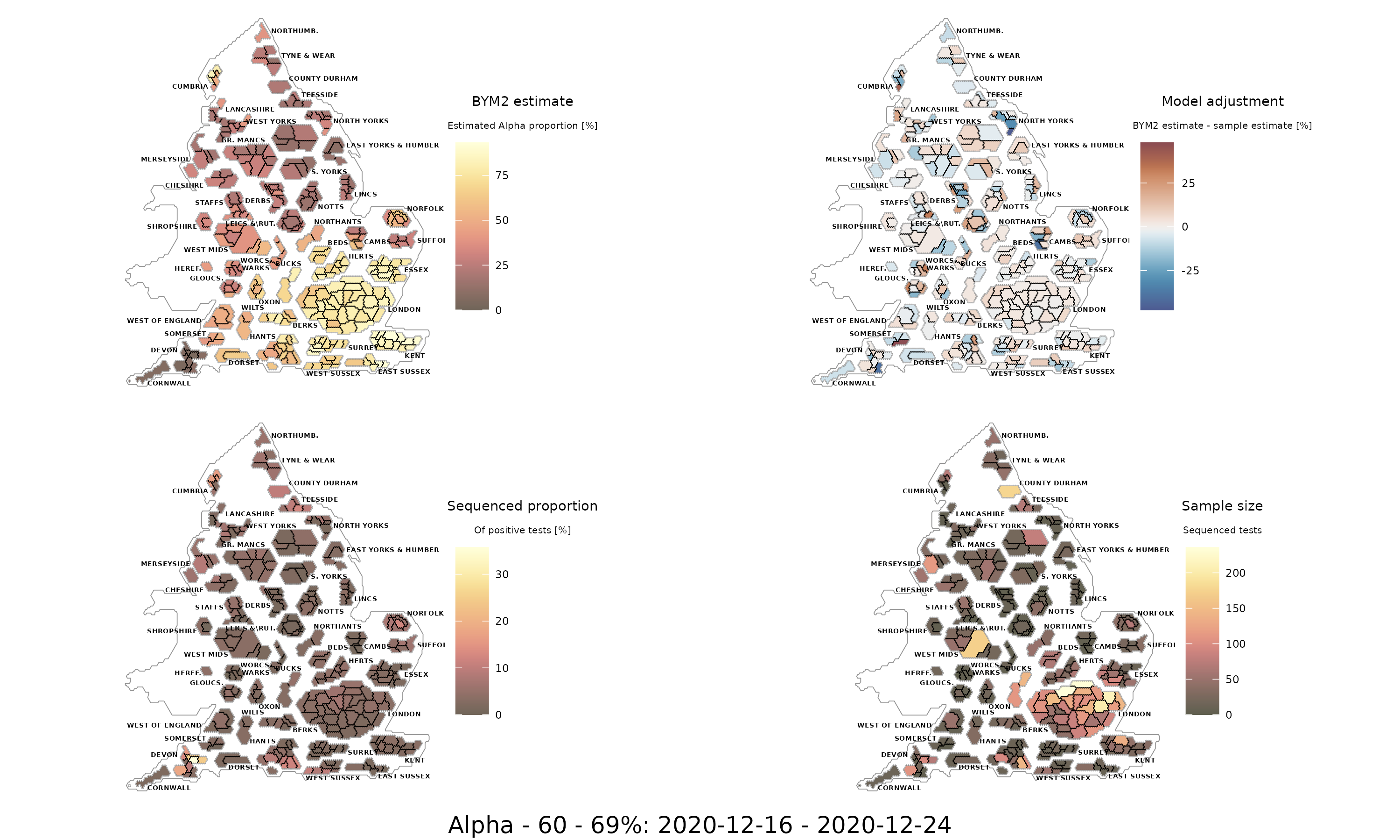


Fig A43. The BYM2 estimated model positivity of the Alpha variant as a proportion of sequenced tests, the model adjustment, the proportion of tests that were sequenced, and the sample size for the time period.


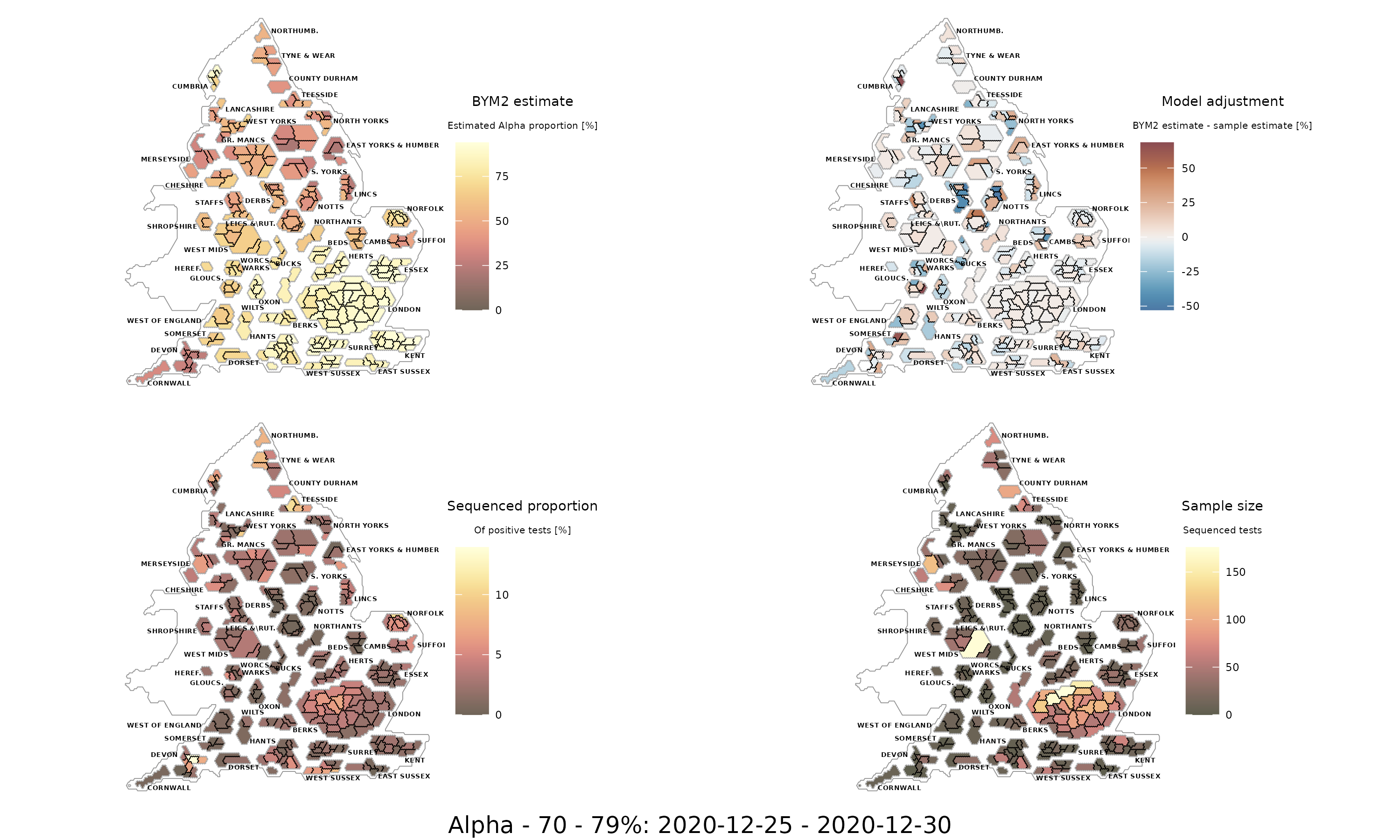


Fig A44. The BYM2 estimated model positivity of the Alpha variant as a proportion of sequenced tests, the model adjustment, the proportion of tests that were sequenced, and the sample size for the time period.


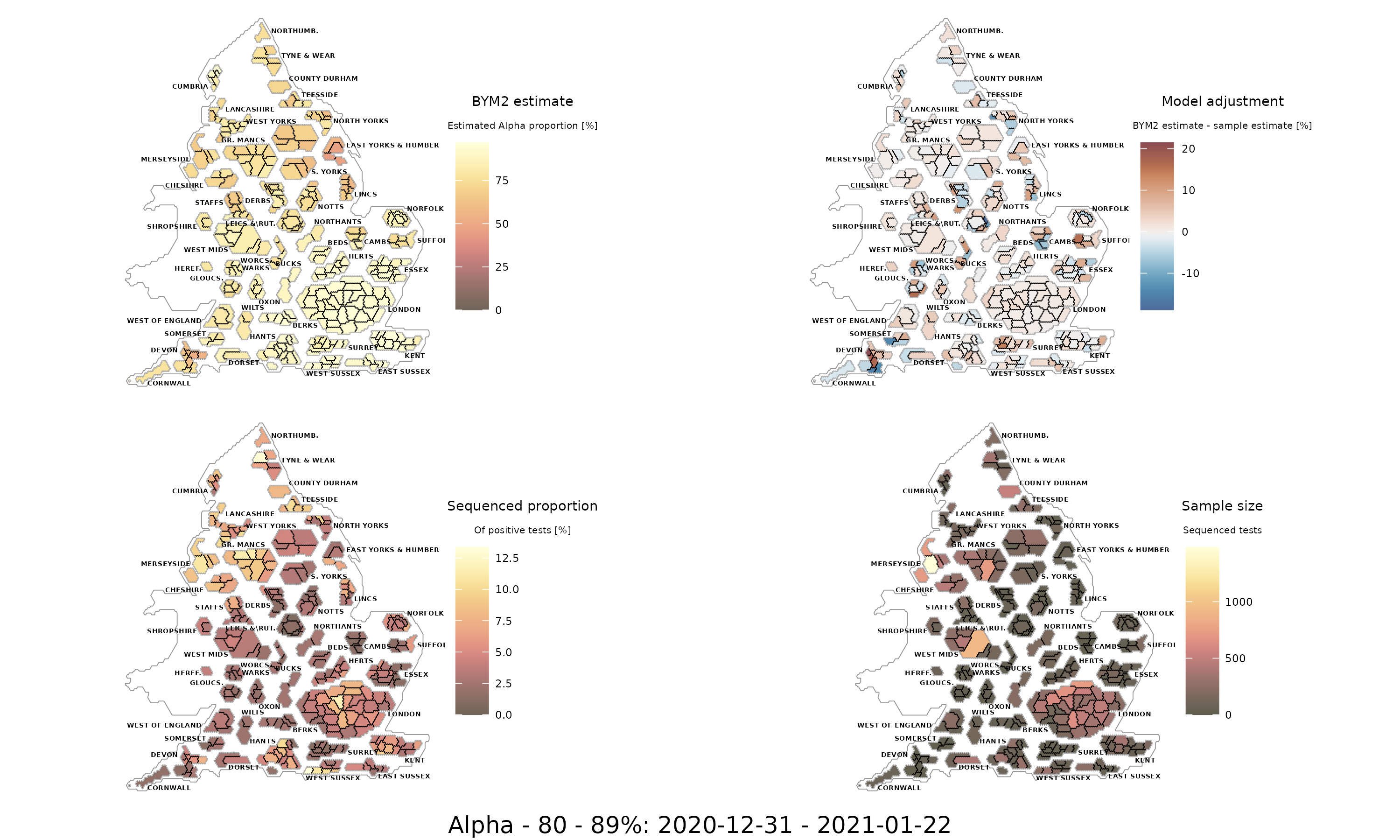


Fig A45. The BYM2 estimated model positivity of the Alpha variant as a proportion of sequenced tests, the model adjustment, the proportion of tests that were sequenced, and the sample size for the time period.


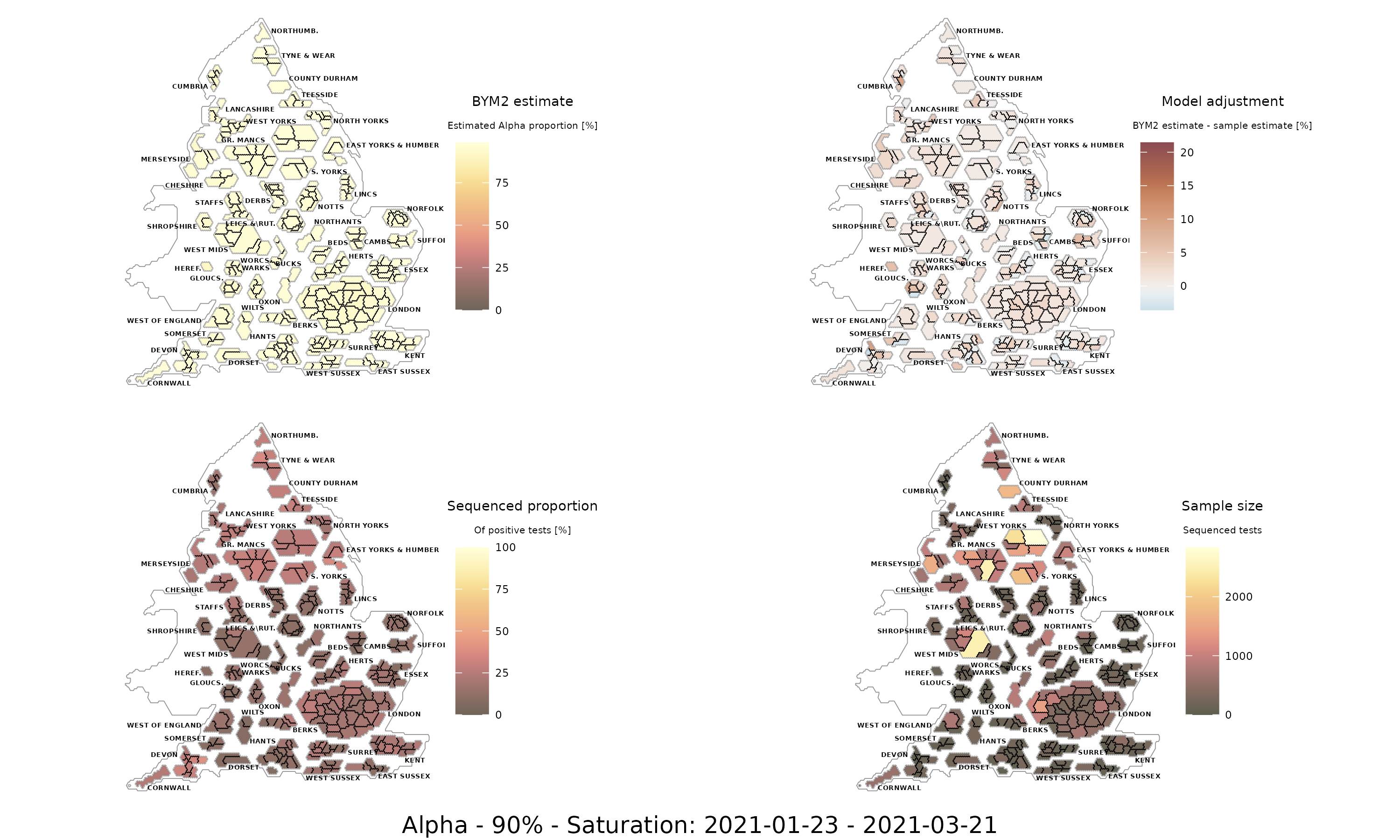


Fig A46. The BYM2 estimated model positivity of the Alpha variant as a proportion of sequenced tests, the model adjustment, the proportion of tests that were sequenced, and the sample size for the time period.


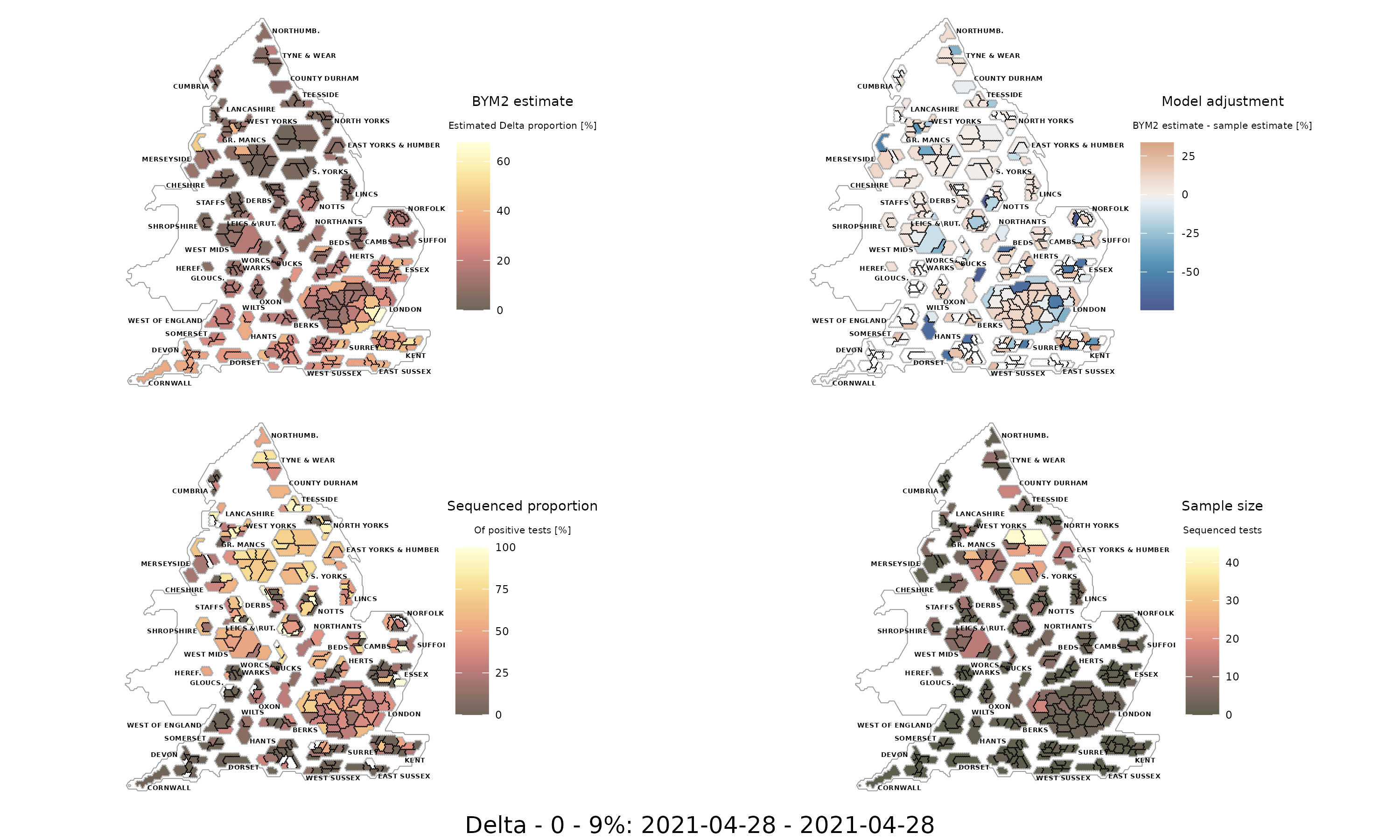


Fig A47. The BYM2 estimated model positivity of the Delta variant as a proportion of sequenced tests, the model adjustment, the proportion of tests that were sequenced, and the sample size for the time period.


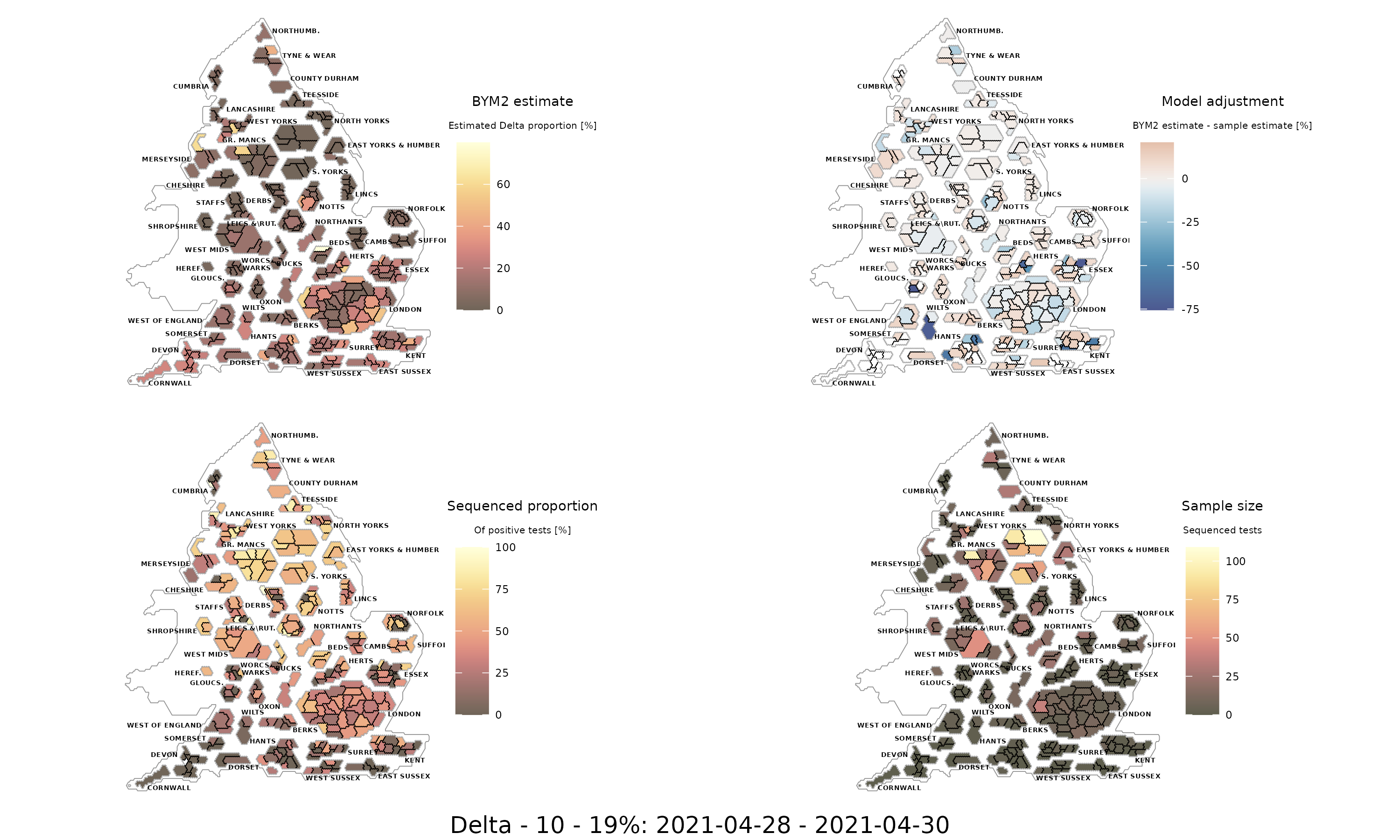


Fig A48. The BYM2 estimated model positivity of the Delta variant as a proportion of sequenced tests, the model adjustment, the proportion of tests that were sequenced, and the sample size for the time period.


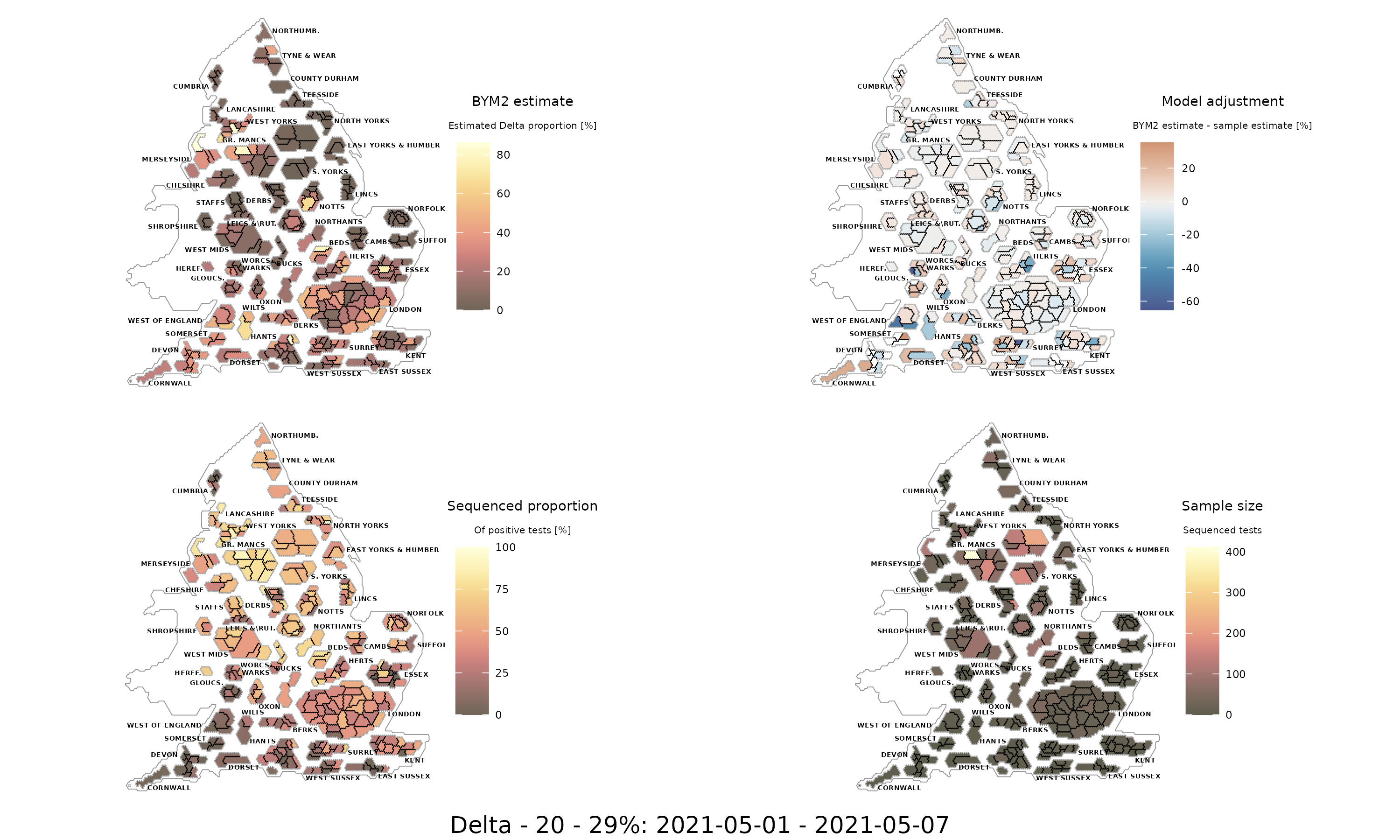


Fig A49. The BYM2 estimated model positivity of the Delta variant as a proportion of sequenced tests, the model adjustment, the proportion of tests that were sequenced, and the sample size for the time period.


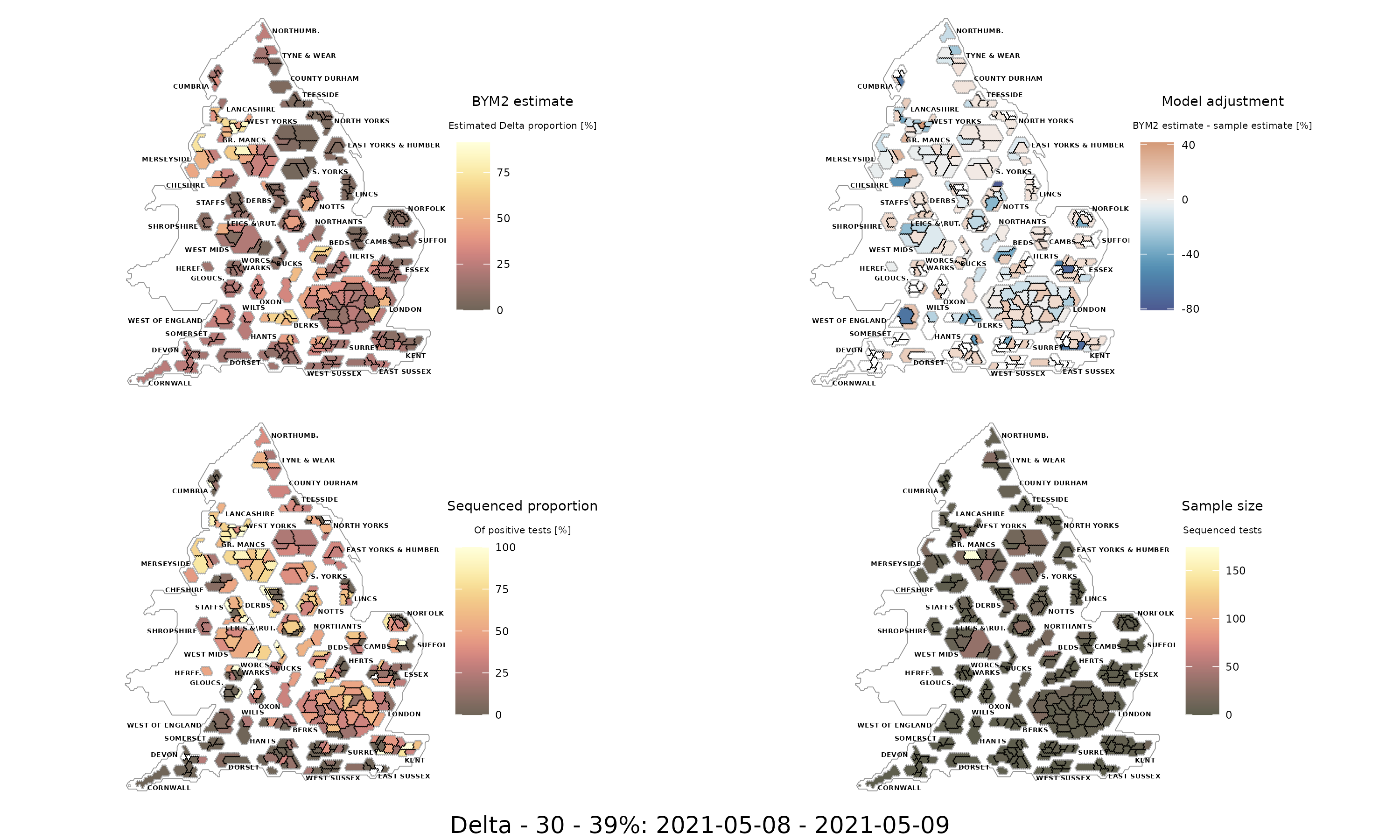


Fig A50. The BYM2 estimated model positivity of the Delta variant as a proportion of sequenced tests, the model adjustment, the proportion of tests that were sequenced, and the sample size for the time period.


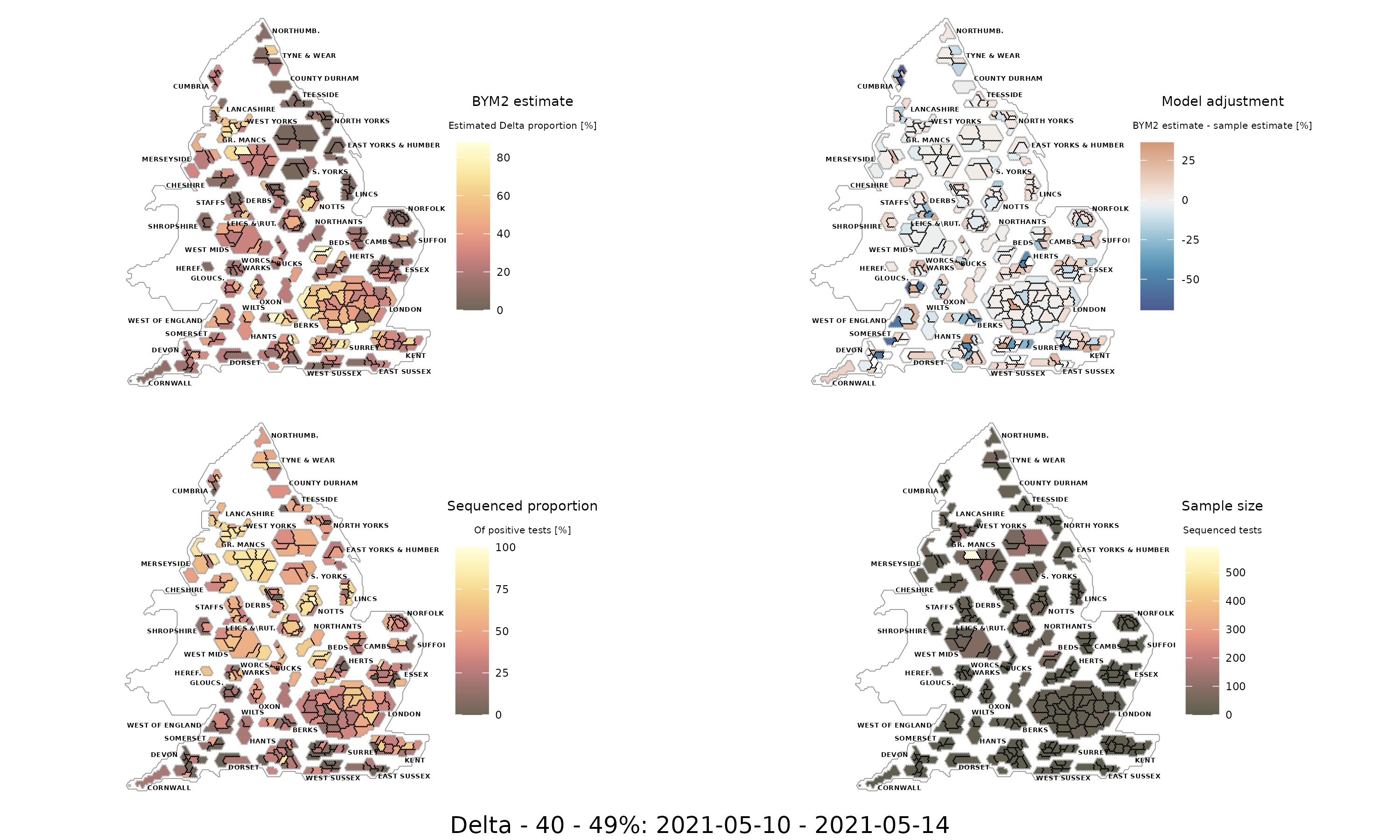


Fig A51. The BYM2 estimated model positivity of the Delta variant as a proportion of sequenced tests, the model adjustment, the proportion of tests that were sequenced, and the sample size for the time period.


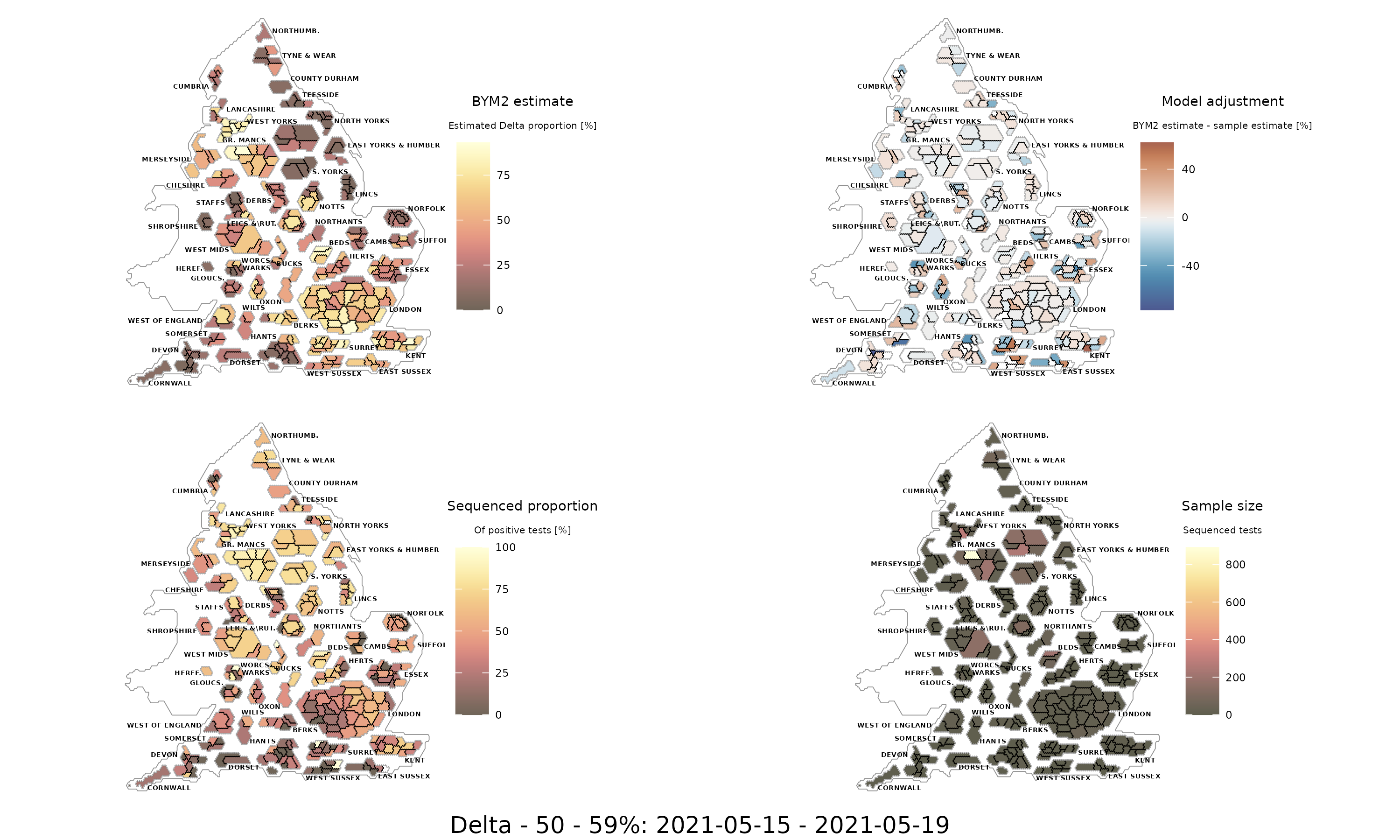


Fig A52. The BYM2 estimated model positivity of the Delta variant as a proportion of sequenced tests, the model adjustment, the proportion of tests that were sequenced, and the sample size for the time period.


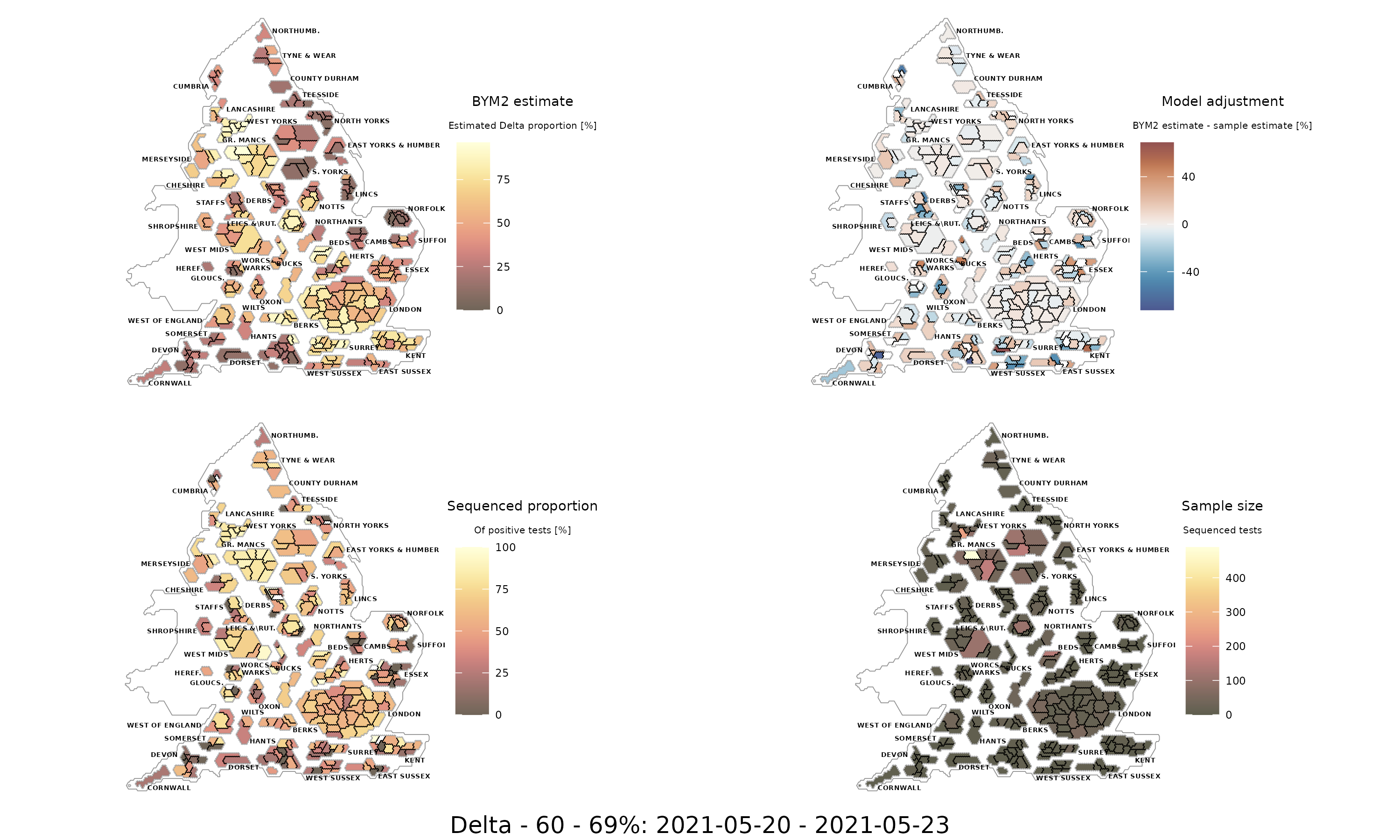


Fig A53. The BYM2 estimated model positivity of the Delta variant as a proportion of sequenced tests, the model adjustment, the proportion of tests that were sequenced, and the sample size for the time period.


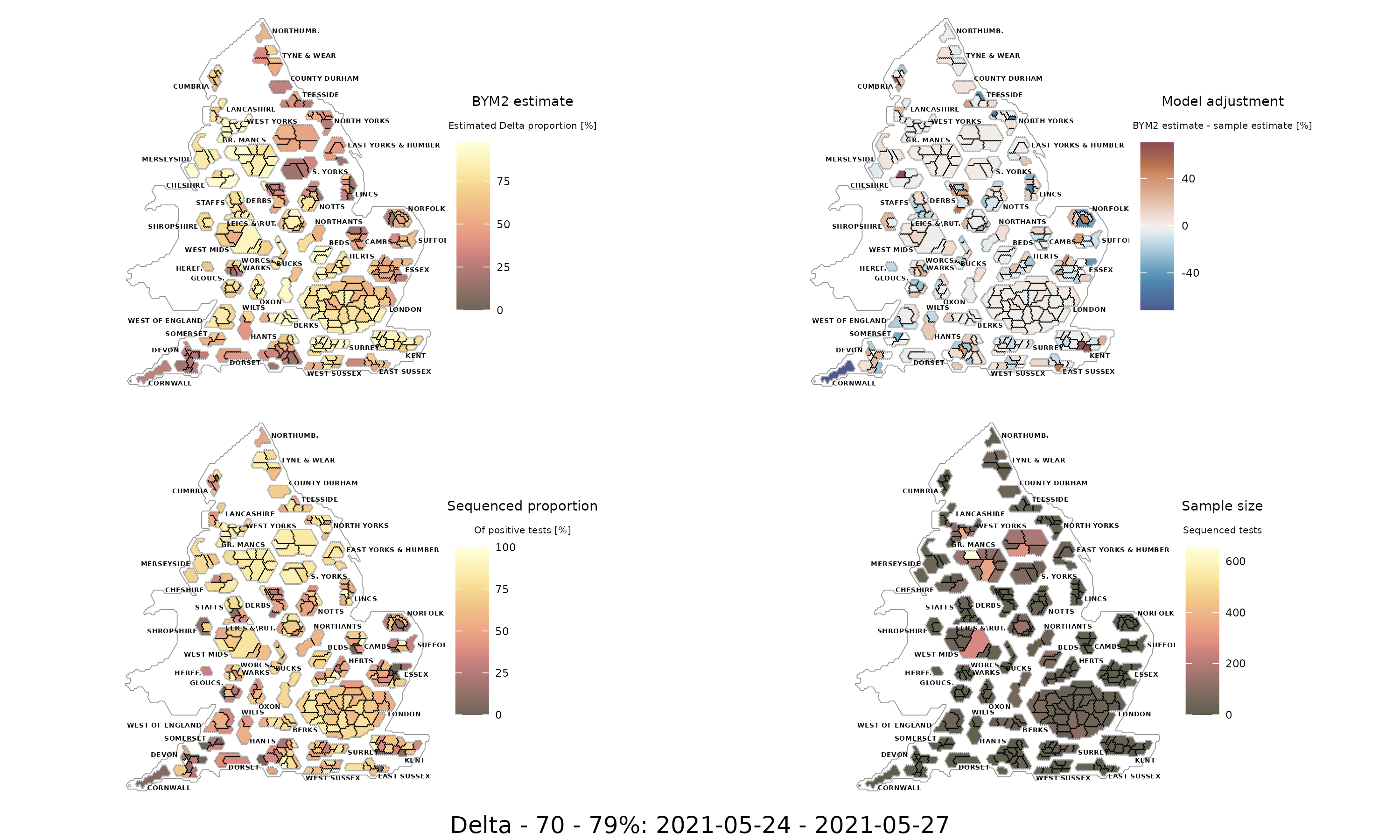


Fig A54. The BYM2 estimated model positivity of the Delta variant as a proportion of sequenced tests, the model adjustment, the proportion of tests that were sequenced, and the sample size for the time period.


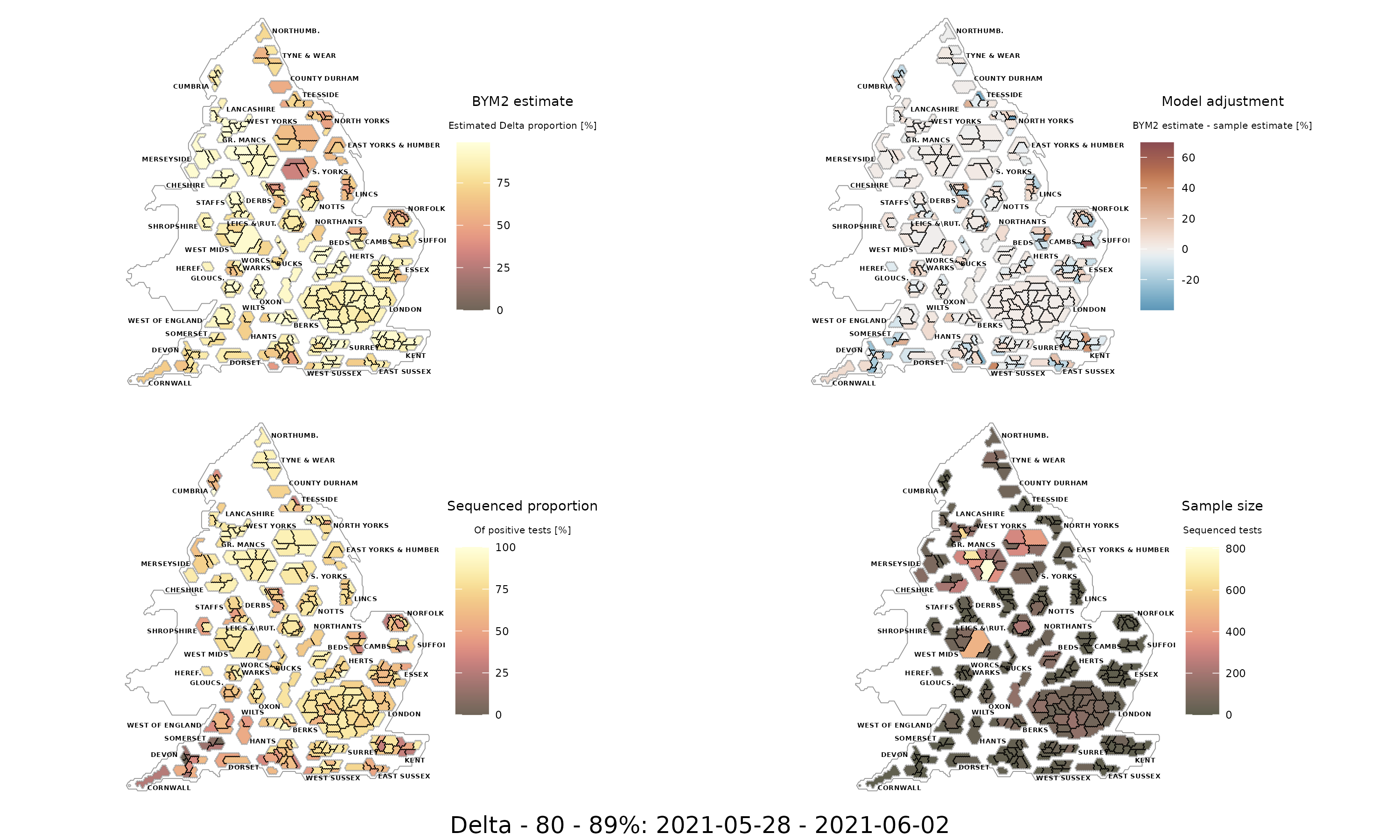


Fig A55. The BYM2 estimated model positivity of the Delta variant as a proportion of sequenced tests, the model adjustment, the proportion of tests that were sequenced, and the sample size for the time period.


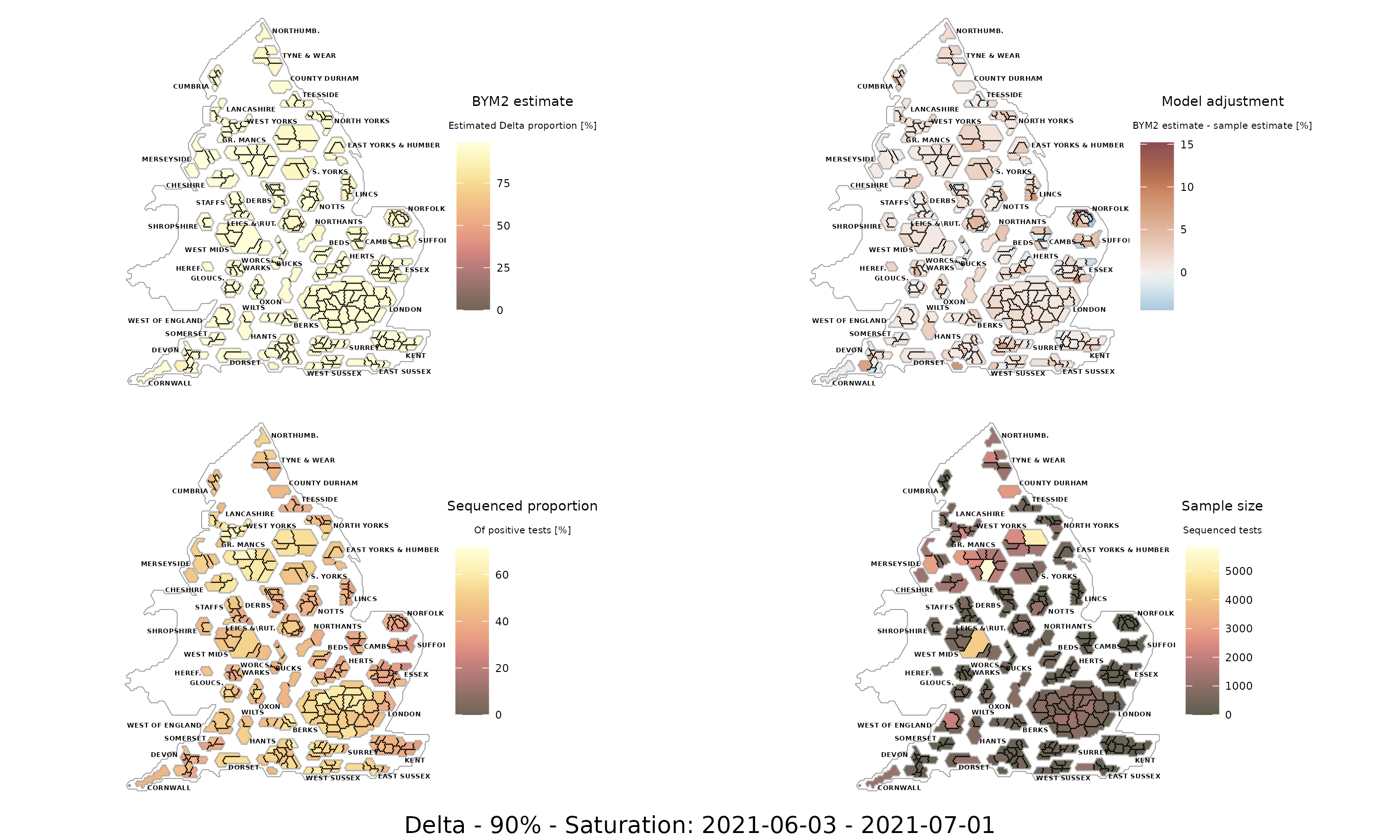


Fig A56. The BYM2 estimated model positivity of the Delta variant as a proportion of sequenced tests, the model adjustment, the proportion of tests that were sequenced, and the sample size for the time period.


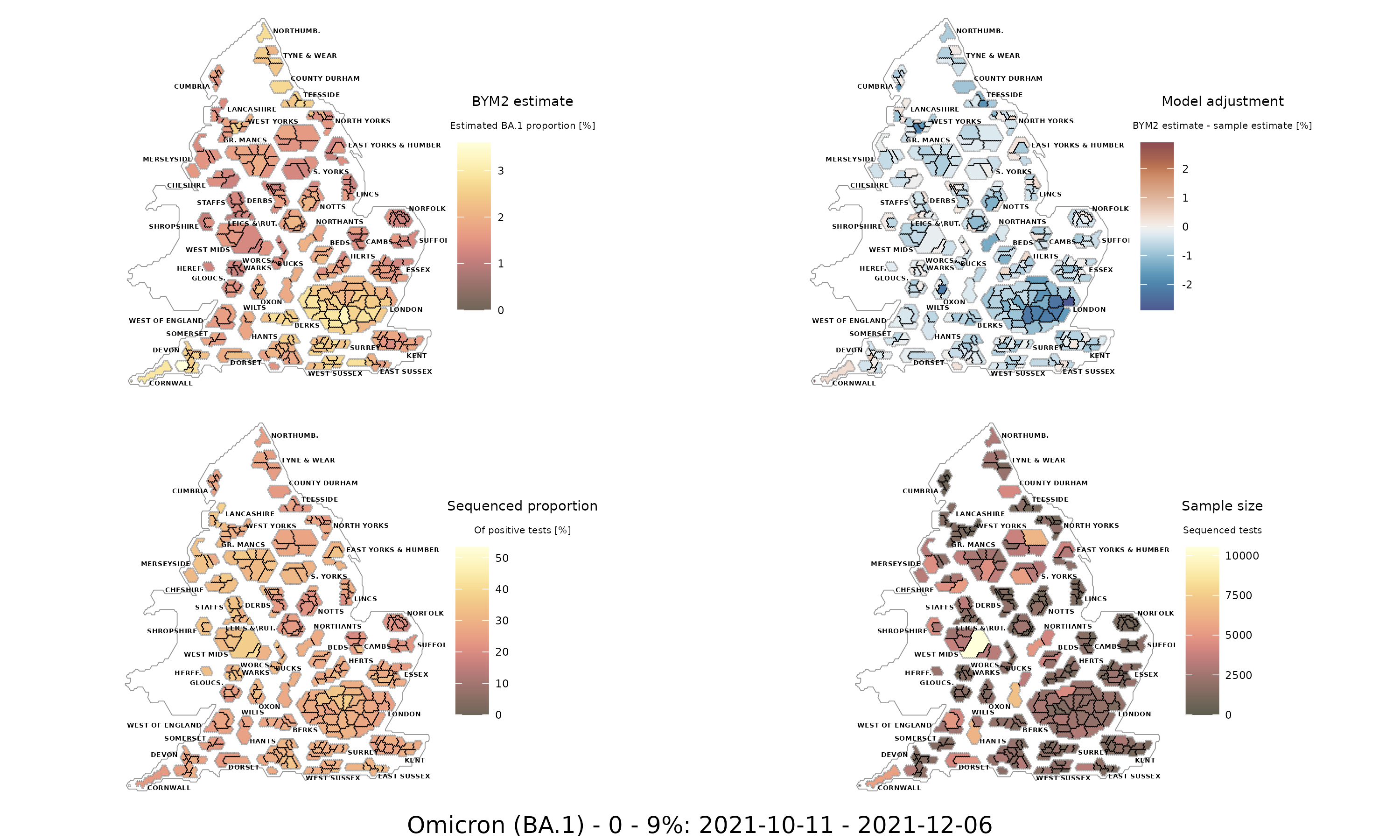


Fig A57. The BYM2 estimated model positivity of the Omicron BA.1 variant as a proportion of sequenced tests, the model adjustment, the proportion of tests that were sequenced, and the sample size for the time period.


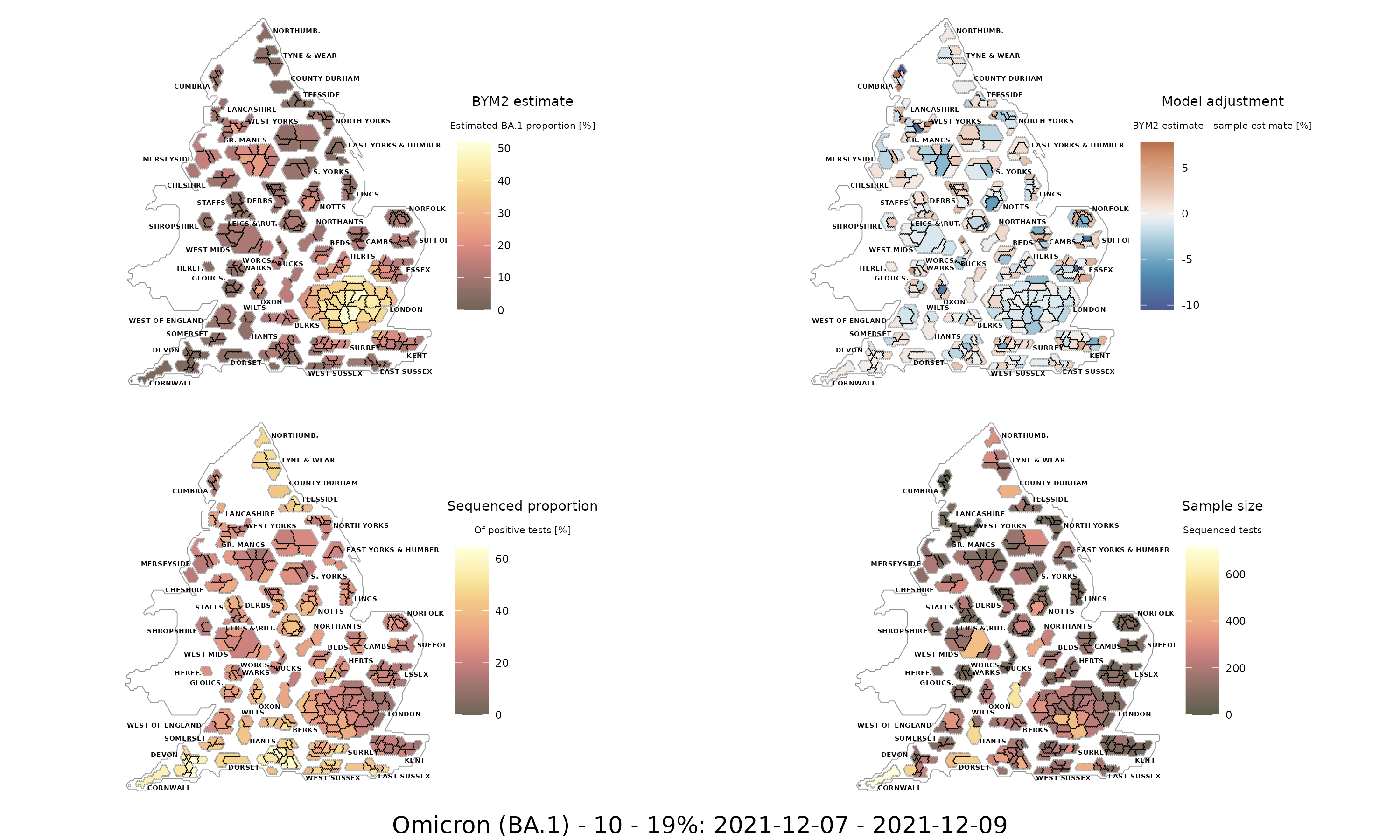


Fig A58. The BYM2 estimated model positivity of the Omicron BA.1 variant as a proportion of sequenced tests, the model adjustment, the proportion of tests that were sequenced, and the sample size for the time period.


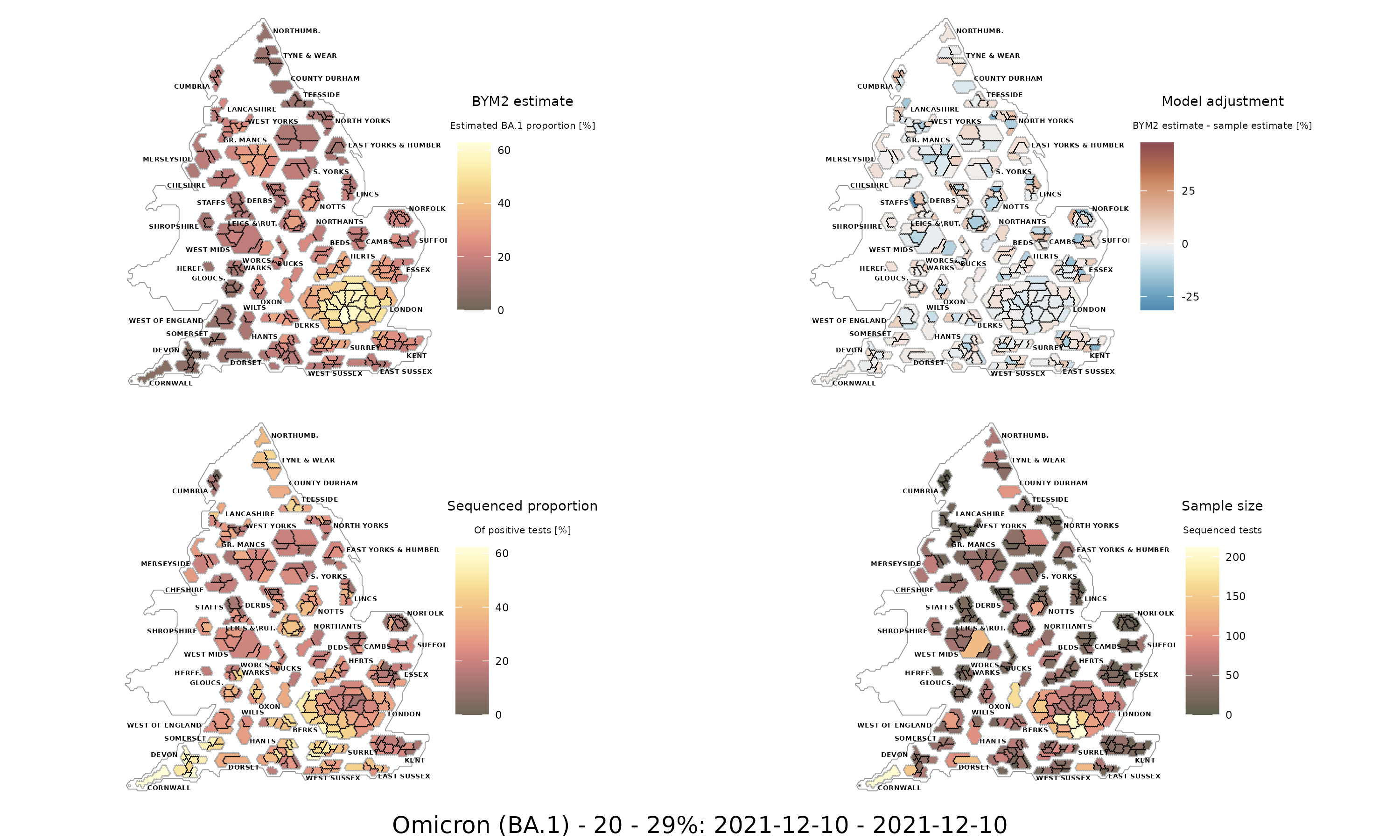


Fig A59. The BYM2 estimated model positivity of the Omicron BA.1 variant as a proportion of sequenced tests, the model adjustment, the proportion of tests that were sequenced, and the sample size for the time period.


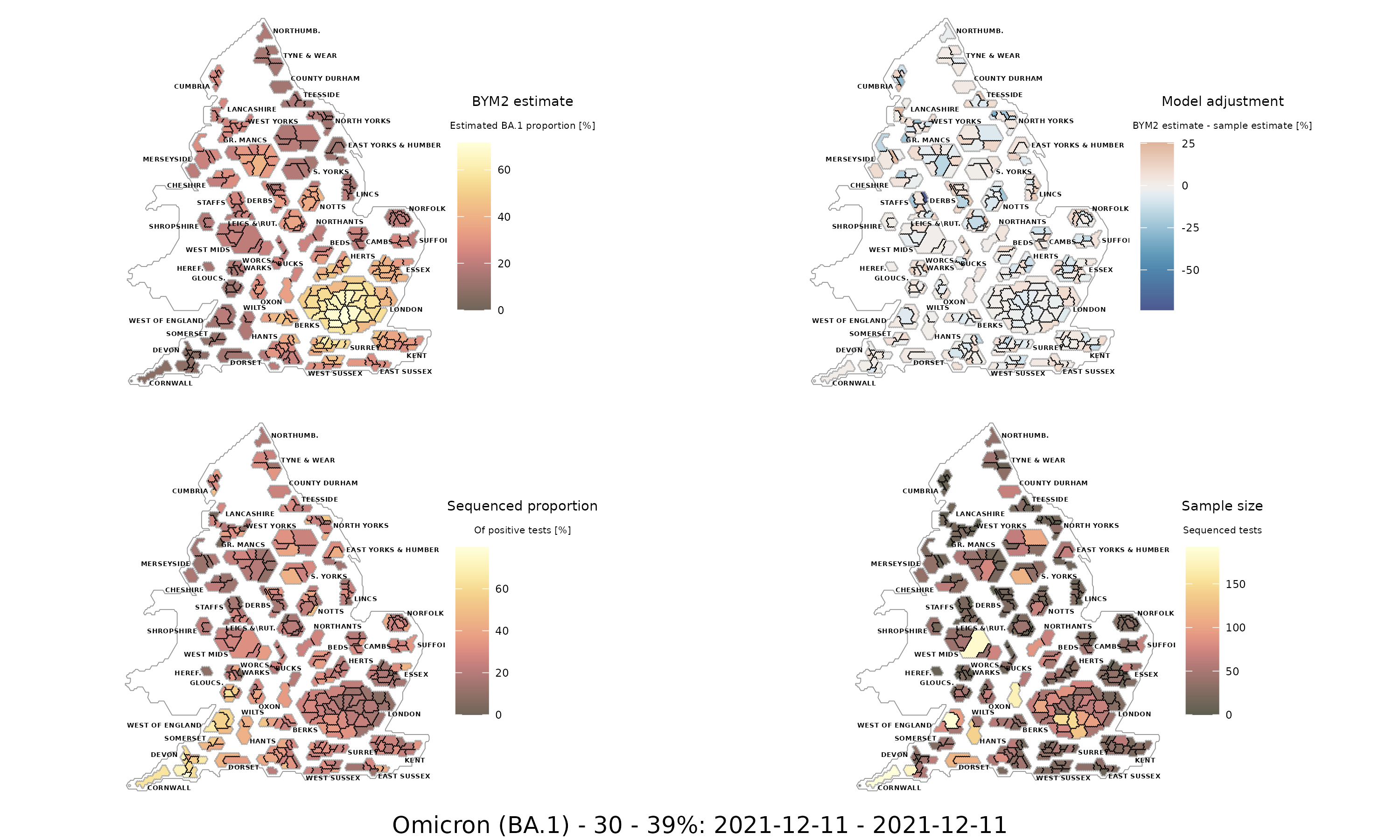


Fig A60. The BYM2 estimated model positivity of the Omicron BA.1 variant as a proportion of sequenced tests, the model adjustment, the proportion of tests that were sequenced, and the sample size for the time period.


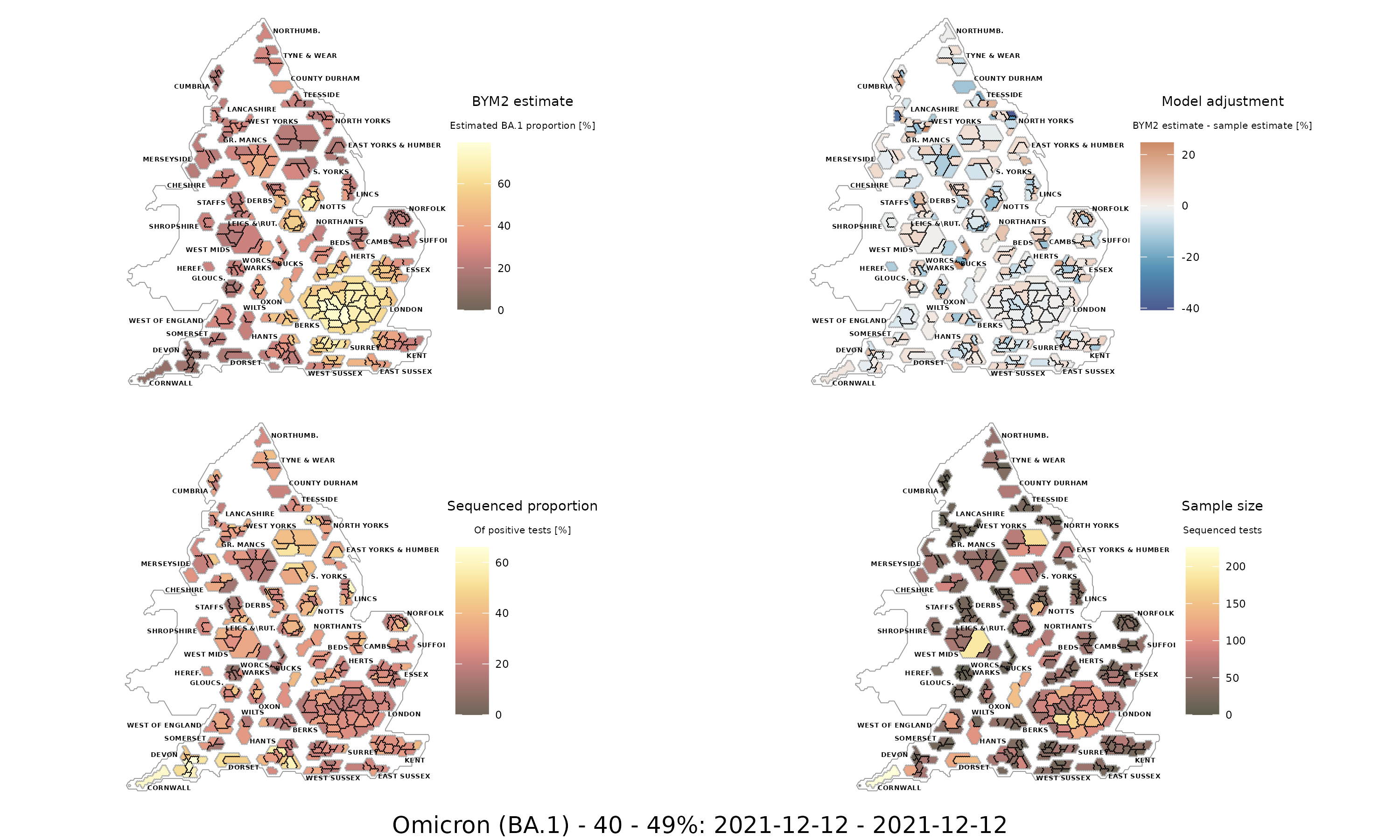


Fig A61. The BYM2 estimated model positivity of the Omicron BA.1 variant as a proportion of sequenced tests, the model adjustment, the proportion of tests that were sequenced, and the sample size for the time period.


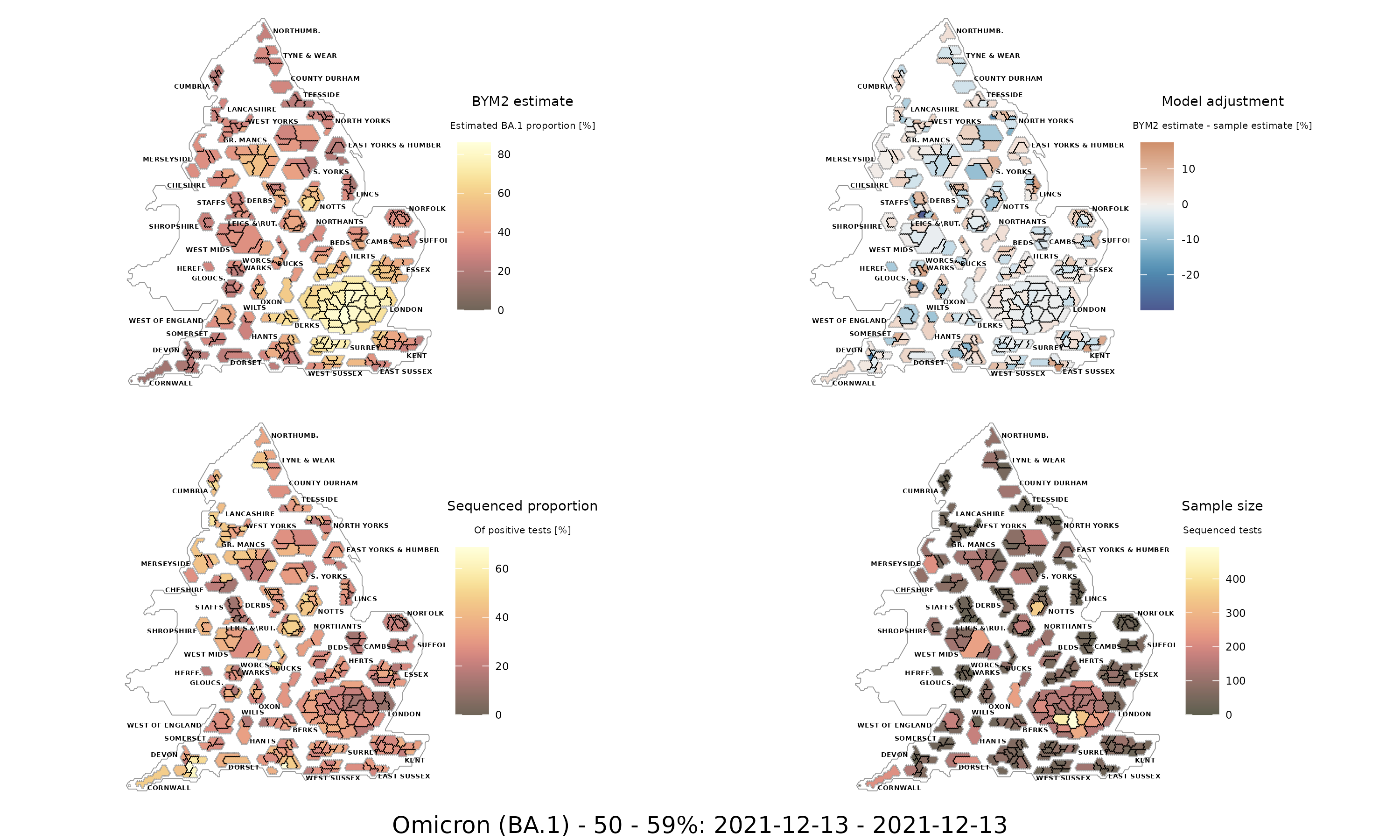


Fig A62. The BYM2 estimated model positivity of the Omicron BA.1 variant as a proportion of sequenced tests, the model adjustment, the proportion of tests that were sequenced, and the sample size for the time period.


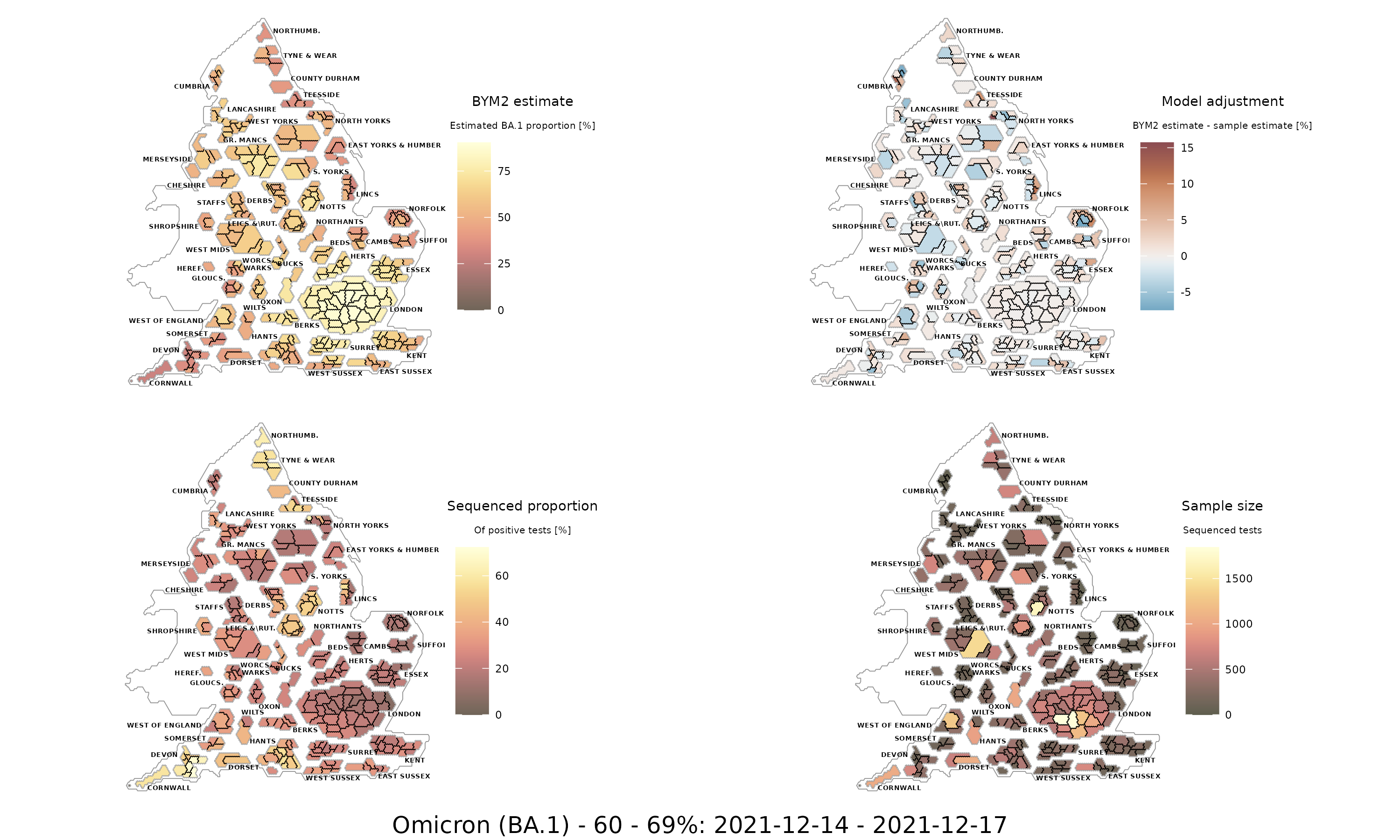


Fig A63. The BYM2 estimated model positivity of the Omicron BA.1 variant as a proportion of sequenced tests, the model adjustment, the proportion of tests that were sequenced, and the sample size for the time period.


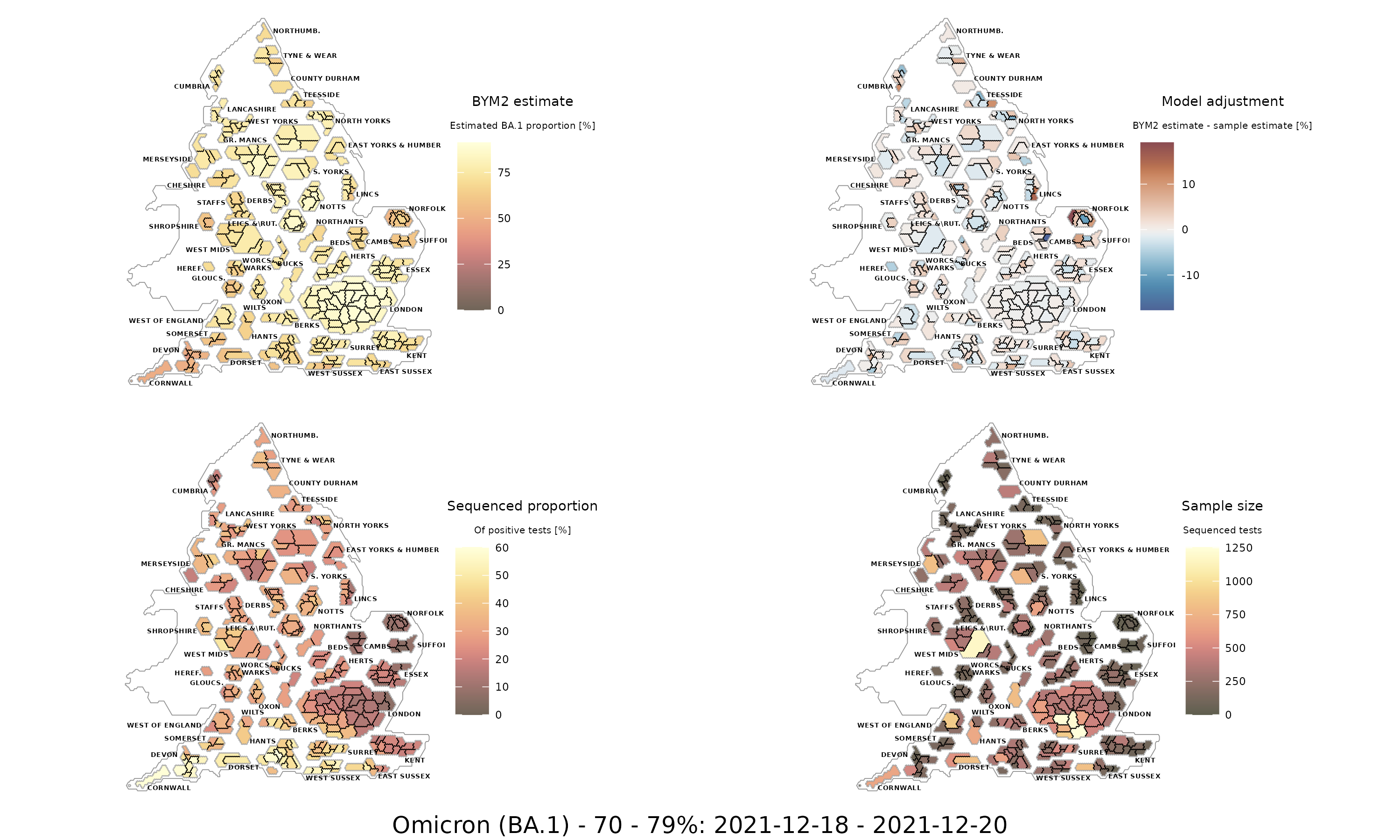


Fig A64. The BYM2 estimated model positivity of the Omicron BA.1 variant as a proportion of sequenced tests, the model adjustment, the proportion of tests that were sequenced, and the sample size for the time period.


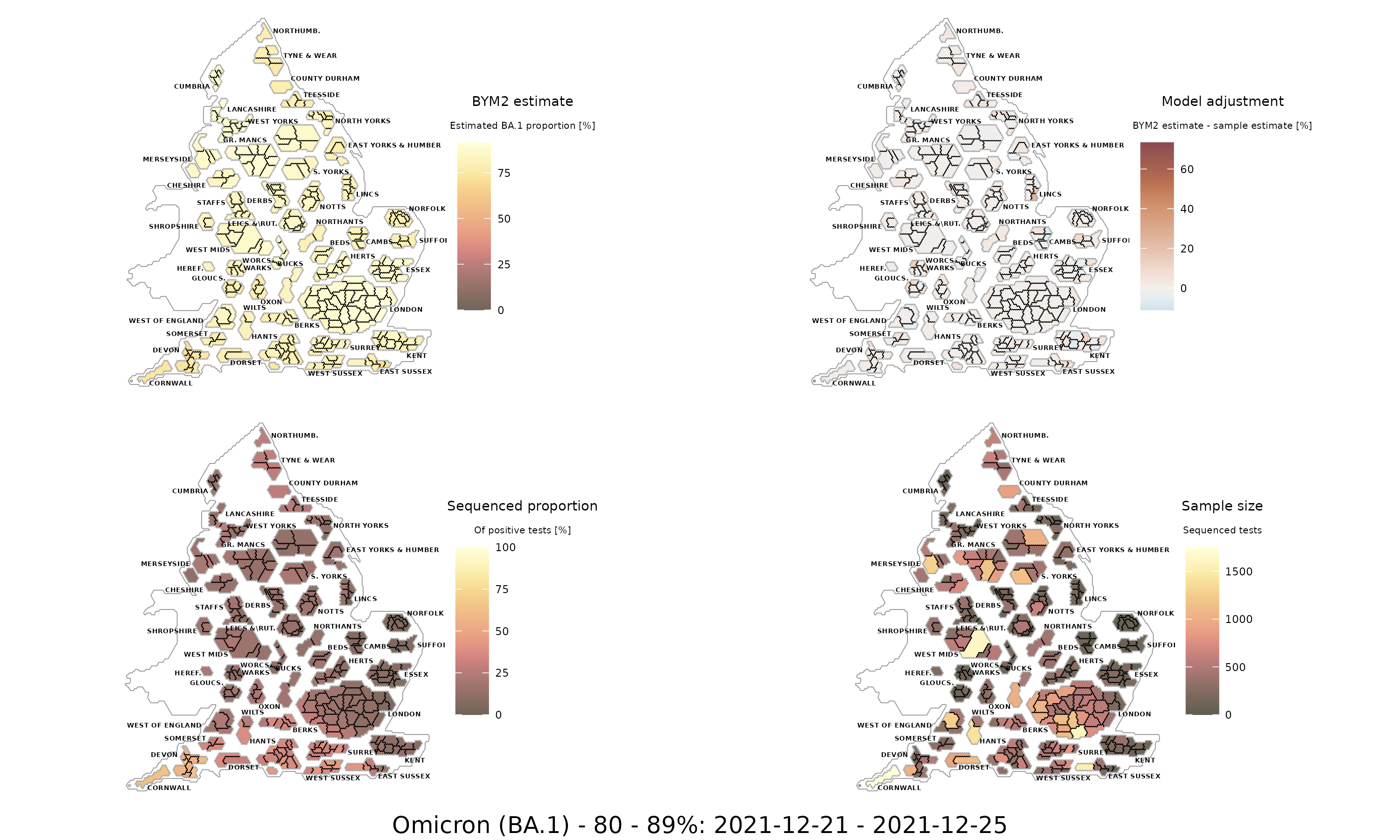


Fig A65. The BYM2 estimated model positivity of the Omicron BA.1 variant as a proportion of sequenced tests, the model adjustment, the proportion of tests that were sequenced, and the sample size for the time period.


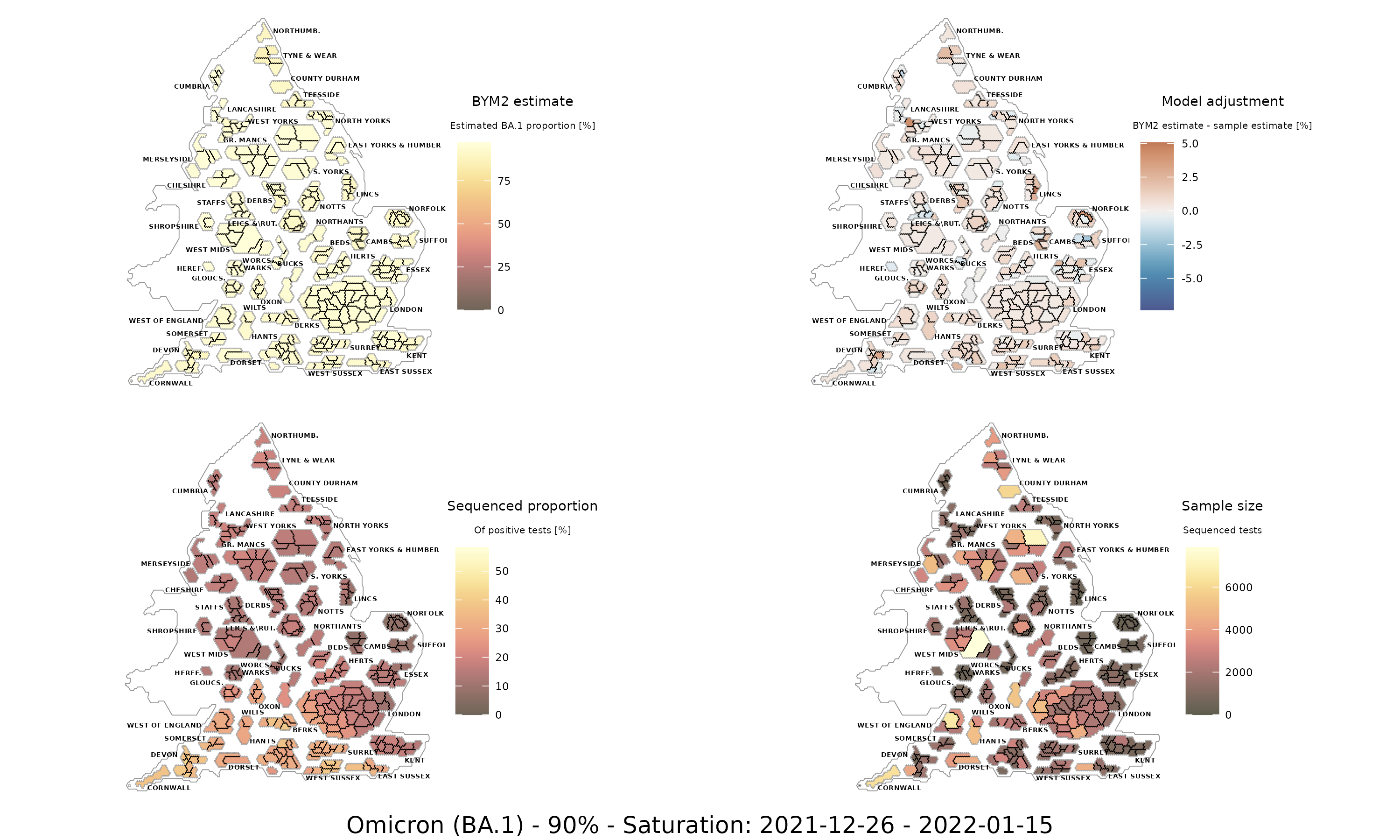


Fig A66. The BYM2 estimated model positivity of the Omicron BA.1 variant as a proportion of sequenced tests, the model adjustment, the proportion of tests that were sequenced, and the sample size for the time period.


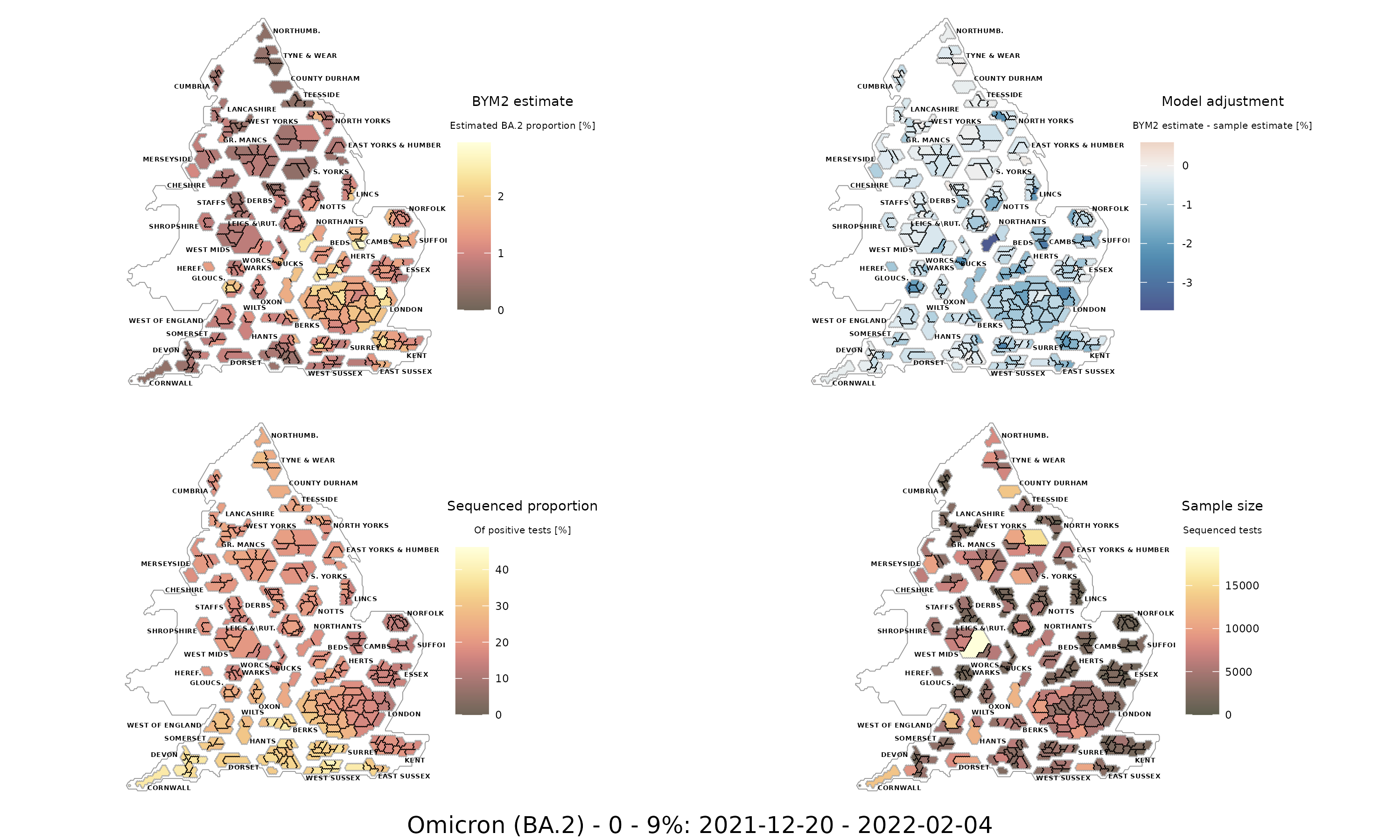


Fig A67. The BYM2 estimated model positivity of the Omicron BA.2 variant as a proportion of sequenced tests, the model adjustment, the proportion of tests that were sequenced, and the sample size for the time period.


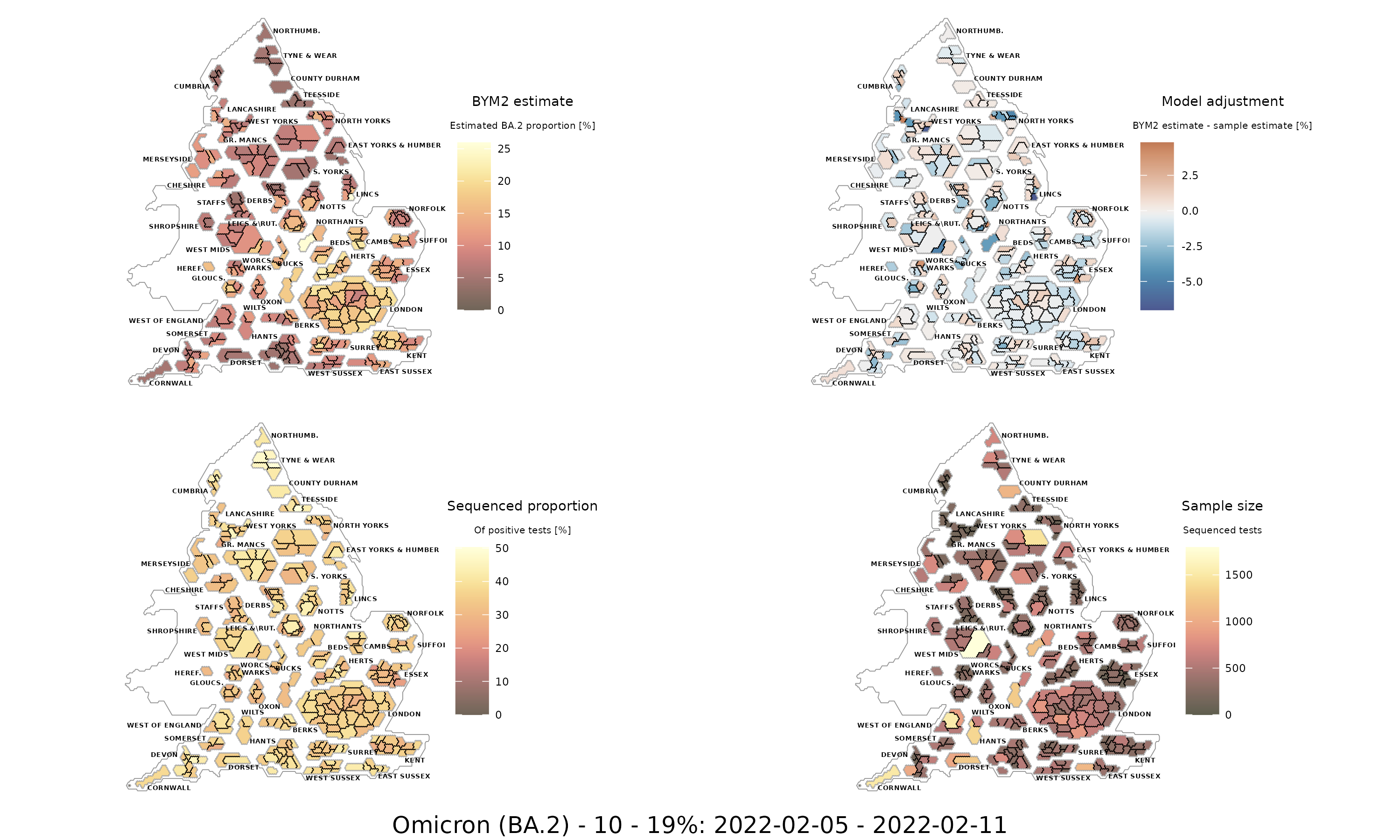


Fig A68. The BYM2 estimated model positivity of the Omicron BA.2 variant as a proportion of sequenced tests, the model adjustment, the proportion of tests that were sequenced, and the sample size for the time period.


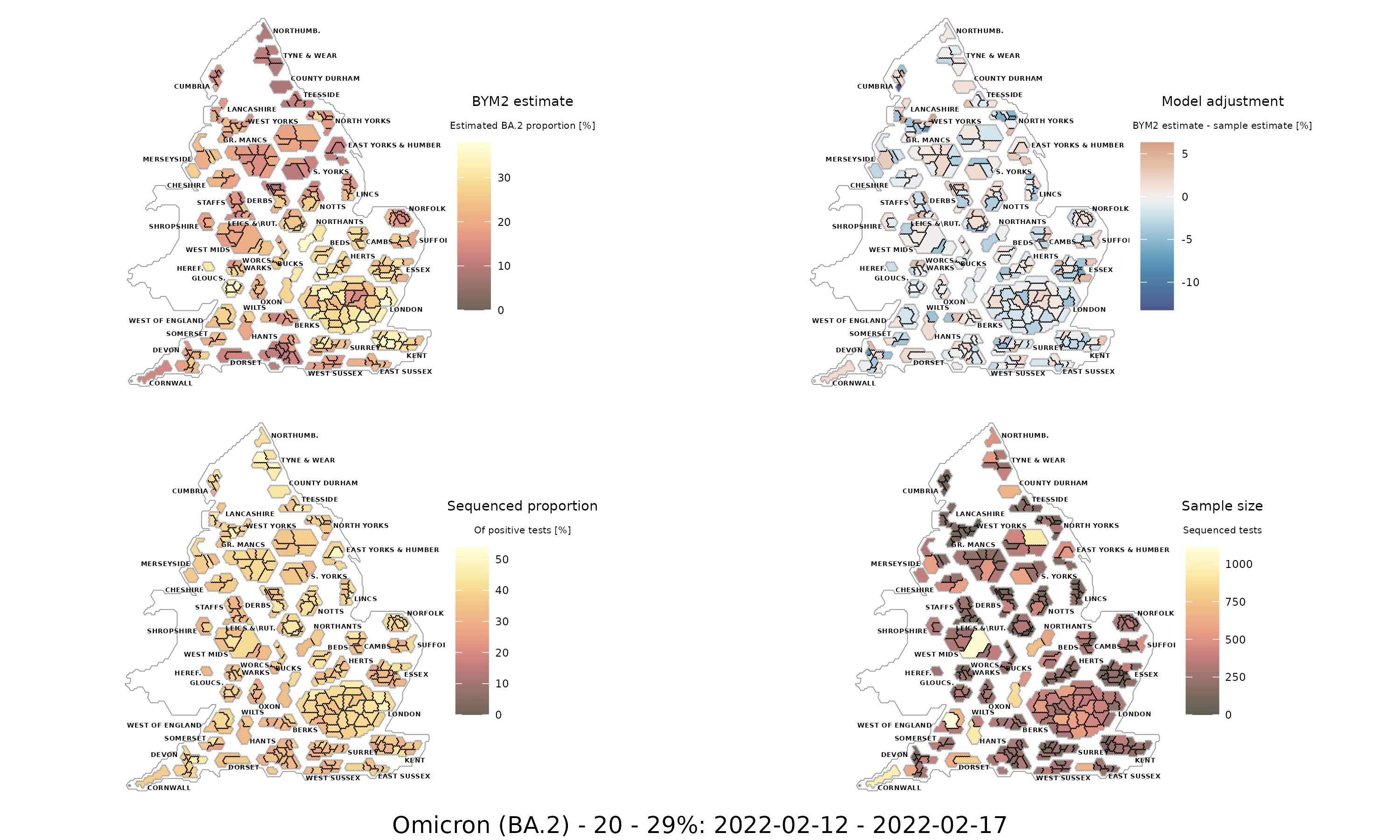


Fig A69. The BYM2 estimated model positivity of the Omicron BA.2 variant as a proportion of sequenced tests, the model adjustment, the proportion of tests that were sequenced, and the sample size for the time period.


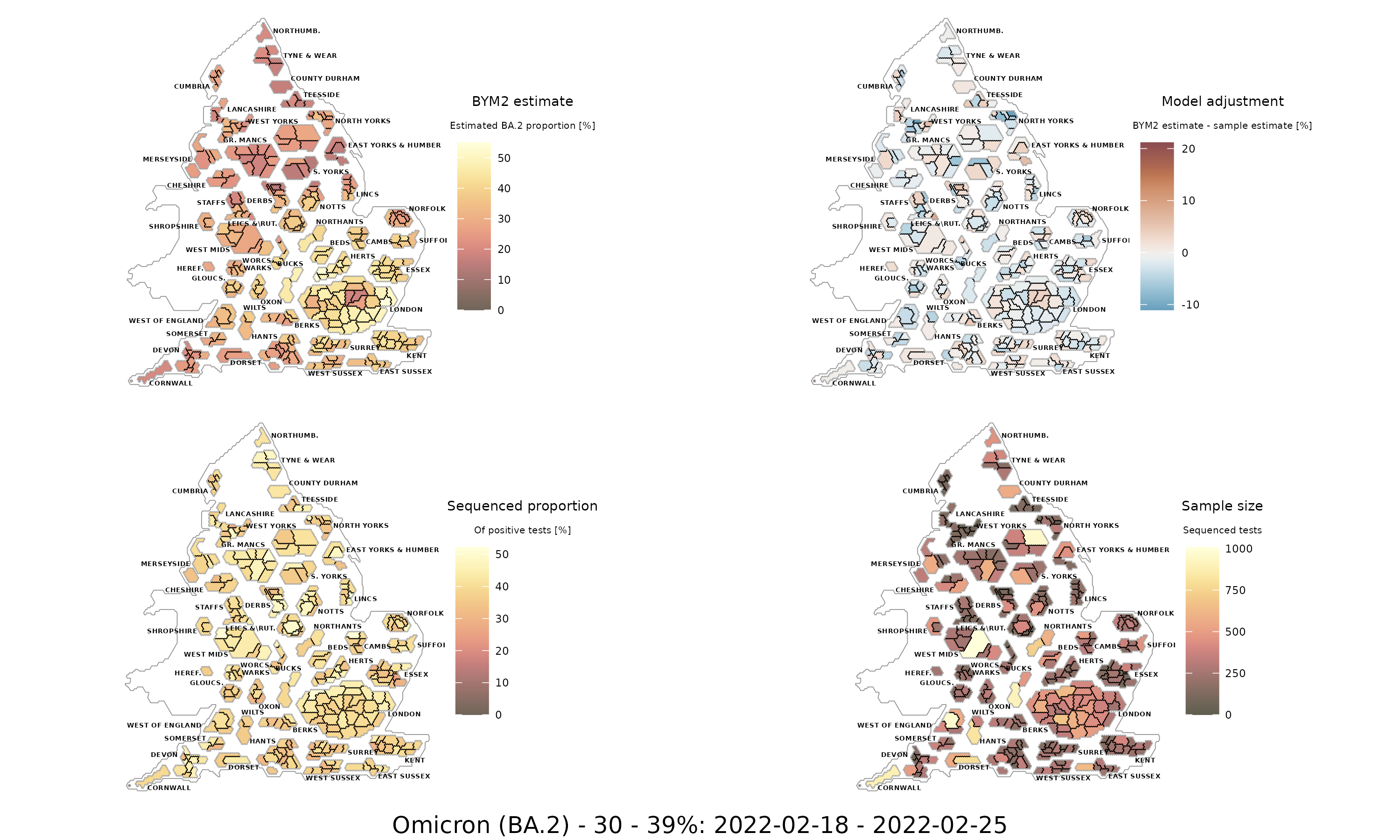


Fig A70. The BYM2 estimated model positivity of the Omicron BA.2 variant as a proportion of sequenced tests, the model adjustment, the proportion of tests that were sequenced, and the sample size for the time period.


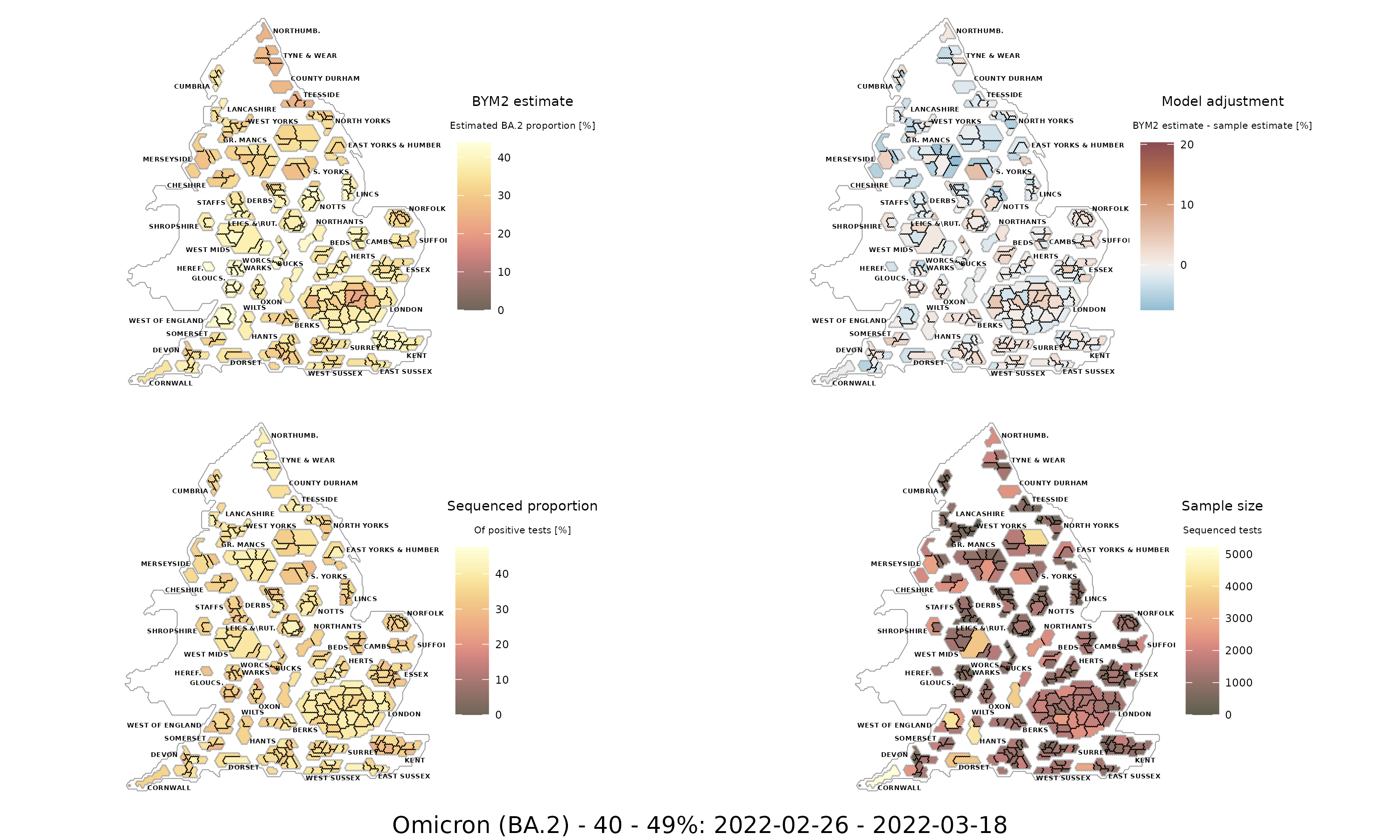


Fig A71. The BYM2 estimated model positivity of the Omicron BA.2 variant as a proportion of sequenced tests, the model adjustment, the proportion of tests that were sequenced, and the sample size for the time period.


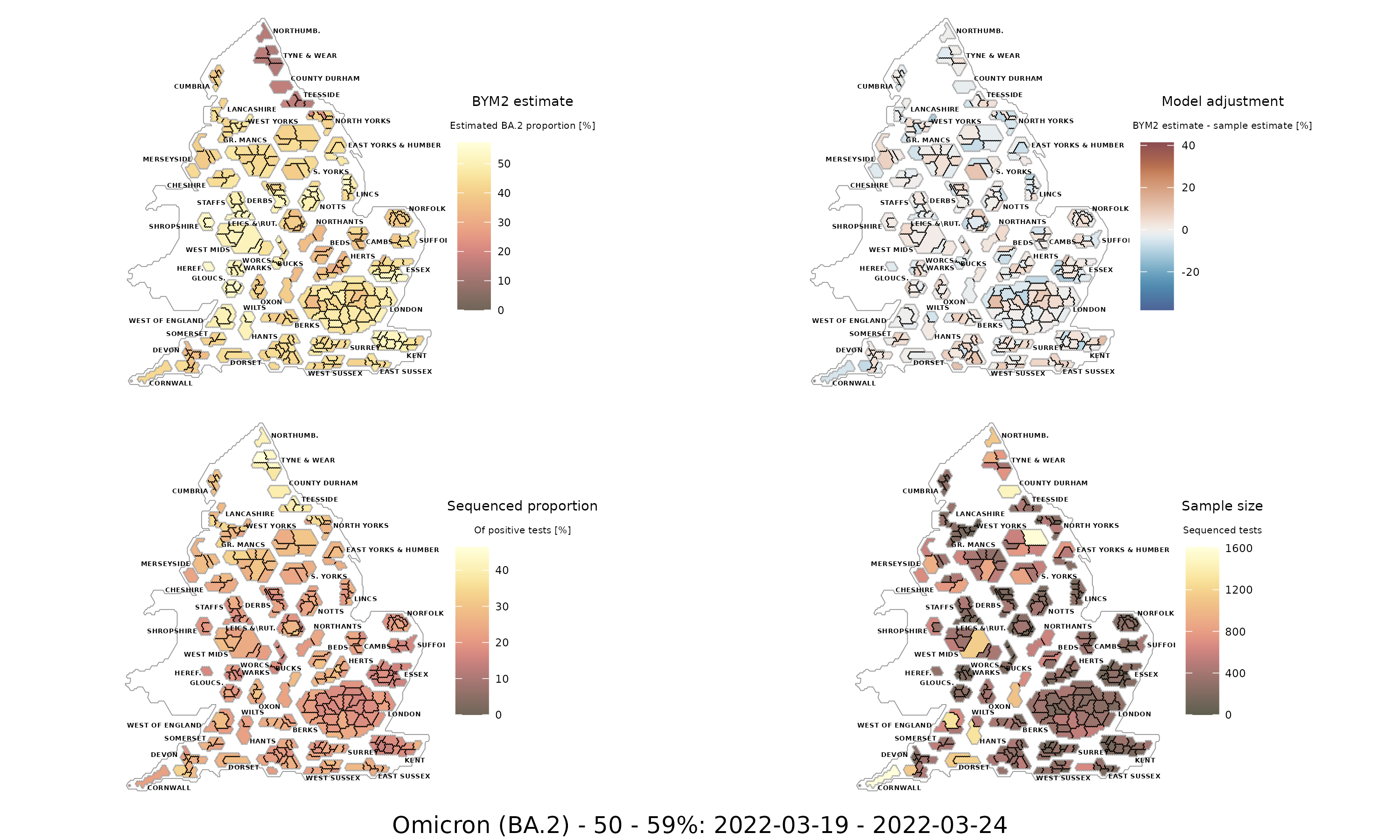


Fig A72. The BYM2 estimated model positivity of the Omicron BA.2 variant as a proportion of sequenced tests, the model adjustment, the proportion of tests that were sequenced, and the sample size for the time period.


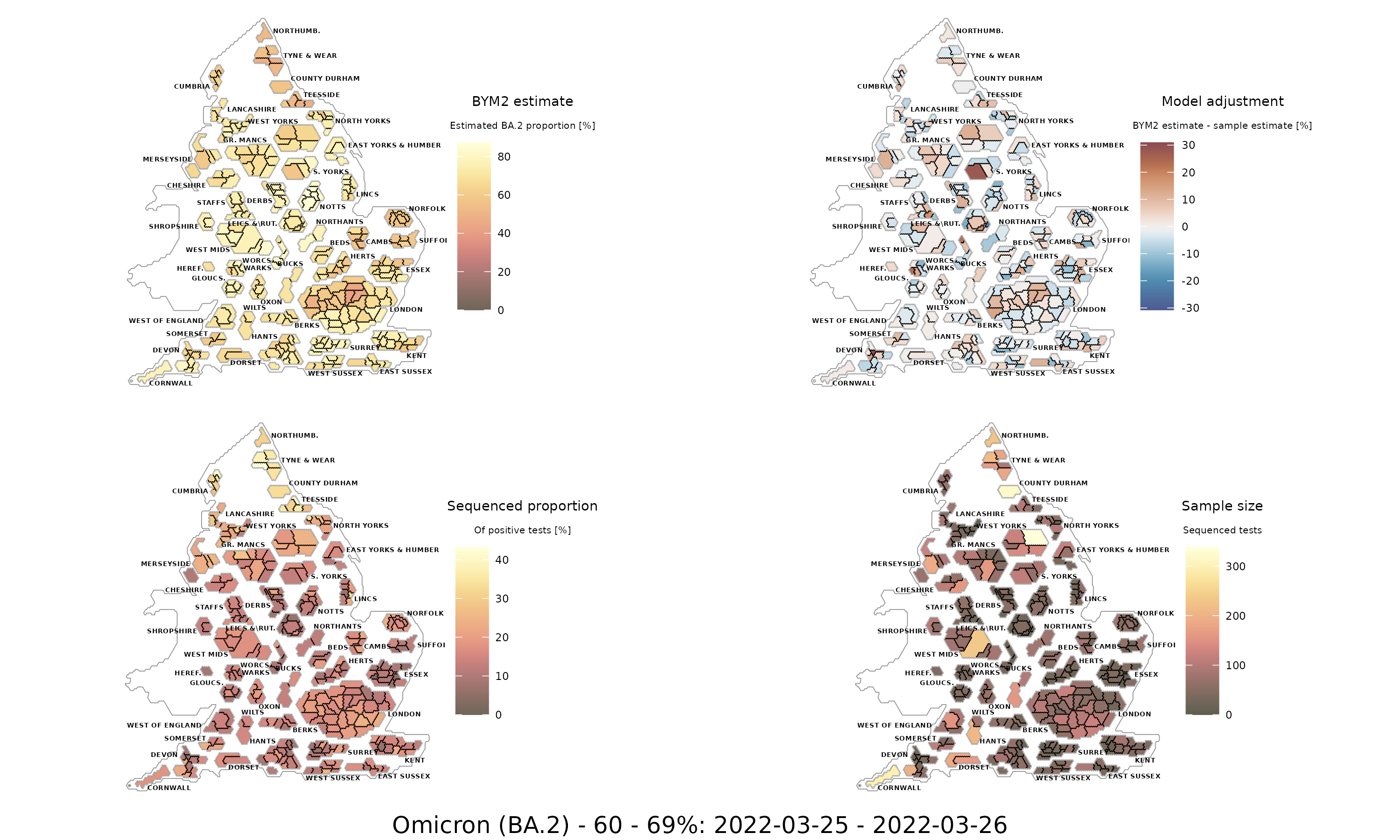


Fig A73. The BYM2 estimated model positivity of the Omicron BA.2 variant as a proportion of sequenced tests, the model adjustment, the proportion of tests that were sequenced, and the sample size for the time period.


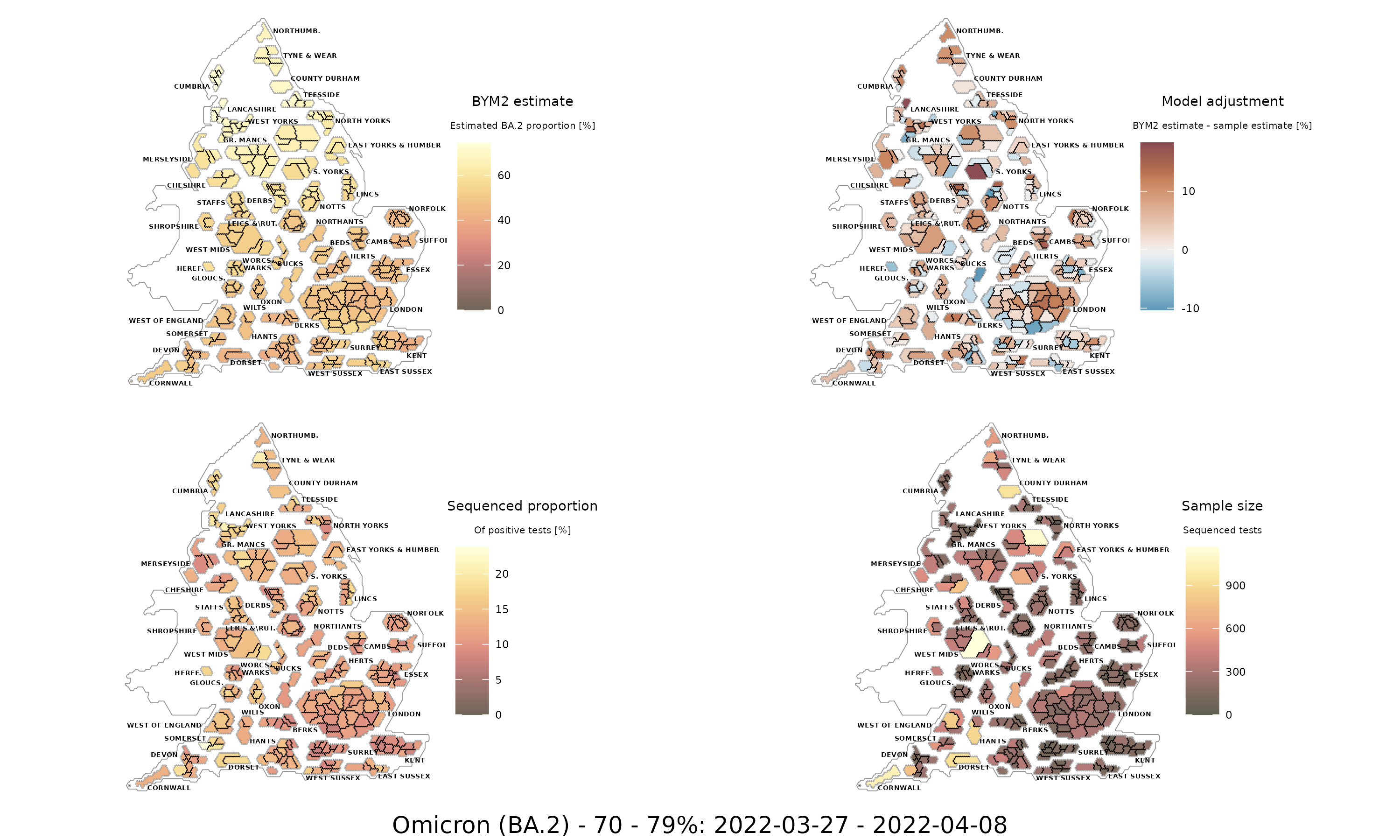


Fig A74. The BYM2 estimated model positivity of the Omicron BA.2 variant as a proportion of sequenced tests, the model adjustment, the proportion of tests that were sequenced, and the sample size for the time period.


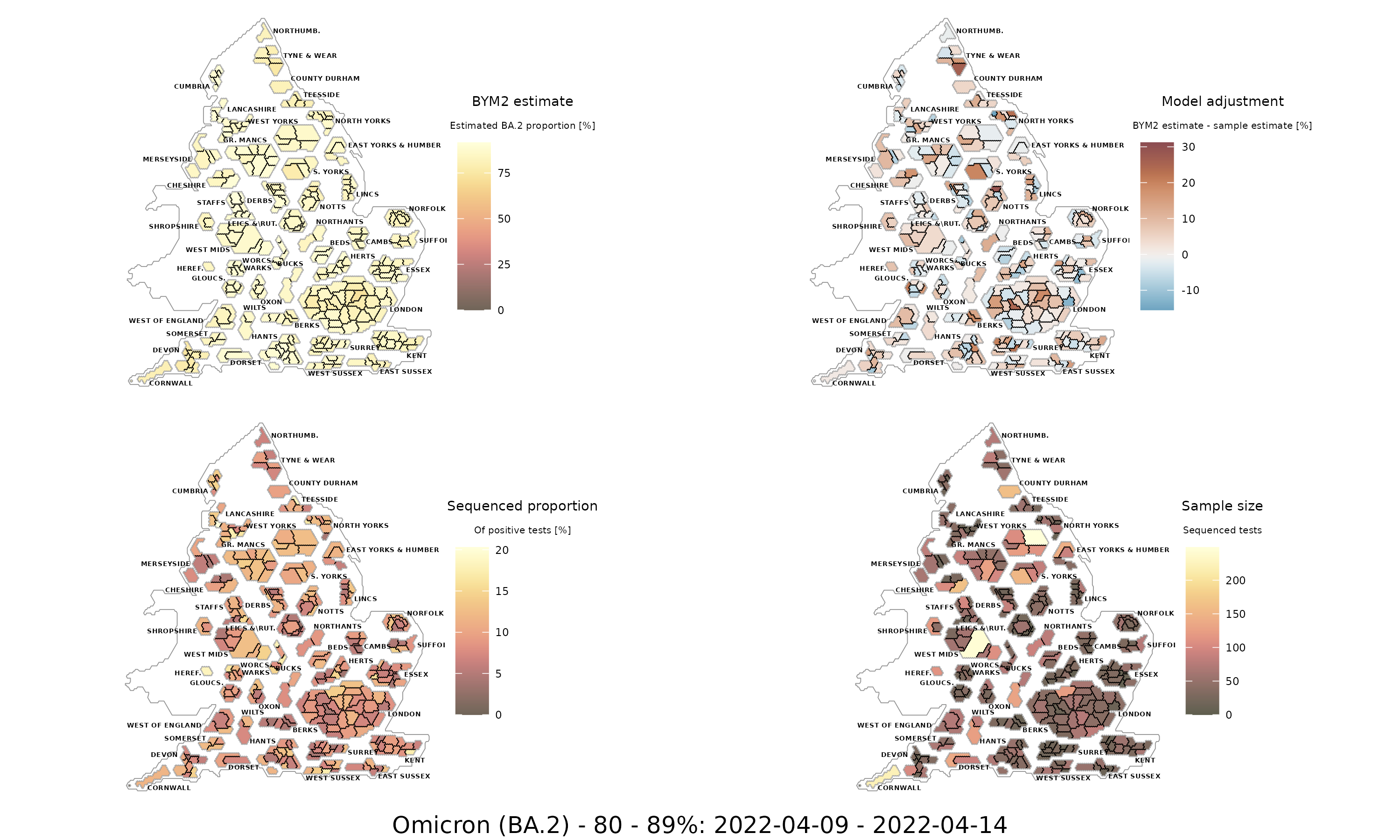


Fig A75. The BYM2 estimated model positivity of the Omicron BA.2 variant as a proportion of sequenced tests, the model adjustment, the proportion of tests that were sequenced, and the sample size for the time period.


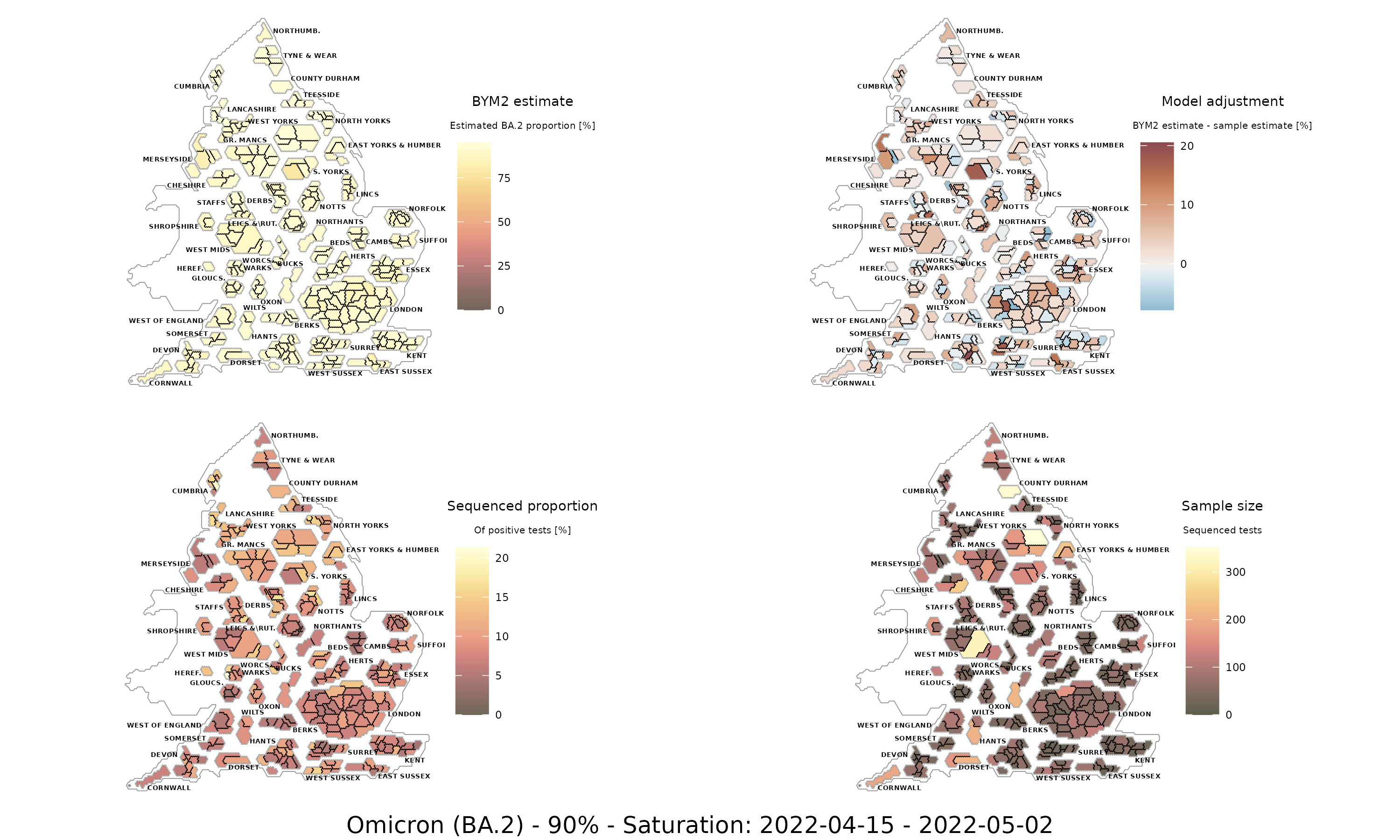


Fig A76. The BYM2 estimated model positivity of the Omicron BA.2 variant as a proportion of sequenced tests, the model adjustment, the proportion of tests that were sequenced, and the sample size for the time period.


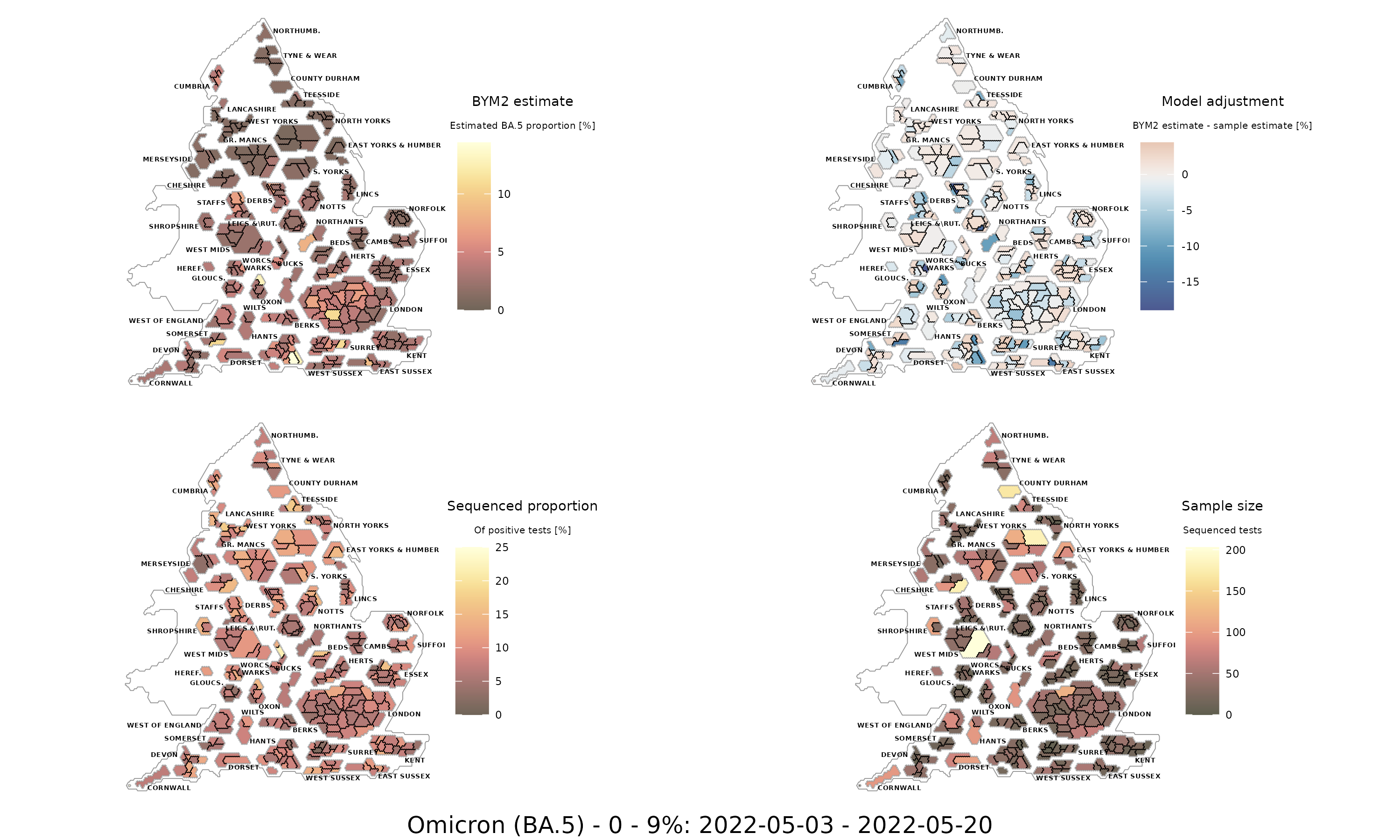


Fig A77. The BYM2 estimated model positivity of the Omicron BA.5 variant as a proportion of sequenced tests, the model adjustment, the proportion of tests that were sequenced, and the sample size for the time period.


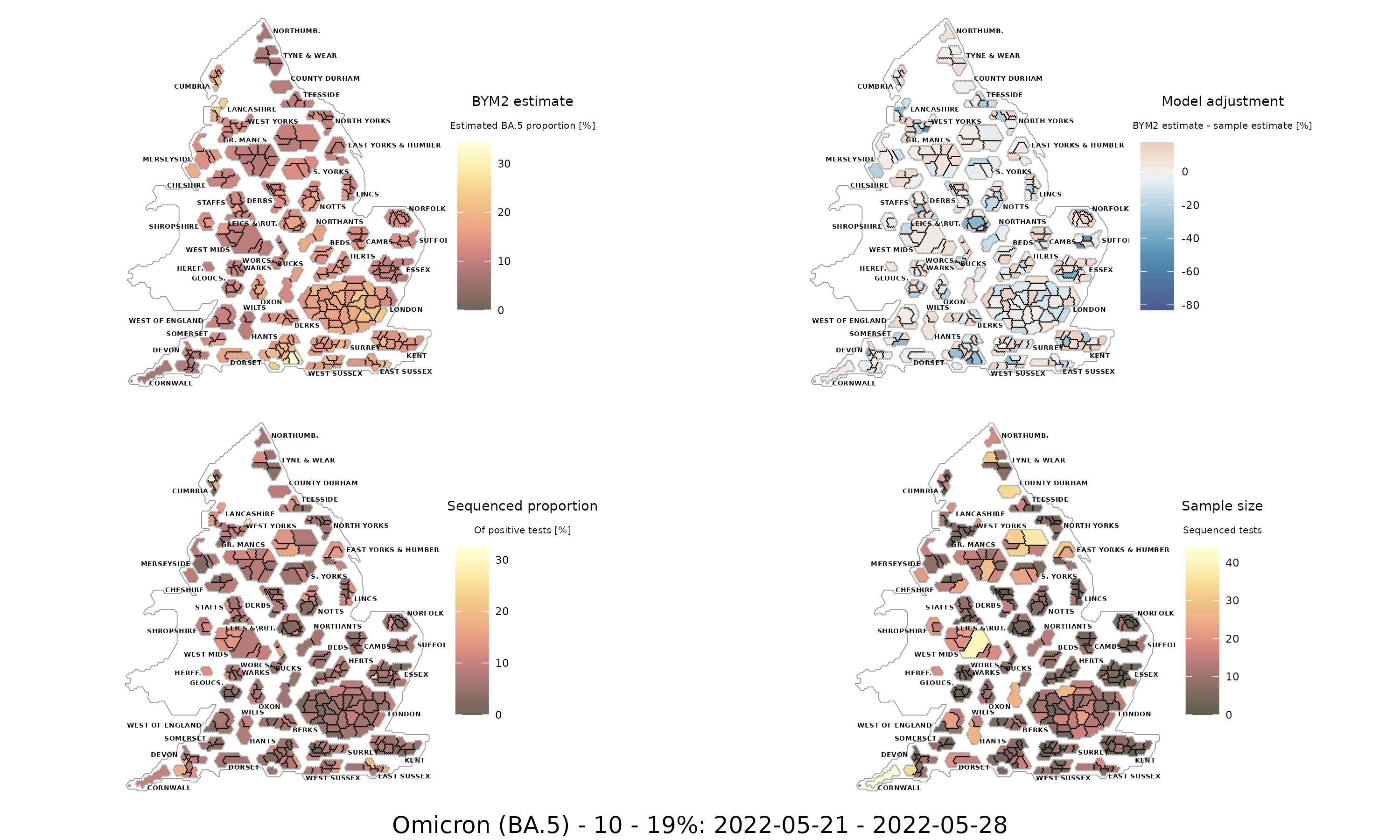


Fig A78. The BYM2 estimated model positivity of the Omicron BA.5 variant as a proportion of sequenced tests, the model adjustment, the proportion of tests that were sequenced, and the sample size for the time period.


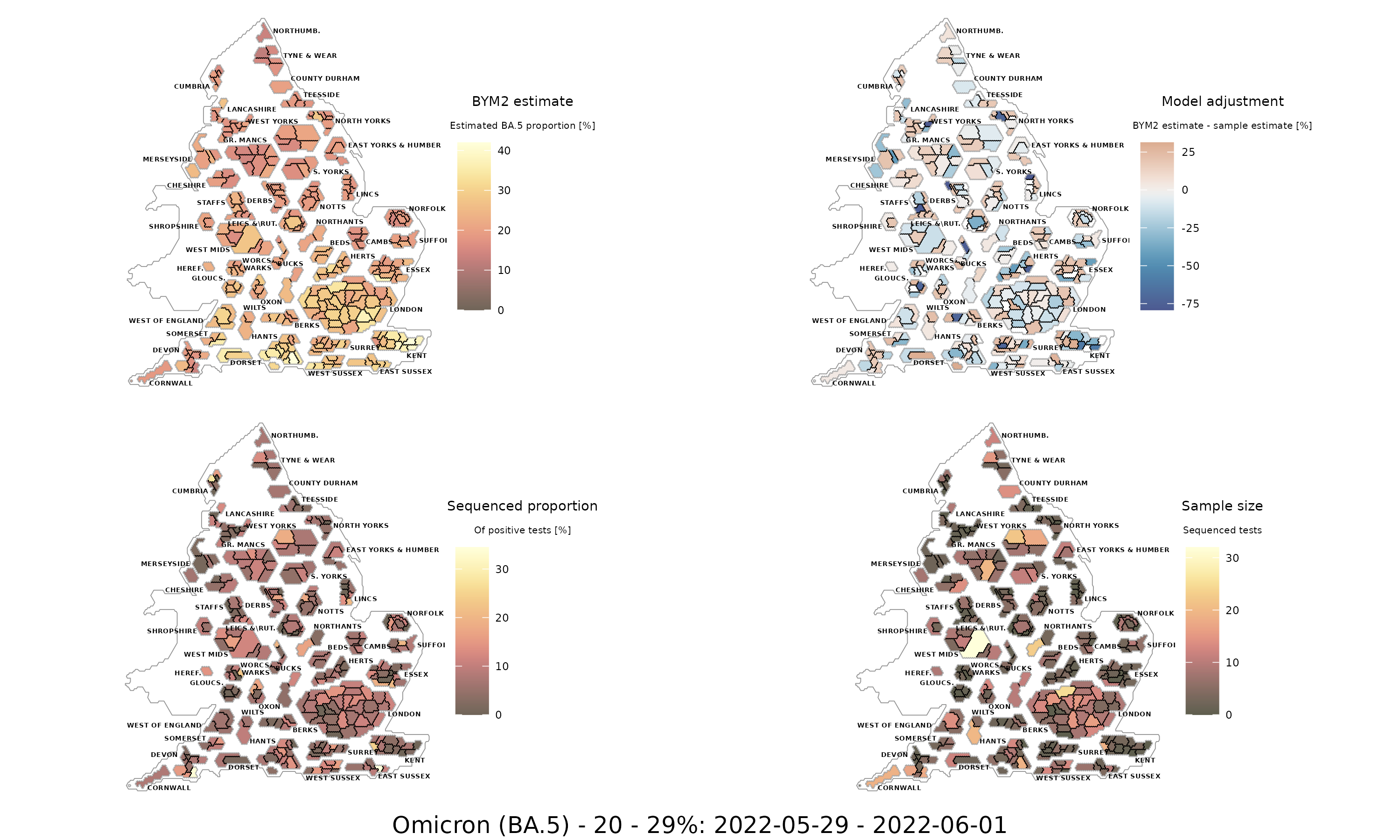


Fig A79. The BYM2 estimated model positivity of the Omicron BA.5 variant as a proportion of sequenced tests, the model adjustment, the proportion of tests that were sequenced, and the sample size for the time period.


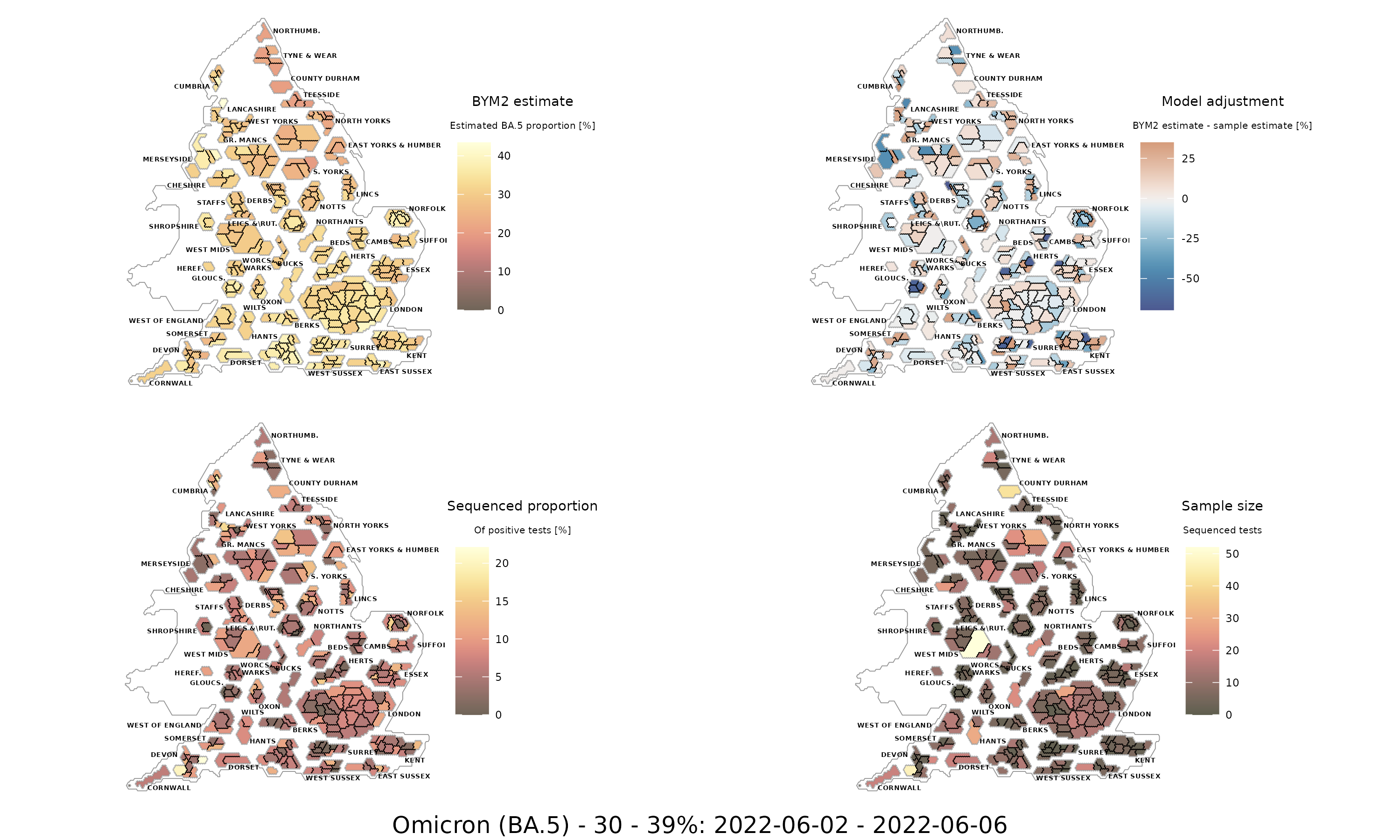


Fig A80. The BYM2 estimated model positivity of the Omicron BA.5 variant as a proportion of sequenced tests, the model adjustment, the proportion of tests that were sequenced, and the sample size for the time period.


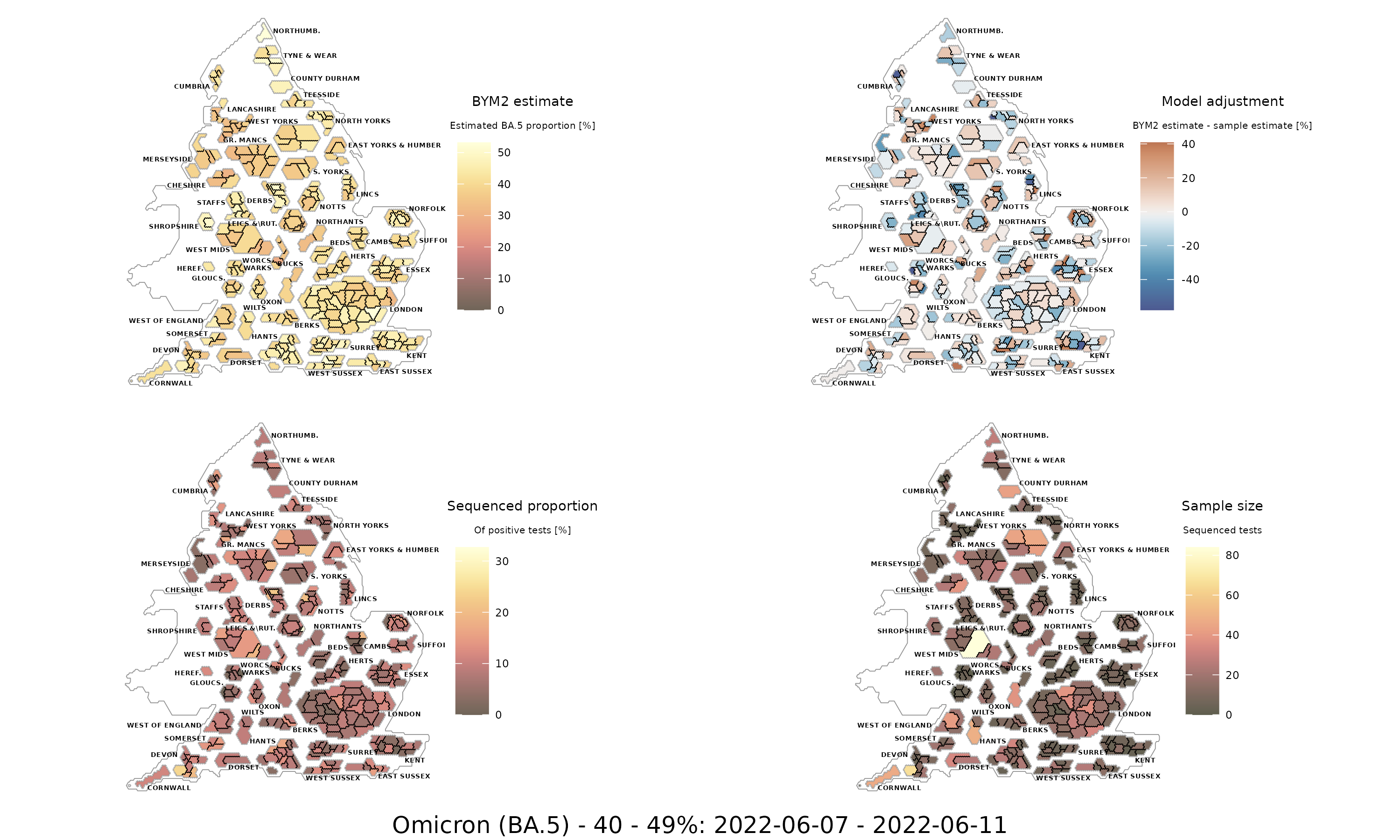


Fig A81. The BYM2 estimated model positivity of the Omicron BA.5 variant as a proportion of sequenced tests, the model adjustment, the proportion of tests that were sequenced, and the sample size for the time period.


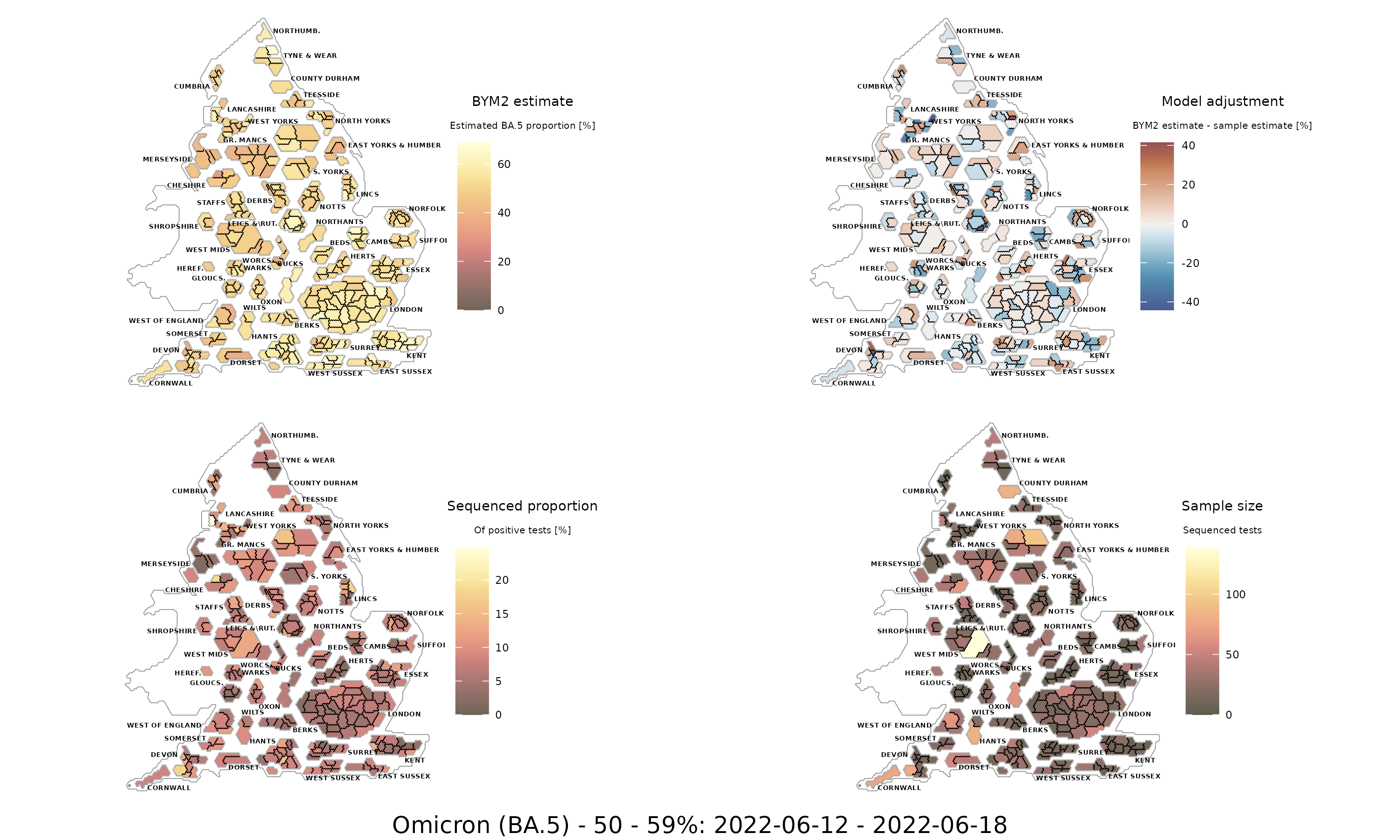


Fig A82. The BYM2 estimated model positivity of the Omicron BA.5 variant as a proportion of sequenced tests, the model adjustment, the proportion of tests that were sequenced, and the sample size for the time period.


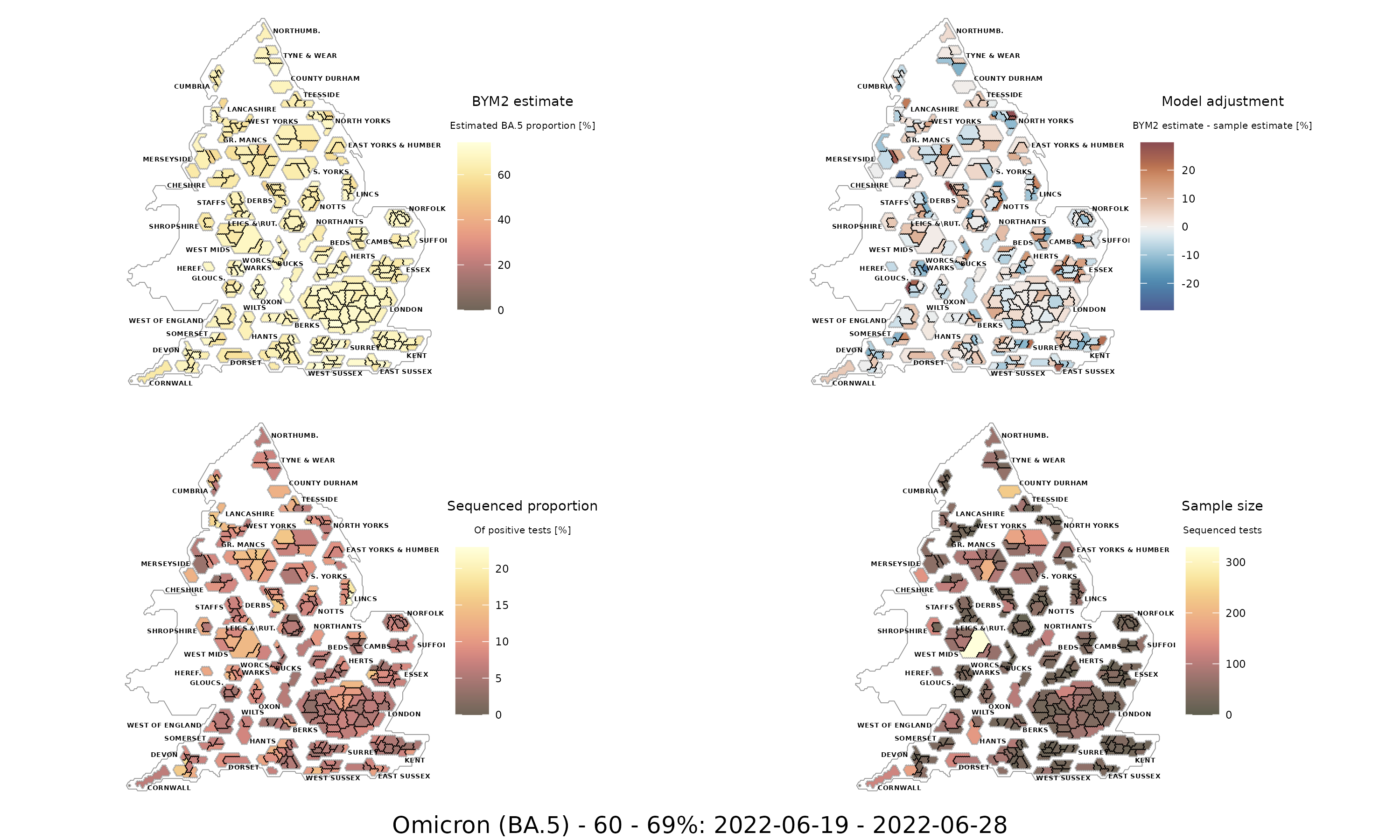


Fig A83. The BYM2 estimated model positivity of the Omicron BA.5 variant as a proportion of sequenced tests, the model adjustment, the proportion of tests that were sequenced, and the sample size for the time period.


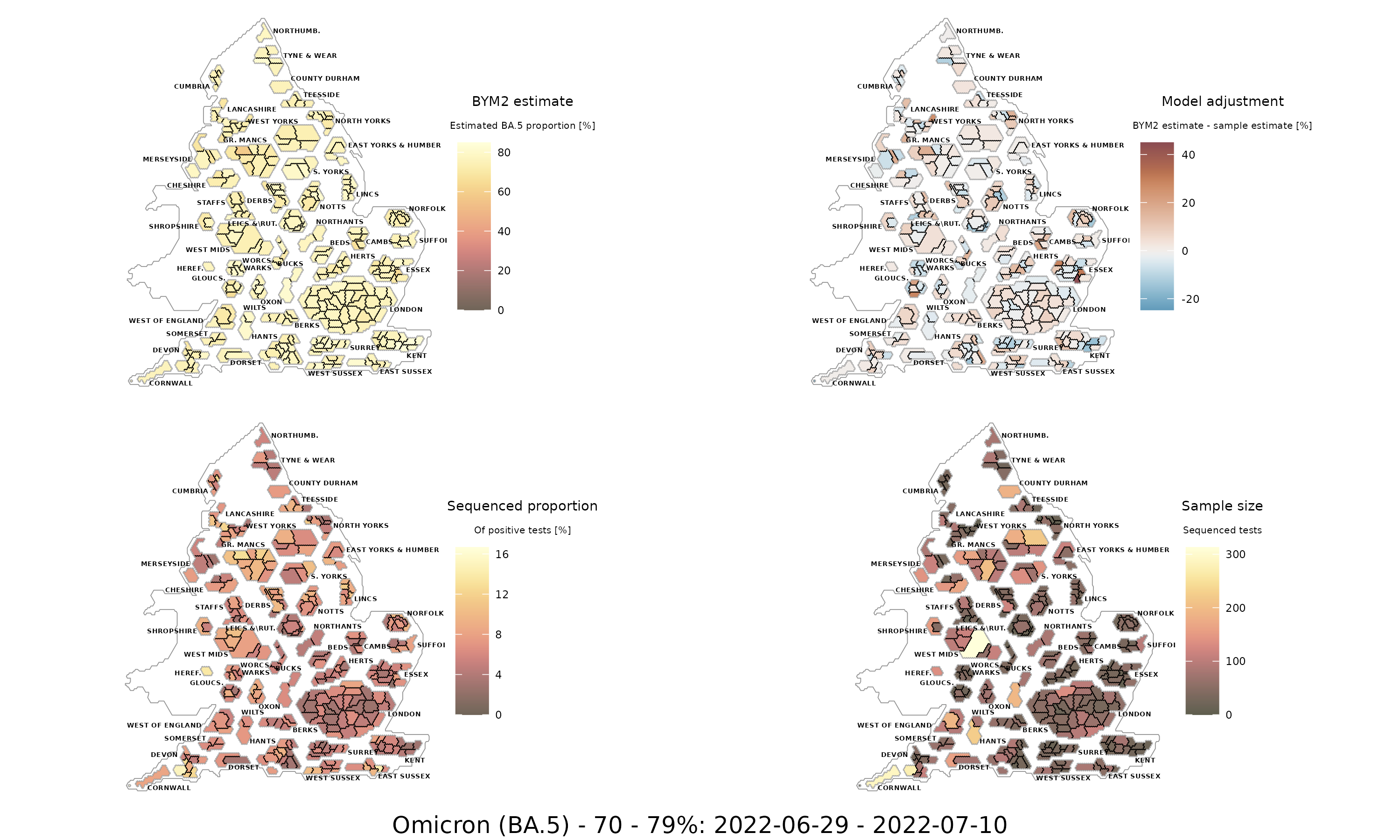


Fig A84. The BYM2 estimated model positivity of the Omicron BA.5 variant as a proportion of sequenced tests, the model adjustment, the proportion of tests that were sequenced, and the sample size for the time period.


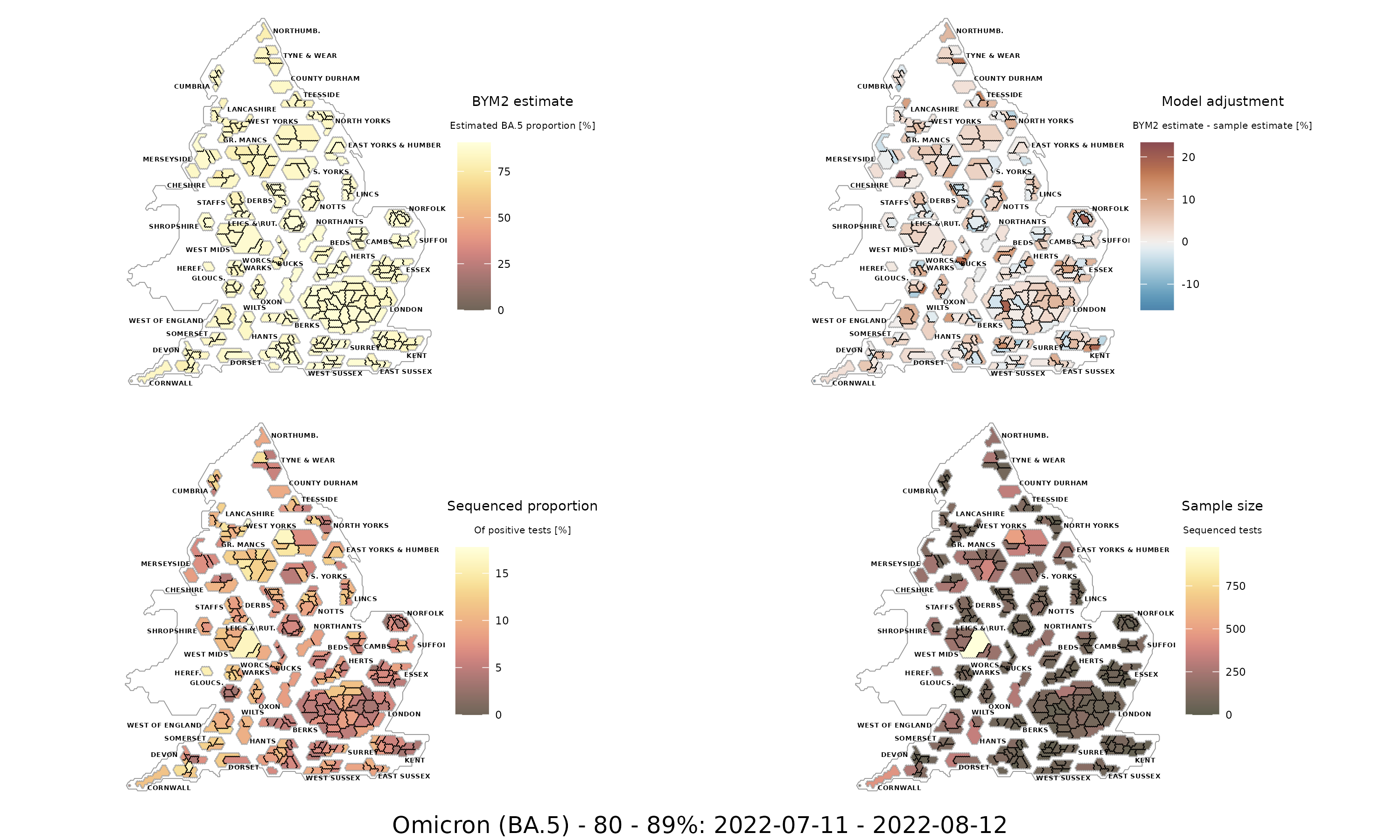


Fig A85. The BYM2 estimated model positivity of the Omicron BA.5 variant as a proportion of sequenced tests, the model adjustment, the proportion of tests that were sequenced, and the sample size for the time period.


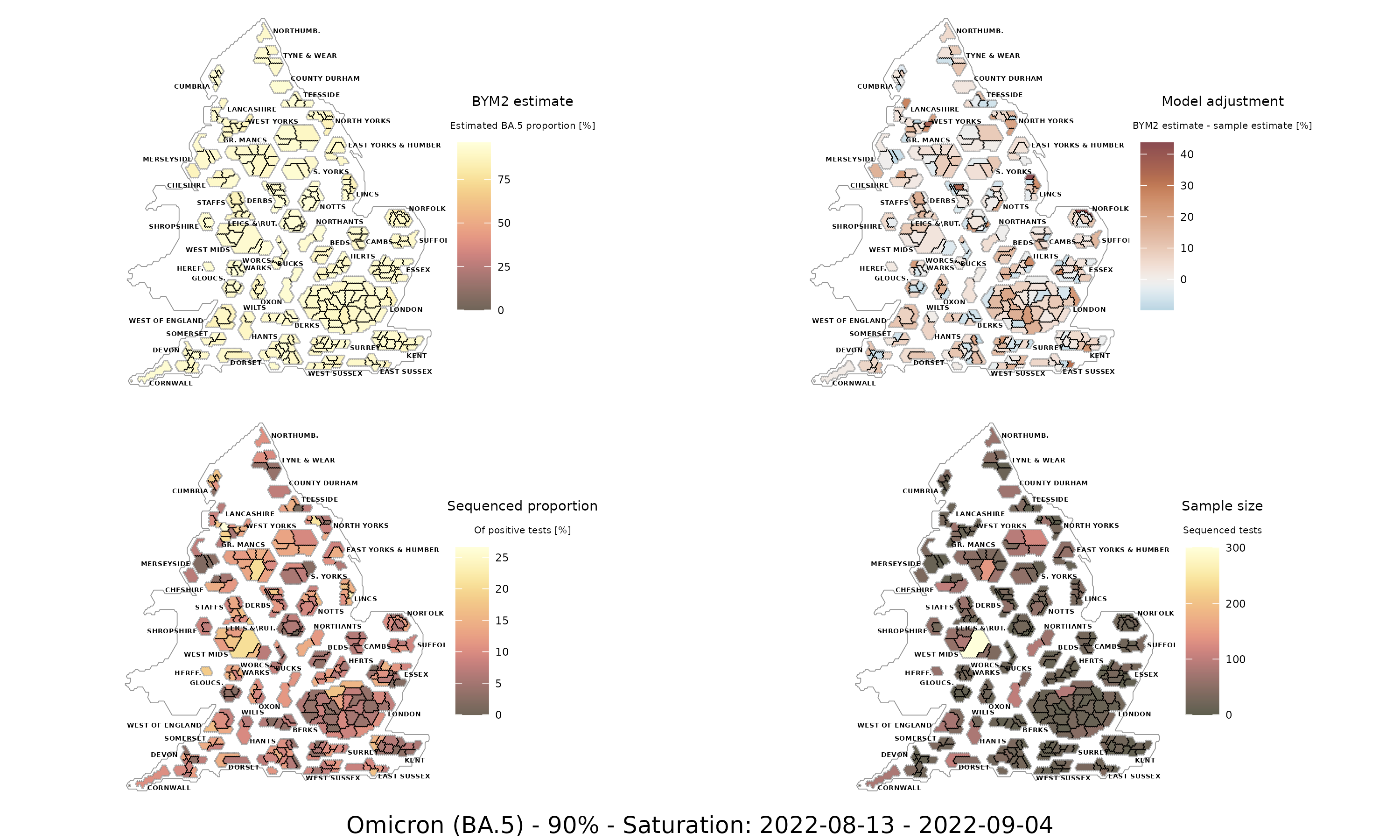


Fig A86. The BYM2 estimated model positivity of the Omicron BA.5 variant as a proportion of sequenced tests, the model adjustment, the proportion of tests that were sequenced, and the sample size for the time period.


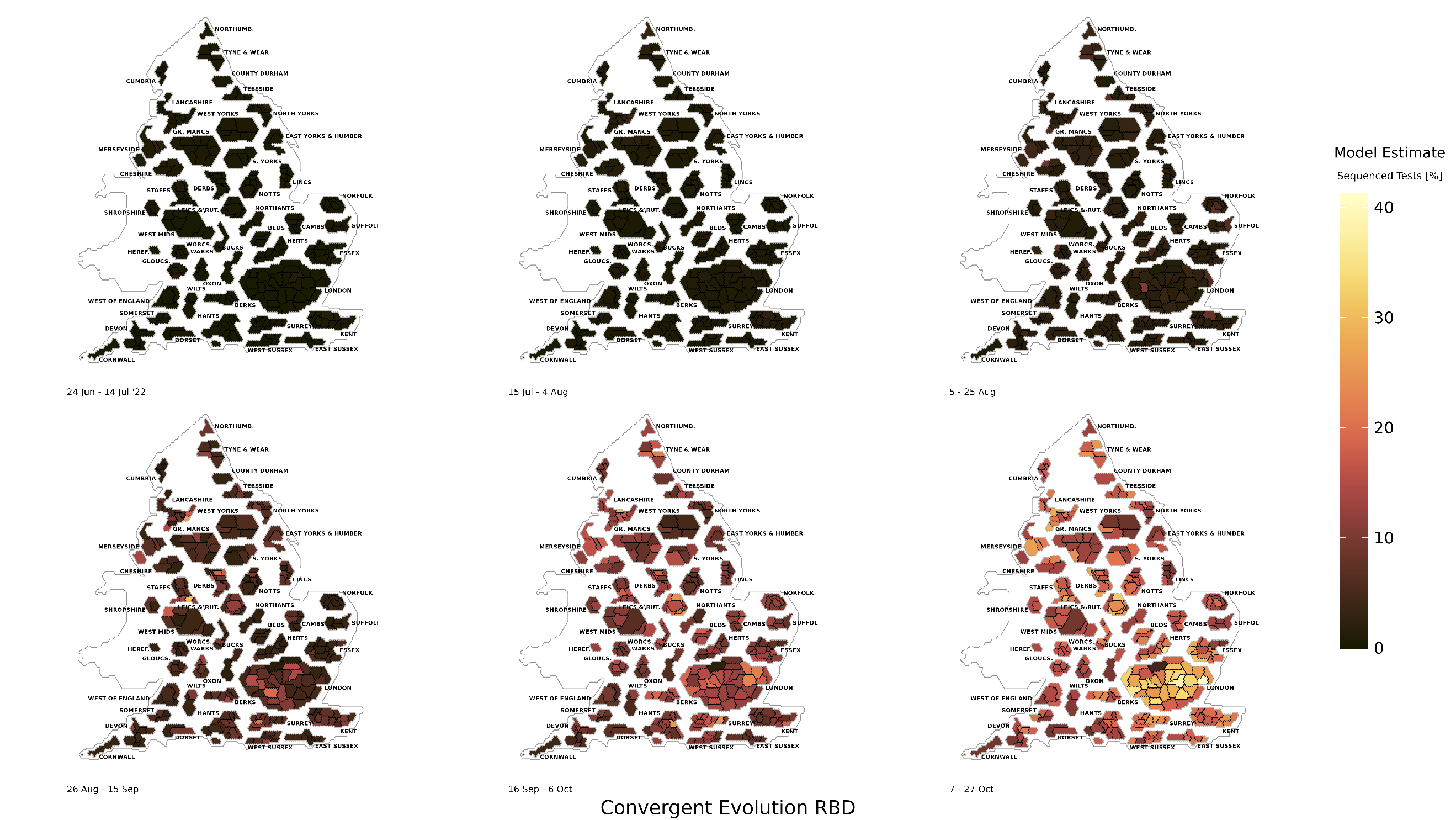


*Fig A87. The BYM2 estimated model positivity of convergent receptor binding domain mutations as a proportion of sequenced tests from 24th June 2022 to 27^th^ October 2022*


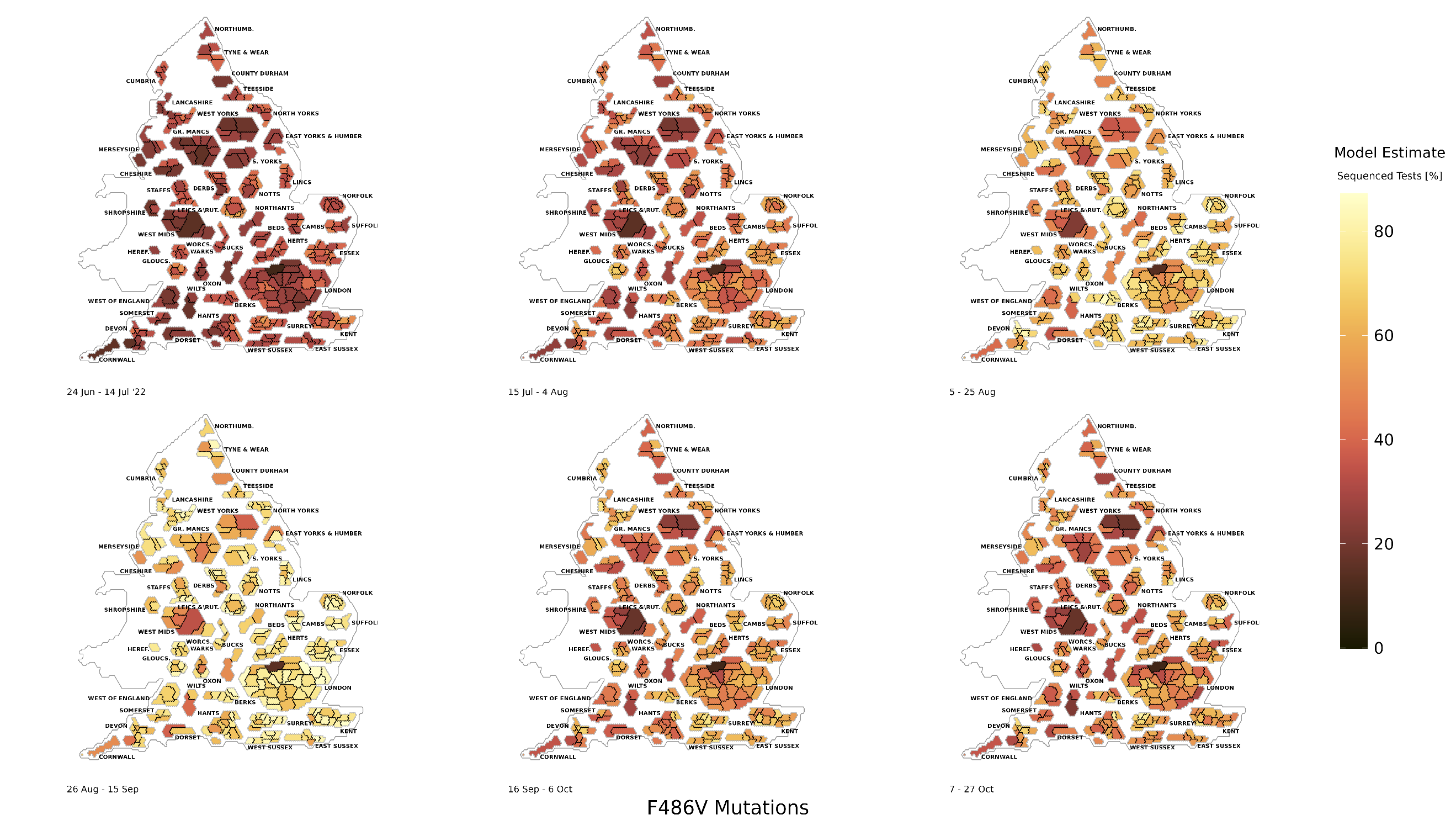


*Fig A88. The BYM2 estimated model positivity of* *F486V receptor binding domain mutation as a proportion of sequenced tests from 24th June 2022 to 27th October 2022*


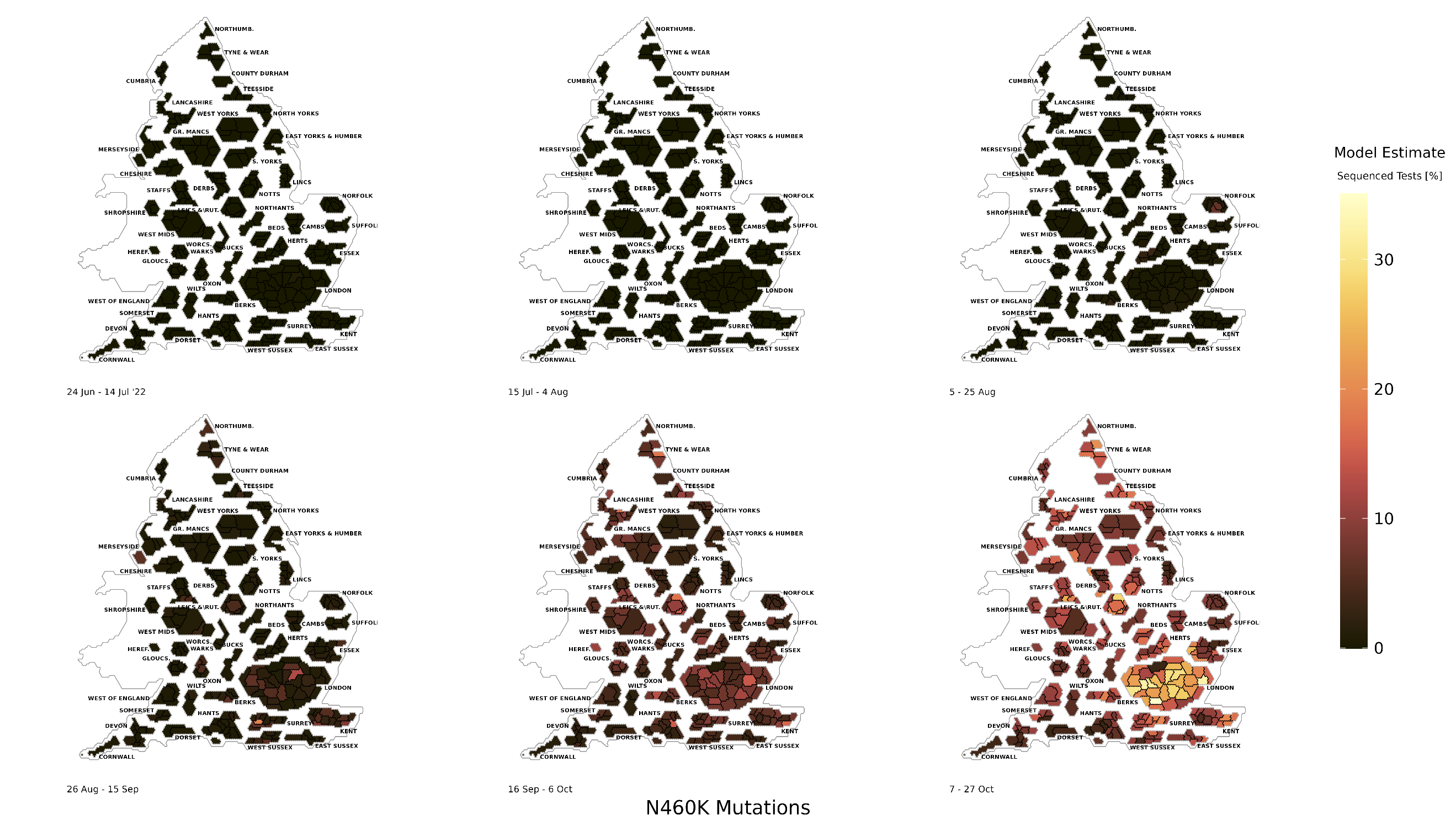


*Fig A89. The BYM2 estimated model positivity of N460K receptor binding domain mutation as a proportion of sequenced tests from 24^th^ June 2022 to 27th October 2022*


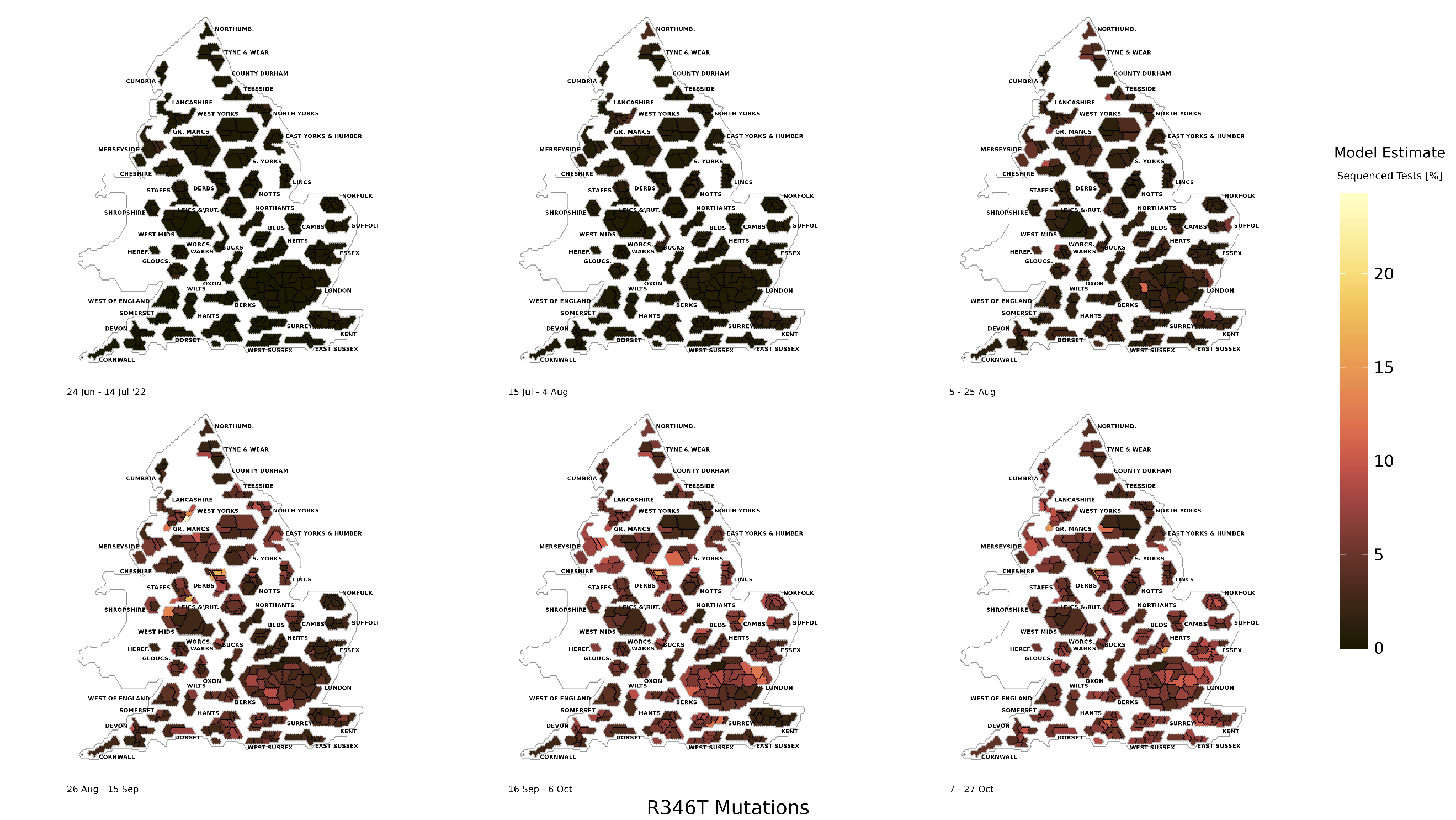


*Fig A90. The BYM2 estimated model positivity of R346T receptor binding domain mutation as a proportion of sequenced tests from 24th June 2022 to 27th October 2022*
