# Supplementary material for: Bayesian spatial modelling of localised SARS-CoV-2 transmission through mobility networks across England
Source: PLoS Comput Biol. 2023 Nov 13;19(11):e1011580. doi: 10.1371/journal.pcbi.1011580 (PMC10756685; doi:10.1371/journal.pcbi.1011580)
Supplement: Attachment — Submitted filename: PLOS Comp Bio Spatial Reviewer Comments.docx [file pcbi.1011580.s003.docx]

Reviewer's Responses to Questions

**Comments to the Authors:
Please note here if the review is uploaded as an attachment.**

Reviewer #1: Many thanks for the excellent study. This tackles an important research question. Mostly the article is clear, but i have the following comments:

- We thank the reviewer for their time and feedback on the study.

line 58: "We have then spatially modelled replacing variant growth through gene targets, whole-genome sequencing, and genotyped RT-PCR tests" doesn't quite read well, suggest a slight change in structure

- We have rephrased this sentence to make it clearer.

line 178: the subscript k is introduced but not explicitly defined to be over spatial unit, this is only implied and may benefit from clarity.

- We have clarified what k means.

about line 200: the authors present the force of infection from other patches in the meta-population but did they consider impact of diurnal flux due to commuting (night-time/daytime mixing is different). would this impact on results?

- We acknowledge that on very granular timescales, diurnal rhythms in the contact network will likely have impacts on transmission. SARS-CoV-2 cases are typically considered to be infectious for around a week, if not longer, and because of this longer timescale we consider it likely that the overall effect of diurnal rhythms is small. For other infections with much smaller infectious periods, it is plausible that diurnal rhythms may need to be accounted for.

Results:

it would be helpful to have a table of parameters being fitted with the prior/posterior estimates in the results. This may be in SI but I couldn't open the SI in the pdf available to conduct review.

- Following reviewer feedback, we have included a table of prior distributions.
- Including the supplementary materials, the model is fitted 137 times. Given this large number of model fits, each producing several parameters of interest, we consider this an unfeasible amount of data to include in this paper as tables. Further, due to complexity of the combined spatial and random effects model, it can be challenging to interpret the effect of several of the key parameters of interest.

Consider % L1 error requires a little thinking about to the lay/general reader.

- We have added an interpretation of L1 error to the paper, line 270.

Reviewer #2: The authors have presented a method for modelling viral transmission using mobility data. The paper is well polished and their use of scenario-based simulation studies to validate their method is commendable. The methodology is well explained and I was able to follow it even as someone with a limited mathematical background. I think this paper is well suited for PLOS Computational Biology, but it still needs some more work.

- We thank the reviewer for their helpful feedback on the study.

Major concern: this is far from the first study on incorporating mobility data into viral transmission models. And yet it is unclear if/how the BYM2 model differs from other methods at achieving this task. I would like to see how this method compares to previous approaches.

- In general, when mobility data is included in epidemic modelling, simulation approaches are typically favoured. This allows modellers to explicitly include epidemic dynamics, while either incorporating or inferring key epidemic parameters. Our focus lies in inferring the spatial pattern of a feature of an epidemic while adjusting for random effects and variations in sample sizes. For emerging variants, estimates of key parameters are likely unknown, and therefore simulation-based approaches would not be appropriate or highly uncertain.

There are examples of using BYM2 type model to understand the spatial structure of features of a disease, such as clinical outcomes or incidence rates. However, these models use adjacency or proximity to inform the spatial relationships between different areas ([2109.10330.pdf (arxiv.org)](https://arxiv.org/pdf/2109.10330.pdf)). Given the complex geographies present in England, this approach is likely unsatisfactory and may lead to poor inferences.

To our knowledge, there are no other BYM2 models that incorporate mobility data to constructing the spatial smoother when inferring the spatial features of an epidemic. In addition, we found relatively few studies that attempt to validate the reliability of their inference using simulation studies. Therefore, comparisons between our model and other approaches are limited by the available literature. Epidemic simulation approaches that assume knowledge of disease parameters are not appropriate for this scenario, and we are unable to find other methodologies for inferring variants prevalence that can account for spatial connectivity.

We have added additional text into the manuscript briefly reviewing other spatial epidemic models.

Code availability: Perhaps I missed it somewhere, but if not I encourage the authors to make their code open source and publicly available. Even if messy, it can still be repurposed by researchers who may want to apply the methods to other parts of the world

- We have released the stan code for the BYM2 model. A significant portion of the codebase will not run without access to a proprietary mobility dataset that we are unable to release this code.

General comment: is this study about England or the UK? The title and body of the article suggests the UK, but all of the maps are of England and there is no mention of Scotland, Ireland, or Wales.

Perhaps it’s too much of a pain to change the title of the paper at this stage, but unless I’m missing something, it should be explicit that this article is about England and not the UK.

- The reviewer is correct that the study focusses only on England. We have amended the title to reflect this comment.

General comment: the maps (esp Fig 4-6) are very low resolution and the labels are difficult to read even when zoomed in

- The images themselves are high resolution, however it appears that when the document was rendered as a pdf much of the image quality was lost. We have partially remedied this by presenting each image on a landscape page, which mitigates the loss in image quality when rendering to pdf that occurs so that the reviewers are able to properly inspect the images. As the images themselves are high resolution, we believe that this will be resolved during the typesetting process.

General comment: from an evolutionary perspective I’m not so keen on the term ‘replacement’, which is used throughout the article. My problem with the term replacement is that the trend is usually variant 1 infects X people per day, and then variant 2 comes along which infects >>X people per day, thereby making variant 1 appear obsolete. The new variant does not simply replace the old one, it also supersedes it. I suggest the term replacement should itself be replaced to better capture underlying evolutionary processes, eg. succeed, supersede, outcompete, outgrow.

- We thank the reviewer for this comment and have removed, where appropriate, replacement however we have retained this term where we think it best captures the process. The rate of replacement is a common term we use when modelling novel variant growth (please see https://www.science.org/doi/10.1126/scitranslmed.abo5395).

General comment: where are the prior distributions? This is a Bayesian analysis, so the priors should be explicated somewhere, perhaps in the SI.

- We have added a table of prior distributions to the manuscript.

Line 3: Here variants of concern are introduced, but on line 89 the article refers to variants of interest. VOC and VOI have different definitions according to the WHO, so it should be clear somewhere if this work and its methodologies make the distinction. Or perhaps use the phrase ‘emerging variants’ where applicable to avoid the need to define these terms.

- We have rephrased line 89, which removes the need to define variant of interest.

Lower tier local authorities -> as a non-Brit I am not familiar with this term, I gather it means district councils? Could you provide a simple description/example of what an LTLA is so the reader does not need to google it

- A description of LTLAs has been included in the data section.

Line 21: subsequently -> consequently

- Fixed

Line 27: “Transmission of this variant… South of England.” -> statement should be supported by a reference

- A reference has been included here.

Line 63: “ethical approval” should start with a new sentence

- Fixed

Line 88: “of which y_i=1 indicate the tested individual was infected with our target variant of interest” -> I think this is erroneous. My understanding is that y_i is the *number* of individuals in population i who were infected with the VoC?

- We have corrected this.

Line 94: what is k? On line 178, A_k is used to denote an area. Is this the same variable k?

- In line 94, k denotes the number of unknown parameters in the generalised linear

Line 117: “if we consider them connected in some sense” -> “if one considers”

- Fixed

Line 278: please provide a reference to support the claim that London, Manchester, and Birmingham were among the first to be infected by Omicron

- We are referring to the results contained in our supplementary materials for the initial spread of Omicron.

Line 405: inconsistent use of present and past-tense: “is found” vs “were small”

- Fixed

Line 411: “Considerable geographical heterogeneity was found for the probability that a test is sequenced or provided gene targets” -> I think this sentence is missing a word somewhere

- We have rephrased this

Line 426: unexpected comma

- Fixed
